# Borylated norbornadiene derivatives: Synthesis and application in Pd-catalyzed Suzuki–Miyaura coupling reactions

**DOI:** 10.3762/bjoc.18.41

**Published:** 2022-04-01

**Authors:** Robin Schulte, Heiko Ihmels

**Affiliations:** 1 Department of Chemistry and Biology, University of Siegen, and Center of Micro- and Nanochemistry and (Bio)Technology (Cμ); Adolf-Reichwein-Str. 2, 57068 Siegen, Germany

**Keywords:** molecular solar thermal system, Pd-mediated catalysis, photochemistry, photoswitches, quadricyclanes

## Abstract

The photochromic norbornadiene/quadricyclane system is among the most promising candidates for molecular solar thermal (MOST) energy storage. As in this context there is still the need for new tailor-made derivatives, borylated norbornadienes were synthesized that may be used as versatile building blocks. Thus, the 4,4,5,5-tetramethyl-2-(bicyclo[2.2.1]heptadien-2-yl)-1,3,2-dioxaborolane was prepared and shown to be a suitable substrate for Pd-catalyzed Suzuki–Miyaura coupling reactions with selected haloarenes. It was demonstrated exemplarily that the novel monosubstituted 2-(1-naphthyl)norbornadiene, that is accessible through this route, is transformed to the corresponding quadricyclane upon irradiation, whereas the back reaction can be accomplished by thermal treatment.

## Introduction

Norbornadiene (**1a**, bicyclo[2.2.1]heptadiene) is a photochromic compound that has recently gained considerable attention because of its ability to store light-energy by the photo-induced intramolecular [2 + 2] cycloaddition to the metastable quadricyclane [1–7]. The latter may be transformed back to the starting norbornadiene in an exothermic process upon heating or irradiation [2,8]. Therefore, the photo-switchable norbornadiene–quadricyclane system has been proposed as a promising basis for a molecular solar thermal storage system (MOST) [9–12]. For that purpose, however, the chromophore of the norbornadiene (**1a**) has to be modified with suitable substituents because the parent system does not operate in the spectrum of the sunlight. Therefore, the efficient and purposeful functionalization of the norbornadiene scaffold is an important and challenging task in this field. Specifically, earlier approaches for the synthesis of norbornadiene derivatives are mainly based on Diels–Alder reactions of cyclopentadiene with alkynes [13–23]. However, since this synthetic route requires strongly electrophilic alkynes, its scope is limited to products that contain at least one electron-acceptor group, such as an ester, a nitrile or amide functionality [13–18,24–25]. At the same time, norbornadiene derivatives are available from metalated substrates. Hence, norbornadiene is deprotonated with the Schlosser base and subsequently trapped by an appropriate electrophile [26–27]. In addition, halogenated norbornadiene derivatives may be metalated in a Li–halogen exchange reaction [27]. In another versatile approach, arylation and alkenylation reactions of the norbornadiene may be accomplished with a Suzuki–Miyaura coupling reaction. In this case, halogenated norbornadienes react with arylboronic acids or their esters to the corresponding aryl-substituted norbornadienes under optimized conditions [28–31]. To the best of our knowledge, however, no borylated norbornadiene derivatives have been employed in Suzuki–Miyaura coupling reactions so far, although this synthetic route appears to be a useful complementary approach to the already established one. Currently, two borylated norbornadienes are already known in literature, namely the dibutyl bicyclo[2.2.1]heptadiene-2-ylboronate, which is not well suited for coupling reactions, and the 2-(3-bromobicyclo[2.2.1]heptadien-2-yl)-4,4,5,5-tetramethyl-1,3,2-dioxaborolane, whose reactivity in Suzuki–Miyaura couplings has not been investigated, so far [32–33]. To explore the suitability of borylated norbornadienes for Suzuki–Miyaura coupling reactions and thus to provide new useful building blocks for the modular construction of norbornadiene derivatives, specifically the so far underrepresented monosubstituted arylnorbornadienes, we examined exemplarily the synthesis of borylated norbornadienes and their Pd-catalyzed Suzuki–Miyaura coupling reactions.

## Results and Discussion

### Synthesis

**Scheme 1:**
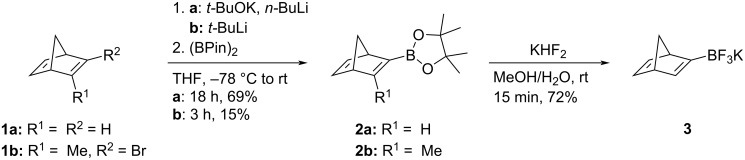
Synthesis of the borylated norbornadienes **2a**,**b** and **3**.

Norbornadiene (**1a**) was deprotonated with the Schlosser base, and the reaction of the resulting metalated norbornadiene with bis(pinacolato)diborone gave the 4,4,5,5-tetramethyl-2-(bicyclo[2.2.1]heptadien-2-yl)-1,3,2-dioxaborolane (**2a**) in 69% yield (Scheme 1). Subsequent treatment of product **2a** with aqueous KHF_2_ solution resulted in the formation of potassium bicyclo[2.2.1]heptadien-2-yltrifluoroborate (**3**) in 72% yield (Scheme 1), which was obtained in sufficient purity (>97%), as indicated by ^1^H NMR spectroscopy; however, satisfactory elemental analysis data could not be obtained for this product. The parent bicyclo[2.2.1]heptadien-2-ylboronic acid could not be obtained at all, as neither the reaction of **2a** with NaIO_4_ and hydrochloric acid, nor the reaction of **2a** with LiOH and subsequent addition of acid (ammonium chloride, hydrochloric acid) gave the desired boronic acid, but led to decomposition, instead. Obviously, the 2-borylated norbornadiene derivatives are acid-labile, so that the synthesis of the boronic acid was not further investigated. In addition, the 4,4,5,5-tetramethyl-2-(3-methylbicyclo[2.2.1]heptadien-2-yl)-1,3,2-dioxaborolane (**2b**) was obtained by conversion of the known 2-bromo-3-methylbicyclo[2.2.1]heptadiene (**1b**) [27] to the metalated intermediate with *t*-BuLi and the following reaction with bis(pinacolato)diborone in 15% overall yield (Scheme 1) [27]. The low yield of this reaction as compared with the ones of resembling reactions of the substrate **1b** [27] may be caused by the unfavorable clash of the sterically demanding borylating reagent and the neighboring methyl group at the reaction center. The novel compounds **2a**, **2b**, and **3** were identified and characterized by NMR spectroscopy (^1^H, ^13^C, COSY, HSQC, HMBC), melting point, and elemental analysis. In addition, the structure of product **2b** was supported by high resolution mass spectrometry (HRMS).

**Scheme 2:**
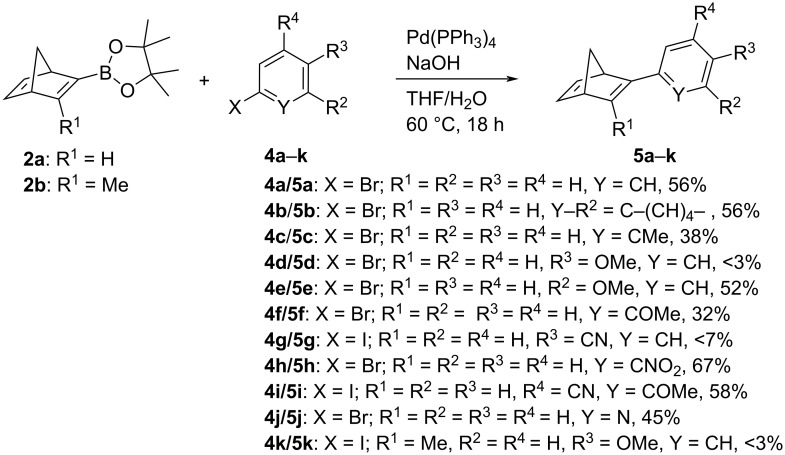
Suzuki–Miyaura coupling reactions of borono-norbornadienes **2a** and **2b** with selected haloarenes **4a**–**k**.

To assess the suitability of the boronic esters **2a** and **2b** to be used as building blocks in Suzuki–Miyaura reactions, the Pd-catalyzed cross-coupling reaction of norbornadiene **2a** and bromobenzene (**4a**) was examined under different conditions (Table 1, Scheme 2). First experiments were conducted with Pd_2_(dba)_3_/(*t*-Bu)_3_PHBF_4_ as catalytic system and CsF as additive in THF at room temperature, because these conditions have been shown to be well-suitable for Suzuki–Miyaura reactions of halogen-substituted norbornadiene derivatives with arylboronic acids [28]. However, under these conditions the coupling reaction of **2a** with **4a** gave the product **5a** in only 37% yield (Table 1, entry 6). The use of PdCl_2_(dppf)·CH_2_Cl_2_ as catalyst with different bases resulted in even lower yields (≤15%; Table 1, entries 2–4). In contrast, the best yield was accomplished with Pd(PPh_3_)_4_ as catalyst and NaOH as base in THF/water at 60 °C giving 2-phenylbicyclo[2.2.1]heptadiene (**5a**) in 56% yield. Under these optimized conditions, the Suzuki–Miyaura coupling reaction of **2a** was also carried out with other representative haloarenes **5b**–**j** to assess the scope and limits of this synthetic route. Thus, the coupling reaction of **2a** with 1-bromonaphthalene (**4b**), 2-bromotoluene (**4c**), 3-bromoanisole (**4e**), 2-bromoanisole (**4f**) 2-bromonitrobenzene (**4h**), 3-iodo-4-methoxybenzonitrile (**4i**), and 2-bromopyridine (**4j**) gave the corresponding 2-arylnorbornadienes in moderate to good yields (32–67%), whereas the reaction with the 4-cyano- and 4-methoxy-substituted halobenzene derivatives **4d** and **4g** gave the corresponding products only in low yield or not at all (Scheme 2). It should be noted, however, that the relatively low yields of some coupling reactions are also caused by the difficult purification of the products, because most of them tend to decompose slowly in solution or during chromatographic work-up [31,34]. The novel compounds **5b**–**j** were identified and fully characterized by NMR spectroscopy (^1^H, ^13^C, COSY, HSQC, HMBC); however, correct elemental analysis was not obtained in the case of the less-stable products **5d**, **5g** and **5j**.

**Table 1:** Pd-catalyzed Suzuki–Miyaura coupling reaction of norbornadiene **2a** and bromobenzene (**4a**) at different reaction conditions.

Entry	Catalyst	Base	Solvent	*T* / °C	Yield **5a** / %^a^

1	Pd(OAc)_2_, PPh_3_	NaOH	THF/H_2_O	60	31
2	PdCl_2_(dppf)·CH_2_Cl_2_	K_2_CO_3_	DME	60	<5%
3	PdCl_2_(dppf)·CH_2_Cl_2_	*t*-BuNH_2_	iPrOH/H_2_O	60	<5%
4	PdCl_2_(dppf)·CH_2_Cl_2_	NaOH	THF/H_2_O	60	15
5	Pd(PPh_3_)_4_	NaOH	THF/H_2_O	60	56
6	Pd_2_(dba)_3_, (*t*-Bu)_3_PHBF_4_	CsF	THF	rt	37

^a^Yield of isolated product **5a** after column chromatography.

The Suzuki–Miyaura coupling reactions of the borononorbornadiene **2a** (Scheme 2) show that this substrate can be used as starting material for the synthesis of monosubstituted norbornadiene derivatives and thus represents a useful complementary method to the already known routes to higher substituted derivatives [28–31]. Although the yields are not very high and may have to be optimized in each specific case, e.g. by variation of the catalyst system, this route is so far the only one reported to get access to a series of compounds from this class of norbornadiene derivatives. So far, only few examples of such monosubstituted compounds have been made available [31,34], and in most cases they have been reported as fairly unstable, which is in agreement with our observations during work-up and storage of the products. At the same time, this approach enabled the isolation and identification of novel stable derivatives as well, such as **5b**, **5c**, and **5i**. But unfortunately, there is no clear trend for a substituent effect on the reaction outcome, as both donor- and acceptor-substituted haloarenes resulted in essentially the same range of yields, i.e. from good yields (e.g., **4b**, **4h**, and **4j**) to failed reactions (**4d** and **4g**). In this context, the different reactivity of the methoxy-substituted arene isomers **4d**–**f** and **4i** is instructive as the *para* isomer did not result in product formation, whereas the *ortho* and *meta*-isomers gave reasonable yields. Hence, there is no obvious impact of the electron-donating or accepting properties of the substituent in this reaction because the methoxy substituent operates as +M donor in the *para* and *ortho*-position and as –I acceptor in the *meta*-position (as well as in the *ortho* position in a not conjugated conformation). Also, steric effects do not seem to have a main impact on the reaction outcome as both examples of *ortho*- and *para*-substituted arene derivatives give similar yields. These results show that there is obviously a fine balance between stereoelectronic and steric effects that determines the outcome of the reaction, as has been shown for the Suzuki–Miyaura reaction [35]. It should be noted that the inverse coupling, i.e., of 2-bromonorbornadiene with arylboronic acid derivatives is also a reasonable and already tested alternative route, that gives very good yields in the case of higher substituted norbornadiene products [28–29]. However, the approach presented herein avoids the use of the significantly more expensive reagents such as the arylboronic acid derivatives and the (*t-*Bu)_3_P ligand. To add to that, the latter is also highly air sensitive and thus requires special handling, but it is essential for a reasonably efficient Pd-mediated coupling of 2-bromonorbornadiene derivatives. In any case, with both complementary methods at hand the synthesis of monoaryl-substituted norbornadiene derivatives is possible in a highly modular approach.

Because the methoxyphenyl-substituted norbornadiene derivative **5c** has been reported already [28], we also attempted, for comparison, its complementary synthesis by the Suzuki–Miyaura coupling reaction of norbornadiene **2b** with 4-iodoanisole (**4k**) (Scheme 2). To our surprise, the product **5k** was not formed in detectable amounts, neither with Pd(PPh_3_)_4_/NaOH, nor with Pd_2_(dba)_3_/(*t*-Bu)_3_PHBF_4_/CsF as catalyst and additive. The ^1^H NMR-spectroscopic analysis of the reaction showed that in both attempts no reaction occurred. It may be concluded that the nucleophilicity of the boronic ester is not sufficient, however, under the employed conditions, it can be hydrolyzed to the more reactive boronic acid. Unfortunately, the latter was not available on preparative scale (see above) so that this aspect could not be clarified with control experiments. At the same time, the coupling reaction may also be sterically hindered by the 3-methyl group of the norbornadiene **2b**, which is in agreement with the low yields of the borylation reaction (see above).

### Photochromism of naphthylnorbornadiene **6b**

**Figure 1:**
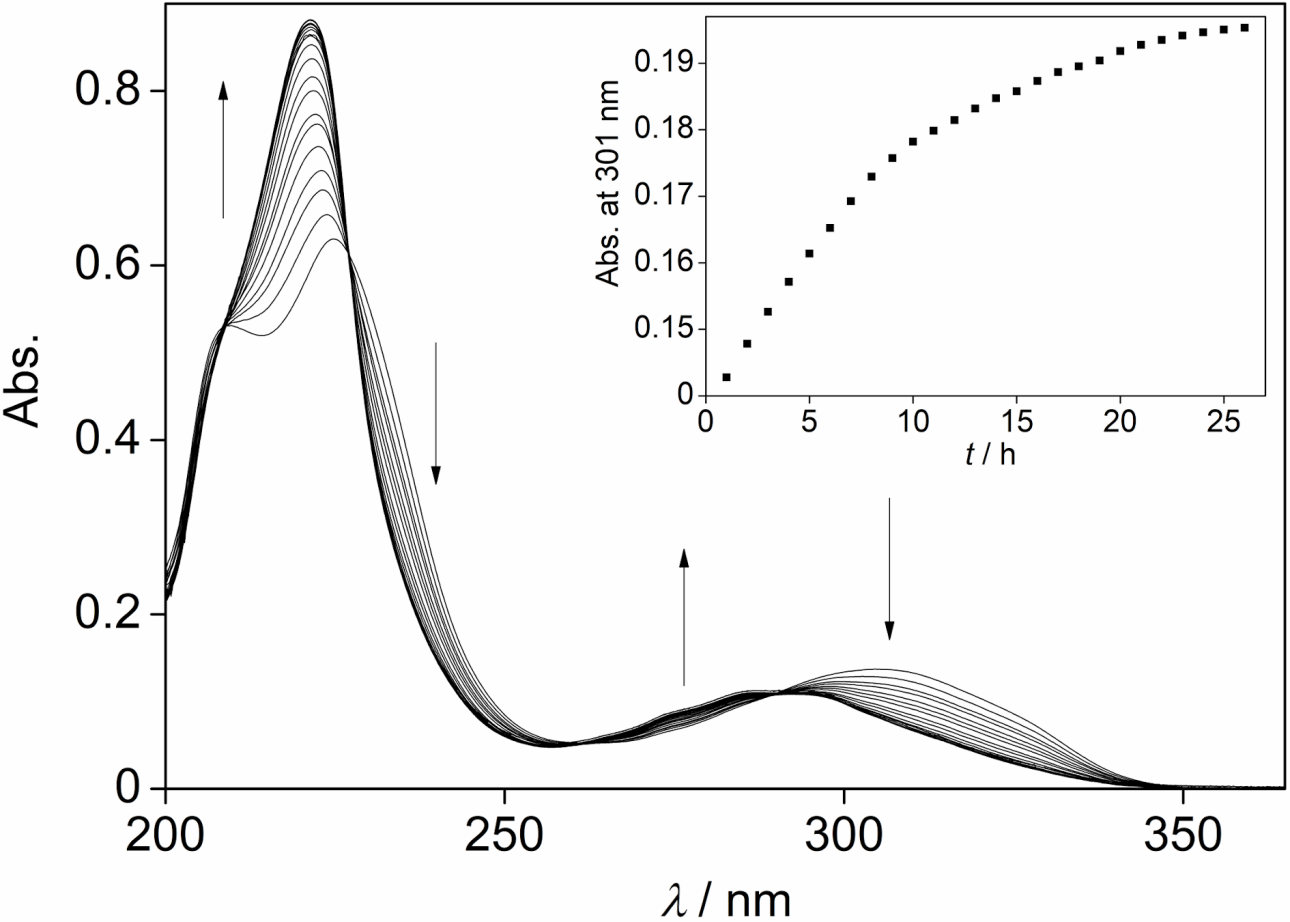
Photometric monitoring of the photoisomerization of 2-(1-naphthyl)norbornadiene (**5b**) in MeCN, *c* = 20 µM, *T* = 20 °C, λ_ex_ = 315 nm. The arrows indicate the development of the absorption bands with reaction time. Inset: Thermally induced back conversion of quadricyclane **6b** into norbornadiene **5b** at 60 °C as monitored by the increase of the norbornadiene absorbance at 301 nm.

**Scheme 3:**
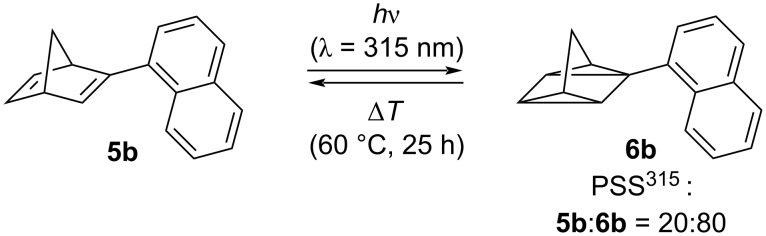
Photo-induced, reversible conversion of the naphthylnorbornadiene **5b** to quadricyclane **6b** in CH_3_CN (PSS^315^: Photostationary state at λ = 315 nm).

The photoisomerization reaction of substrate **5b** was monitored by absorption spectroscopy (Figure 1) and by ^1^H NMR-spectroscopic analysis (see Supporting Information File 1, Figure S51). In MeCN solution, norbornadiene **5b** exhibits the lowest-energy maximum at 305 nm, which is comparable to the reported absorption spectra of known naphthyl-substituted norbornadiene derivatives [36–37]. Irradiation of **5b** at 315 nm leads to a decrease of the absorption band with simultaneous formation of a blue-shifted maximum at 292 nm, which usually indicates the formation of the corresponding quadricyclane **6b** [10–12,29,36]. In addition, the isosbestic points, that developed upon superposition of the spectra obtained during the photoreaction, clearly indicated that only two absorbing species were present in the reaction mixture. The NMR-spectroscopic studies of the latter also confirmed the formation of the quadricyclane product **6b**, because the olefinic ^1^H NMR signals of the norbornadiene at 6.65 ppm, 6.69 ppm, and 6.90 ppm disappeared in favor of signals in the aliphatic region, that are characteristic of the quadricyclane structure [9–12,36]. Moreover, the NMR-spectroscopic analysis of the reaction mixture revealed a photostationary state at λ_ex_ = 315 nm with a norbornadiene/quadricyclane ratio of 20:80. The quadricyclane **6b** could not be transformed back to the norbornadiene **5b** by direct irradiation because of the overlap of its absorption with the one of the norbornadiene. However, upon heating to 60 °C in MeCN solution the norbornadiene was regained after 25 h, as shown by photometric monitoring of the reaction (Scheme 3 and Figure 1, inset). These photochemical properties are comparable to the ones of literature-known naphthyl-substituted norbornadienes, which carry, however, additional acceptor substituents [36–37]. Therefore, these results indicate that the latter substituents may not be necessary to accomplish these photochemical properties and may be even omitted for the sake of molecular mass, that should be as low as possible for MOST applications [38–39].

## Conclusion

In summary, we presented two new borylated norbornadiene derivatives **2a** and **2b** and identified suitable reaction conditions of their Suzuki–Miyaura-coupling reaction with halogenated aromatic substrates. Although the sterically more congested derivative **2b** has significantly suppressed reactivity, the monosubstituted derivative **2a** was shown to be a very useful and complementary building block for the synthesis of monoarylated norbornadiene derivatives that carry no further substituents. It should be noted, however, that the inverse coupling, i.e., of the 2-bromonorbornadiene with arylboronic acid derivatives is also a reasonable and already tested alternative route, but the approach presented herein avoids the use of the usually more expensive arylboronic acid derivatives. In any case, with both complementary methods at hand the synthesis of monoaryl-substituted norbornadiene derivatives is possible in a highly modular approach. Considering the paucity of such derivatives in this field, this synthetic route may pave the way for a new series of promising norbornadiene-based MOST materials.

## Supporting Information

File 1 Experimental protocols and NMR spectra.
