# Supplementary material for: Borylated norbornadiene derivatives: Synthesis and application in Pd-catalyzed Suzuki–Miyaura coupling reactions
Source: Beilstein J Org Chem. 2022 Apr 1;18:368–73. doi: 10.3762/bjoc.18.41 (PMC8978913; doi:10.3762/bjoc.18.41)
Supplement: File 1 — Experimental protocols and NMR spectra. [file Beilstein_J_Org_Chem-18-368-s001.pdf]

BEILSTEIN JOURNAL OF ORGANIC CHEMISTRY

Supporting Information

for

Borylated norbornadiene derivatives: Synthesis and application in Pd-catalyzed Suzuki–Miyaura coupling reactions

Robin Schulte and Heiko Ihmels

Beilstein J. Org. Chem. **2022,** *18,* 368–373. doi:10.3762/bjoc.18.41

Experimental protocols and NMR spectra

License and Terms: This is a supporting information file under the terms of the Creative Commons Attribution License (https://creativecommons.org/licenses/by/4.0). Please note that the reuse, redistribution and reproduction in particular requires that the author(s) and source are credited and that individual graphics may be subject to special legal provisions.
The license is subject to the *Beilstein Journal of Organic Chemistry* terms and conditions: (https://www.beilstein-journals.org/bjoc/terms)

Table of contents

1 Experimental section S2

1.1 Materials S2

1.2 Equipment S2

1.3 Methods S2

1.4 Synthesis S2

1.5 Parameters of the photometric experiments S8

2 NMR spectra S9

3 References S34

1 Experimental section

1.1 Materials

Commercially available reagents were obtained from Acros Organics (*n*-BuLi, *t*-BuLi, CsF), Alfa Aesar [norbornadiene, $(BPin)_2$, 1-bromonaphthalene, 2-bromoanisole, 3-bromoanisole, 4-bromoanisole, 2-bromotoluene, 2-bromopyridine, 1-bromo-2-nitrobenzene, 4-iodobenzonitrile, 4-iodoanisole, $(t\text{-Bu})_3PHBF_4$), Carl Roth [$Pd(PPh_3)_4$, $Pd(dba)_3$] and Merck (*t*-BuOK, bromobenzene). Technical-grade solvents were distilled prior to use. THF was stirred under reflux with a Na wire and distilled prior to use. Column chromatography was carried out with silica gel 60 M (0.0063–0.25 mm) from Macherey Nagel GmbH & Co. KG. 2,3-Dibromonorbornadiene, 2-bromo-3-methylnorbornadiene and 3-iodo-4-methoxybenzonitrile were synthesized according to literature protocols [1,2].

1.2 Equipment

The NMR spectra were recorded on a JEOL ECZ 500 spectrometer (^{1}H: 500 MHz, ^{13}C: 125 MHz, 22 °C) or on a Varian 600 ASC (^{1}H: 600 MHz, ^{13}C: 150 MHz). The spectra were referenced to DMSO-d_6 [δ(^{1}H) = 2.05 ppm, δ(^{13}C) = 39.5 ppm], $CDCl_3$ [δ(^{1}H) = 7.26 ppm, δ(^{13}C) = 77.2 ppm], MeCN [δ(^{1}H) = 1.94 ppm, δ(^{13}C) = 118.3 ppm] or acetone-d_6) [δ(^{1}H) = 2.05 ppm, δ(^{13}C) = 29.8 ppm, 206.0 ppm] and processed with the MestReNova software. The melting points were determined with a BÜCHI 545 (Büchi, Flawil, CH) and are uncorrected. Elemental analysis data were determined by Rochus Breuer (Organic Chemistry I, University of Siegen) on a HEKAtech EUROEA combustion analyzer. Mass spectra were recorded with an orbitrap mass spectrometer Thermo Fisher Exactive (driving current: 4 KV, capillary temperature: 250 °C, capillary voltage: 32.5 V, injection rate: 15 µL/min, scanning range: 100–750 *m*/*z*, resolution: ultra-high) and processed with the Xcalibur software. The absorption spectra were measured on a Varian Cary 100 Bio absorption spectrometer with Hellma quartz glass cuvettes 115 F-QS (layer thickness d = 10 mm). The measurements were recorded at a temperature of T = 20 °C as adjusted with a thermostat if not stated otherwise. The irradiation of **5b** was performed with a LUMOS 43 from Atlas Photonics.

1.3 Methods

Reaction solutions were stirred with a magnetic stirring bar. Reaction temperatures refer to the medium that surrounded the reaction vessel. Solvents were usually removed under reduced pressure at 40–50 °C with a rotatory evaporator. Room temperature (rt) was approximately 22 °C. Air- and/or water-sensitive reactions were carried out under inert atmosphere with Schlenk equipment.

1.4 Synthesis

4,4,5,5-Tetramethyl-2-(bicyclo[2.2.1]heptadien-2-yl)-1,3,2-dioxaborolane (**2a**)

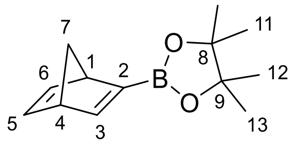

Under argon-gas atmosphere norbornadiene (1.5 g, 16 mmol, 1.6 mL) was added to a mixture of *t*-BuOK (881 mg, 7.87 mmol) in anhydrous THF (30 mL) at −78 °C, and the solution was stirred for 5 min at this temperature. Then, *n*-BuLi (7.9 mmol, 3.2 mL) was added dropwise at −78 °C over a period of 30 min and the resulting yellow solution was stirred for 30 min at −40 °C. After the mixture was cooled to −78 °C, a solution of bis(pinacolato)diborone (2.00 g,

7.87 mmol) in anhydrous THF (25 mL) was added dropwise. The reaction mixture was stirred for 1 h at −78 °C, followed by stirring for 18 h at room temperature. Water (100 mL) was added and the aqueous layer was extracted with EtOAc (3 × 80 mL). The combined organic layers were washed with brine (1 × 100 mL), dried with sodium sulfate, and filtered. The solvent was removed under reduced pressure and the crude product (2.23 g) was purified by column chromatography (hexane/EtOAc 9:1, *v*:*v*, R_f = 0.63) to give **2a** as a colorless solid (1.18 g, 5.41 mmol, 69%); mp 47–48 °C. – ^{1}H-NMR (500 MHz, $CDCl_3$): δ = 1.27 (s, 12H, 10-H, 11-H, 12-H, 13-H), 1.94–1.98 (m, 2H, 7-H), 3.65 (br. s, 1H, 4-H), 3.81 (br. s, 1H, 1-H), 6.66 (dd, 1H, ^3J = 3.2 Hz, ^3J = 4.5 Hz, 5-H), 6.80 (dd, 1H, ^3J = 3.2 Hz, ^3J = 4.9 Hz, 6-H), 7.60 (d, 1H, ^3J = 2.9 Hz, 3-H). – ^{13}C-NMR (125 MHz, $CDCl_3$): δ = 25.0 (C10, C 11, C12, C13), 52.3 (C4), 52.4 (C1), 75.2 (C7), 88.2 (C8, C9), 141.7 (C5), 144.4 (C6), 146.2 (C2), 161.3 (C3). – El. Anal. for $C_{13}H_{19}BO_2$: calc. (%): C 71.59, H 8.78, found (%): C 71.72, H 8.85.

Potassium bicyclo[2.2.1]heptadien-2-yl trifluoroborate (**3**)

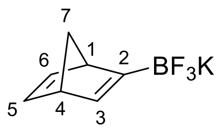

To a solution of **2a** (500 mg, 2.29 mmol) in MeOH (7.0 mL) was added an aqueous solution of KHF_2 (4.5 M, 13 mmol, 2.9 mL), and the resulting suspension was stirred for 30 min at room temperature. The solvent was removed under reduced pressure (40 mbar, 55 °C), and the residue was diluted with hot acetone (20 mL). After filtration, the solvent of the filtrate was removed under reduced pressure and the residue was treated with Et_2O (50 mL). The precipitate was filtered off and washed with Et_2O (20 mL) to yield **3** as a white solid (326 mg, 1.65 mmol, 72%); mp 220–225 °C. – ^{1}H-NMR (500 MHz, acetone-d_6): δ = 1.69-1.74 (m, 2H, 7-H), 3.35 (br. s, 1H, 4-H), 3.55 (br. s, 1H, 1-H), 6.37 (br. s, 1H, 3-H), 6.51–6.55 (m, 1H, 5-H), 6.60–6.63 (m, 1H, 6-H). – ^{13}C-NMR (125 MHz, acetone-d_6): δ = 51.2 (C4), 53.7 (C1), 74.2 (C7), 140.3 (C3), 142.8 (C5), 144.8 (C6).

4,4,5,5-Tetramethyl-2-(3-methylbicyclo[2.2.1]heptadien-2-yl)-1,3,2-dioxaborolane (**2b**)

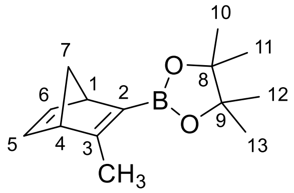

t-BuLi (1.90 M, 7.50 mmol, 3.95 mL) was added dropwise under argon gas atmosphere to a solution of 2-bromo-3-methylnorbornadiene (694 mg, 3.75 mmol) in anhydrous THF (15 mL) at −78 °C, and the resulting yellow solution was stirred for 40 min at −78 °C. A solution of bis(pinacolato)diborone (2.00 g, 7.87 mmol) in anhydrous THF (10 mL) was added at –78 °C and the colorless solution was stirred for 45 min at −78 °C and subsequently for 1 h at room temperature. Water (25 mL) was added, the layers were separated, and the aqueous layer was extracted with Et_2O (3 × 30 mL). The combined organic layers were washed with water (1 × 50 mL) and brine (1 × 50 mL), dried with sodium sulfate, and filtered. The solvent was removed under reduced pressure and the crude product was purified by column chromatography (hexane/EtOAc 9:1, *v*:*v*, R_f = 0.59) to give **2b** (130 mg, 560 µmol, 15%) as a corlorless liquid. ^{1}H-NMR (600 MHz, $CDCl_3$): 1.25 (s, 12H, 10-H, 11-H, 12-H, 13-H), 1.86–1.87 (m, 1H, 7-H), 1.92–1.93 (m, 1H, 7-H), 2.12 (s, 3H, CH_3), 3.29 (br. s, 1H, 4-H), 3.75 (br. s, 1H, 1-H), 6.68 (dd, 1H, ^3J = 3.1 Hz, ^3J = 4.8 Hz, 5-H), 6.79 (dd, 1H, ^3J = 3.1 Hz, ^3J = 4.9 Hz, 6-H). – ^{13}C-NMR (150 MHz, $CDCl_3$): 18.4 (CH_3C), 25.1 (C10, C11, C12, C13), 52.9 (C1), 57.8 (C4), 72.6 (C7), 82.7 (C8, C9), 140.3 (C5), 144.9 (C6), 174.4 (C3). – HRMS for $C_{14}H_{21}BO_2$: calcd. 232.1629 $[M + H]^+$; found 232.1701 $[M + H]^+$. – El. Anal. for $C_{14}H_{21}BO_2$: calc. (%): C 72.44, H 9.12, found (%): C 72.55, H 9.27.

General procedure A (GP A) for Pd-catalyzed Suzuki–Miyaura coupling reaction of borylated norbornadiene derivatives with aryl halides

Under anaerobic conditions, an aqueous NaOH solution (1.0 M, 1.4 mmol, 1.4 mL, 3.3 molar equiv) was added to a suspension of the aryl halide (418 µmol, 1.00 molar equiv), norbornadiene **2a** or **2b** (459 µmol, 1.10 equiv) and $Pd(PPh_3)_4$ (20.9 µmol, 5 mol %) in THF (4.0 mL). The resulting suspension was stirred for 18 h at 60 °C. After cooling the reaction mixture to room temperature, it was diluted with Et_2O (50 mL), dried with sodium sulfate and filtered. The solvents were removed under reduced pressure and the remaining crude was purified by column chromatography.

General procedure B (GP B) for Pd-catalyzed Suzuki–Miyaura coupling reaction of borylated norbornadiene derivatives with aryl halides

Under an argon gas atmosphere, a suspension of aryl halide (505 µmol, 1.00 equiv), norbornadiene **2a** or **2b** (505 µmol, 1.00 equiv), CsF (1.67 mmol, 3.30 equiv), $Pd_2(dba)_3$ (2 mol %) and $(t\text{-Bu})_3PHBF_4$ (1 mol %) in oxygen-free THF (4.0 mL) was stirred for 18 h at room temperature. The solvent was removed under reduced pressure and the remaining crude was purified by column chromatography.

2-Phenylnorbornadiene (**5a**)

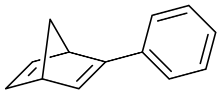

1. Bromobenzene (**4a**, 32.7 mg, 208 µmol, 22.0 µL) and **2a** (50.0 mg, 229 µmol) were treated according to GP A. The crude product was purified by column chromatography (hexane, R_f = 0.37) to provide **5a** as a colorless liquid (19.5 mg, 116 µmol, 56%). NMR data were identical to those reported in the literature [3].

2. Bromobenzene (**4a**, 79.3 mg, 505 µmol, 53.0 µL) and **2a** (111 mg, 505 µmol) were treated according to GP B. The crude product was purified by column chromatography (hexane, R_f = 0.37) to provide **5a** as a colorless liquid (31.0 mg, 184 µmol, 37%). NMR data were identical to those reported in the literature [3].

2-(1-Naphthyl)-norbornadiene (**5b**)

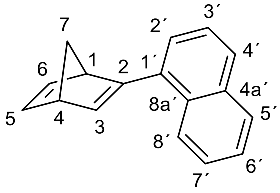

1-Bromonaphthalene (**4b**, 86.5 mg, 418 µmol, 59.0 µL) and **2a** (100 mg, 459 µmol) were treated according to GP A. The crude product was purified by column chromatography (hexane, R_f = 0.61) to provide **5b** as a colorless liquid (51.0 mg, 233 µmol, 56%). – ^{1}H-NMR (500 MHz, $CDCl_3$): δ = 2.18 (d, 1H, ^2J = 6.0 Hz, 7-H), 2.39 (d, 1H, ^2J = 6.0 Hz, 7-H), 3.84–3.85 (m, 2H, 1-H, 4-H), 6.83 (d, 1H, ^3J = 3.0 Hz, 3-H), 6.89 (dd, 1H, ^3J = 5.2 Hz, ^3J = 3.0 Hz, 5-H), 7.07 (dd, ^3J = 5.1 Hz, ^3J = 3.0 Hz, 6-H), 7.28 (d, 1H, ^3J = 7.1 Hz, 2´-H), 7.42 (dd, 1H, ^3J = ^3J = 8.0 Hz, 3´-H), 7.46–7.49 (m, 2H, 6´-H, 7´-H), 7.72 (d, 1H, ^3J = 8.2 Hz, 4´-H), 7.83–7.86 (m, 1H, 5´-H), 8.12–8.15 (m, 1H, 8´-H). – ^{13}C-NMR (125 MHz, $CDCl_3$): δ = 51.6 (C4), 56.2 (C1), 73.5 (C7), 123.3 (C2´), 125.5 (C3´), 125.8 (C6´), 125.9 (C7´), 126.0 (C8´), 127.3 (C4´),

128.5 (C5´), 131.2 (C8a´), 134.0 (C4a´), 136.3 (C1´), 140.1 (C3), 142.8 (C6), 143.4 (C5), 156.1 (C2). – El. Anal. for $C_{17}H_{14}$: calc. (%): C 93.54, H 6.46, found (%): C 93.82, H 6.45.

2-(2-Methylphenyl)-norbornadiene (**5c**)

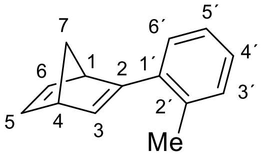

1-Bromo-2-methylbenzene (**4c**, 141 mg, 824 µmol, 99.3 µL) and **2a** (198 mg, 906 µmol) were treated according to GP A. The crude product was purified by column chromatography (hexane, R_f = 0.57) and subsequent size exclusion chromatography (Sephadex, LH20, MeOH) to provide **5c** as a colorless liquid (57.3 mg, 315 µmol, 38%). – ^{1}H-NMR (500 MHz, $CDCl_3$): δ = 2.08 (dt, 1H, ^2J = 5.9 Hz, ^3J = 1.5 Hz, 7-H), 2.19 (dt, 1H, ^2J = 5.9 Hz, ^3J = 1.6 Hz, 7-H), 2.35 (s, 3H, Me), 3.72–3.75 (m, 1H, 4-H), 3.77–3.80 (m, 1H, 1-H), 6.65 (d, 1H, ^3J = 3.2 Hz, 3-H), 6.83 (dd, 1H, ^3J = 4.3 Hz, ^3J = 3.0 Hz, 5-H), 6.96 (dd, 1H, ^3J = 5.0 Hz, ^3J = 3.0 Hz, 6-H), 7.12–7.16 (m, 1H, 4´H), 7.18–7.21 (m, 3H, 3´H, 5´H, 6´H). – ^{13}C-NMR (125 MHz, $CDCl_3$): δ = 21.6 (Me), 51.3 (C4), 55.1 (C1), 73.0 (C7), 125.7 (C5´), 126.6 (C4´), 126.7 (C6´), 130.6 (C3´), 135.4 (C2´), 137.1 (C1´), 139.3 (C3), 142.6 (C6), 143.5 (C5), 156.8 (C2). – El. Anal. for $C_{14}H_{14}$: calc. (%): C 92.26, H 7.74, found (%): C 92.54, H 7.75.

2-(4-Methoxyphenyl)-norbornadiene (**5d**)

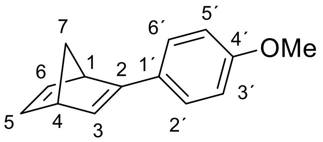

1-Bromo-4-methoxybenzene (**4d**, 94.4 mg, 505 µmol, 63.4 µL) and **2a** (121 mg, 556 µmol) were treated according to GP A. ^{1}H NMR-spectroscopic analysis of the reaction mixture showed that the product was formed, but decomposed rapidly under the reaction conditions.

2-(3-Methoxyphenyl)-norbornadiene (**5e**)

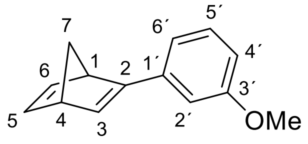

1-Bromo-3-methoxybenzene (**4e**, 142 mg, 758 µmol, 96.1 µL) and **2a** (182 mg, 834 µmol) were treated according to GP A. The crude product was purified by column chromatography (hexane/EtOAc 9:1, *v*:*v*, R_f = 0.65) to provide **5e** as a colorless liquid (78.0 mg, 394 µmol, 52%). – ^{1}H-NMR (500 MHz, $CDCl_3$): δ = 2.10 (dt, 1H, ^2J = 6.3 Hz, ^3J = 1.8 Hz, 7-H), 2.14 (dt, 1H, ^2J = 6.3 Hz, ^3J = 1.7 Hz, 7-H), 3.69–3.72 (m, 1H, 4-H), 3.82 (s, 3H, OMe), 3.90–3.92 (m, 1H, 1-H), 6.75 (dd, 1H, ^3J = 8.2 Hz, ^4J = 2.6 Hz, 4´-H), 6.79 (dd, 1H, ^3J = 5.2 Hz, ^3J = 3.0 Hz, 5-H), 6.89–6.91 (m, 2H, 3-H, 6-H,), 6.95 (t, 1H, ^4J = 1.6 Hz, 2´-H), 7.01 (dt, 1H, ^3J = 7.8 Hz, ^4J = 1.1 Hz, 6´-H), 7.24 (t, 1H, ^3J = 8.0 Hz, 5´-H). – ^{13}C-NMR (125 MHz, $CDCl_3$): δ = 51.0 (C4), 51.7 (C1), 55.3 (OMe), 72.5 (C7), 110.4 (C2´), 112.5 (C4´), 117.5 (C6´), 129.5 (C5´), 136.7 (C3), 141.9 (C1´), 142.1 (C6), 143.5 (C5), 156.6 (C2), 159.9 (C3´).). – El. Anal. for $C_{14}H_{14}O$ x 0.5 H_2O: calc. (%): C 81.13, H 7.29, found (%): C 81.01, H 7.05.

2-(2-Methoxyphenyl)-norbornadiene (**5f**)

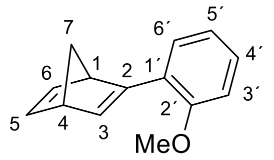

1-Bromo-2-methoxybenzene (**4f**, 142 mg, 758 µmol, 95.0 µL) and **2a** (182 mg, 834 µmol) were treated according to GP A. The crude product was purified by column chromatography (hexane/EtOAc 9:1, *v*:*v*, R_f = 0.69) to provide **5f** as a colorless liquid (48.2 mg, 243 µmol, 32%). – ^{1}H-NMR (500 MHz, $CDCl_3$): δ = 2.05 (dt, 1H, ^2J = 6.0 Hz, ^3J = 1.5 Hz, 7-H), 2.12 (dt, 1H, ^2J = 6.0 Hz, ^3J = 1.9 Hz, 7-H), 3.70–3.72 (m, 1H, 4-H), 3.86 (s, 3H, OMe), 3.97–4.00 (m, 1H, 1-H), 6.79 (dd, 1H, ^3J = 5.1 Hz, ^3J = 3.0 Hz, 5-H), 6.87 (dd, 1H, ^3J = 8.3 Hz, ^4J = 1.1 Hz, 3´-H), 6.91–6.97 (m, 2H, 6-H, 5´H), 7.11 (d, 1H, ^3J = 3.2 Hz, 3-H), 7.18 (ddd, 1H, ^3J = 9.9 Hz, ^3J = 8.3 Hz, ^4J = 1.9 Hz, 4´-H), 7.33 (dd, 1H, ^3J = 7.6 Hz, ^4J = 1.9 Hz, 6´-H). – ^{13}C-NMR (125 MHz, $CDCl_3$): δ = 51.1 (C4), 53.2 (C1), 55.3 (OMe), 72.0 (C7), 110.8 (C3´), 120.6 (C5´), 125.8 (C1´), 127.2 (C6´), 127.8 (C4´), 139.8 (C3), 142.5 (C6), 143.6 (C5), 153.3 (C2), 156.9 (C2´). – El. Anal. for $C_{14}H_{14}O$ x 0.5 H_2O: calc. (%): C 81.13, H 7.29, found (%): C 80.93, H 6.97.

2-(4-Cyanophenyl)-norbornadiene (**5g**)

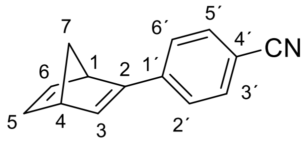

1-Iodo-4-cyanobenzene (**4g**, 178 mg, 777 µmol) and **2a** (187 mg, 855 µmol) were treated according to GP A. The crude product was purified by column chromatography (hexane/EtOAc 9:1, *v*:*v*, R_f = 0.49) to provide **5g** as a yellow oil (10.5 mg, 54.4 µmol, 7%), which was still contaminated with starting material and decomposition products according to ^{1}H NMR-spectroscopic analysis.

2-(2-Nitrophenyl)-norbornadiene (**5h**)

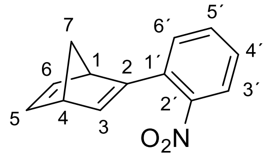

1-Bromo-2-nitrobenzene (**4h**, 142 mg, 704 µmol) and **2a** (169 mg, 774 µmol) were treated according to GP A. The crude product was purified by column chromatography (hexane/EtOAc 9:1, *v*:*v*, R_f = 0.51) to provide **5h** as a colorless liquid (100.0 mg, 469 µmol, 67%). – ^{1}H-NMR (500 MHz, $CDCl_3$): δ = 2.09 (dt, 1H, ^2J = 6.0 Hz, ^3J = 1.4 Hz, 7-H), 2.29 (dt, 1H, ^2J = 6.0 Hz, ^3J = 1.5 Hz, 7-H), 3.50–3.53 (m, 1H, 1-H), 3.70–3.73 (m, 1H, 4-H), 6.77–6.80 (m, 2H, 3-H, 5-H), 6.91 (dd, 1H, ^3J = 5.0 Hz, ^3J = 2.9 Hz, 6-H), 7.26 (dd, 1H, ^3J = 7.8 Hz, ^4J = 1.4 Hz, 6´-H), 7.33–7.37 (m, 1H, 4´-H), 7.47 (td, 1H, ^3J = 7.6 Hz, ^4J = 1.3 Hz, 5´-H), 7.79 (dd, 1H, ^3J = 8.1 Hz, ^4J = 1.3 Hz, 3´-H). – ^{13}C-NMR (125 MHz, $CDCl_3$): δ = 50.9 (C4), 54.5 (C1), 73.5 (C7), 124.1 (C3´), 127.7 (C4´), 129.9 (C6´), 132.5 (C1´), 132.9 (C5´), 140.6 (C3), 142.3 (C5), 142.9 (C6), 147.7 (C2), 154.0 (C2´). – El. Anal. for $C_{14}H_{14}NO_2$ x 0.5 H_2O: calc. (%): C 70.26, H 5.44, N 6.30, found (%): C 70.74, H 5.29, N 6.07.

2-(2-Methoxy-5-cyanophenyl)-norbornadiene (**5i**)

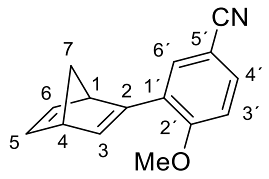

3-Iodo-4-methoxybenzonitrile (**4i**, 174 mg, 673 µmol) and **2a** (161 mg, 740 µmol) were treated according to GP A. The crude product was purified by column chromatography (hexane/EtOAc 8:2, *v*:*v*, R_f = 0.40) to provide **5i** as a yellow liquid (86.4 mg, 387 µmol, 58%). – ^{1}H-NMR (500 MHz, $CDCl_3$): δ = 2.08 (dt, 1H, ^{2}J = 6.1 Hz, ^{3}J = 1.5 Hz, 7-H), 2.12 (dt, 1H, ^{2}J = 6.1 Hz, ^{3}J = 1.5 Hz, 7-H), 3.72–3.75 (m, 1H, 4-H), 3.92 (br. s, 4H, 1-H, OMe), 6.80 (dd, 1H, ^{3}J = 5.1 Hz, ^{3}J = 3.2 Hz, 5-H), 6.89–6.93 (m, 2H, 6-H, 3´H), 7.21 (d, 1H, ^{3}J = 3.3, 3-H), 7.48 (dd, 1H, ^{3}J = 8.5 Hz, ^{4}J = 2.2 Hz, 4´-H), 7.59 (d, 1H, ^{4}J = 2.2 Hz, 6´-H). – ^{13}C-NMR (125 MHz, $CDCl_3$): δ = 51.3 (C4), 53.0 (C1), 55.7 (OMe), 72.2 (C7), 104.1 (C5´), 111.2 (C3´), 119.6 (C2´), 127.1 (CN), 130.8 (C6´), 132.0 (C3´), 142.3 (C6), 142.9 (C3), 143.6 (C5), 151.5 (C1), 160.2 (C1´). – El. Anal. for $C_{15}H_{13}NO_2$ x 0.5 H_2O: calc. (%): C 77.56, H 6.08, N 6.03 found (%): C 77.59, H 5.66, N 5.94.

2-(2-Pyridyl)-norbornadiene (**5j**)

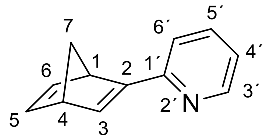

2-Bromopyridine (**4j**, 140 mg, 888 µmol, 86.4 µL) and **2a** (213 mg, 977 µmol) were treated according to GP A. The crude product was purified by column chromatography (hexane/EtOAc 9:1, *v*:*v*, R_f = 0.41) to provide **5j** as a brown liquid (67.3 mg, 398 µmol, 45%). – ^{1}H-NMR (500 MHz, $CDCl_3$): δ = 2.15 (dt, 1H, ^{2}J = 6.2 Hz, ^{3}J = 1.6 Hz, 7-H), 2.19 (dt, 1H, ^{2}J = 6.2 Hz, ^{3}J = 1.6 Hz, 7-H), 3.74–3.78 (m, 1H, 4-H), 4.19–4.22 (m, 1H, 1-H), 6.80 (dd, 1H, ^{3}J = 5.0 Hz, ^{3}J = 2.9 Hz, 5-H), 6.96 (dd, 1H, ^{3}J = 5.0 Hz, ^{4}J = 2.9 Hz, 6-H), 7.06–7.10 (m, 1H, 4´H), 7.28–7.31 (m, 1H, 3-H), 7.39 (ddd, 1H, ^{3}J = 8.0 Hz, ^{4}J = 2.0 Hz, ^{5}J = 1.1 Hz, 6´-H), 7.60 (tt, 1H. ^{3}J = 8.0 Hz, ^{4}J = 2.0 Hz, 5´-H), 8.55–8.58 (m, 1H, 3´-H). – ^{13}C-NMR (125 MHz, $CDCl_3$): δ = 50.8 (C1), 51.4 (C4), 72.9 (C7), 119.9 (C6´), 121.6 (C4´), 136.2 (C5´), 141.8 (C3), 142.9 (C6), 143.2 (C5), 149.5 (C3´), 154.6 (C1´), 157.6 (C2).

(4-Methoxyphenyl)-3-methyl-norbornadiene (**5k**)

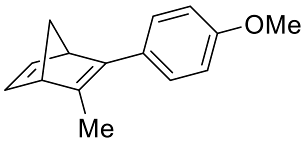

1. 4-Iodoanisole (**4k**, 55.7 mg, 238 µmol) and **2a** (50.0 mg, 216 µmol) were treated according to GP A. The ^{1}H NMR-spectroscopic analysis of the reaction mixture showed only the signals of the starting materials with no signs of new products.

2. 4-Iodoanisole (**4k**, 67.0 mg, 285 µmol) and **2a** (67.0 mg, 285 µmol) were treated according to GP B. The ^{1}H NMR-spectroscopic analysis of the reaction mixture showed only the signals of the starting materials with no signs of new products.

Bicyclo[2.2.1]heptadien-2-yl-boronic acid

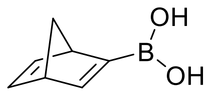

Attempt 1

A mixture suspension of *4,4,5,5-tetramethyl-2-(bicyclo-[2.2.1]heptadien-2-yl)-1,3,2-dioxaborolane* (**2a**, 100 mg, 459 µmol) and $NaIO_4$ (295 mg, 1.38 mmol) in THF/water (4:1, *v*:*v*, 4.0 mL) was stirred for 30 min at rt. An aqueous solution of HCl (1 M, 321 µmol, 321 µL) was added to the suspension, which immediately turned black. The TLC analysis showed that the substrate decomposed.

Attempt 2

A suspension of *potassium bicyclo[2.2.1]heptadien-2-yl-trifluoroborate* (**3**, 80.0 mg, 404 µmol) and LiOH (33.9 mg, 1.41 mmol) in water (2.0 mL) and MeCN (4.0 mL) was stirred for 20 h at rt. An aqueous solution of NH_4Cl (3.5 mL) was added followed by the addition of aq. HCl (1 M, 1 mL), which resulted in a black solution. The TLC analysis showed that the substrate decomposed.

1.5 Photoreactions

Starting from a stock solution in $CHCl_3$, a solution of **5b** in MeCN (c = 20 µM) was prepared. The experiments were performed with a sample volume of 2000 µL. An absorption spectrum of **5b** was recorded in the range of 200 nm to 370 nm. Subsequently, the sample was irradiated at λ_{ex} = 315 nm. The photoreaction was monitored photometrically until no further changes in the spectra occurred. The thermal backconversion of **6b** into **5b** was investigated at 60 °C, monitored by the increase of the norbornadiene absorbance at 301 nm. The spectra were processed with the software Origin with the implemented smoothing function “adjacent-averagin” with a factor of 10 [4].

2 NMR spectra

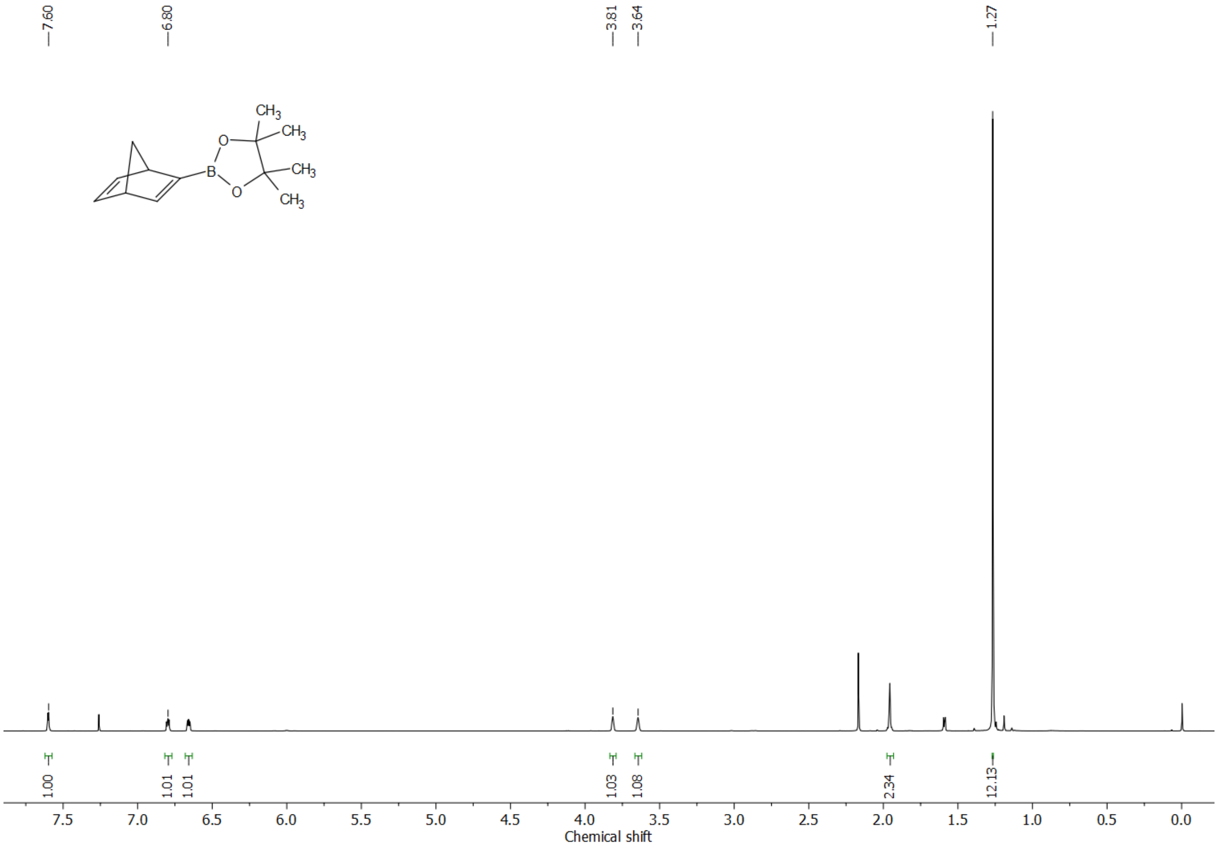

Figure S1: ^1H NMR spectrum of **2a** (500 MHz) in $CDCl_3$.

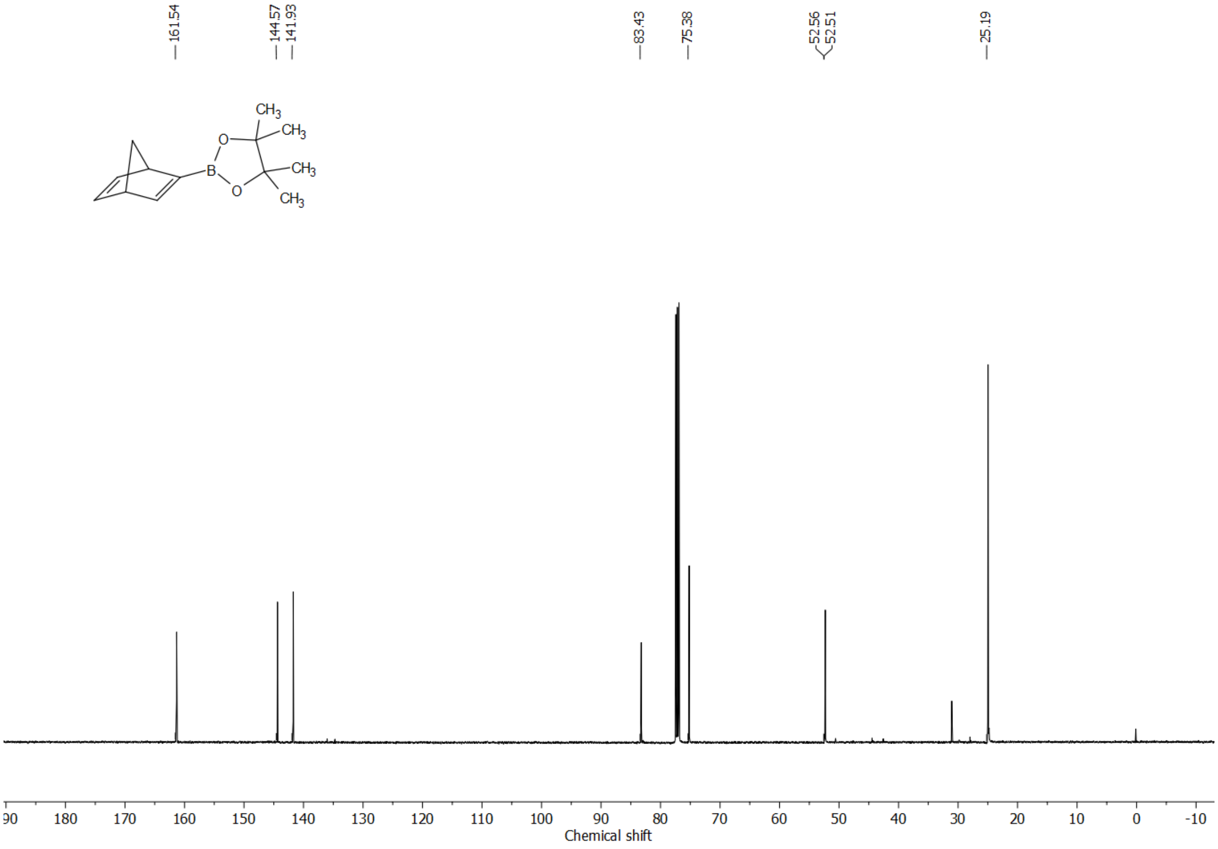

Figure S2: ^{13}C NMR spectrum of **2a** (125 MHz) in $CDCl_3$.

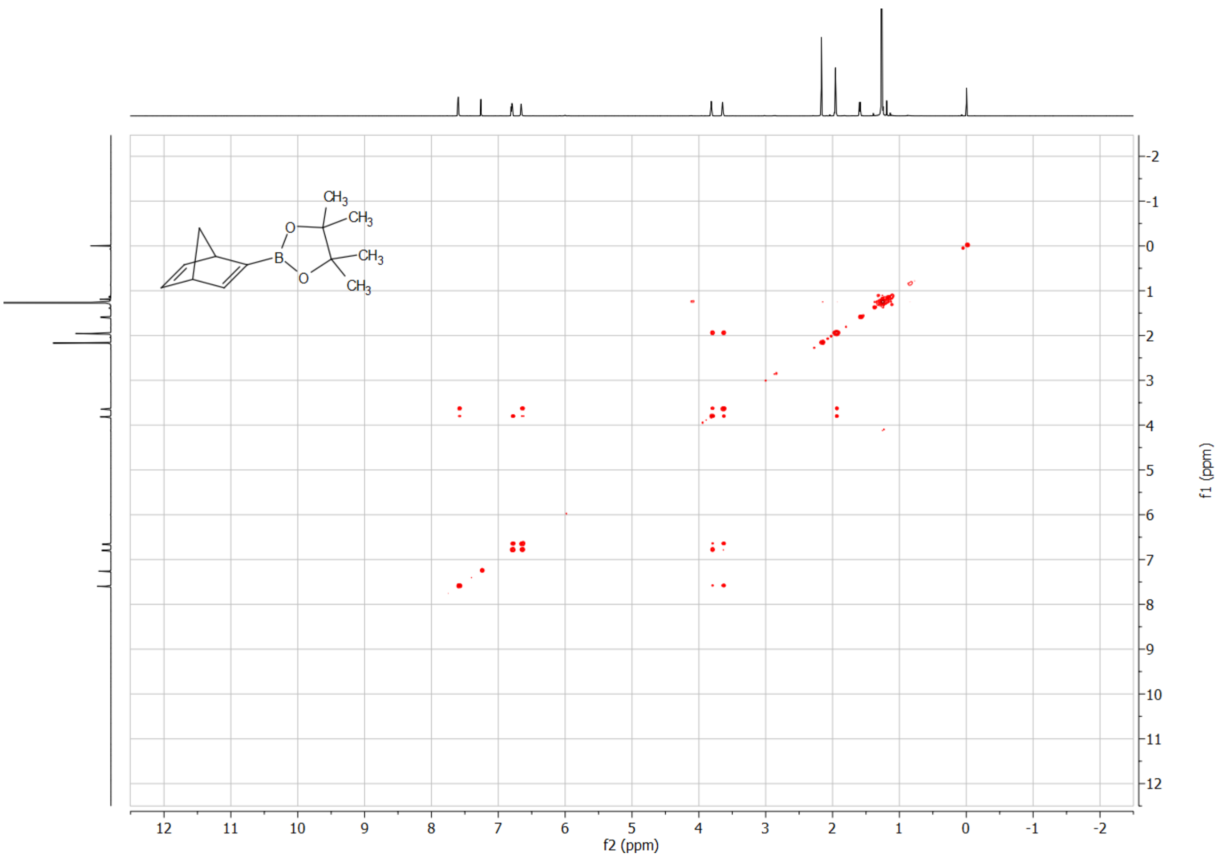

Figure S3: H,H-COSY-NMR spectrum of **2a** (500 MHz, 500 MHz) in $CDCl_3$.

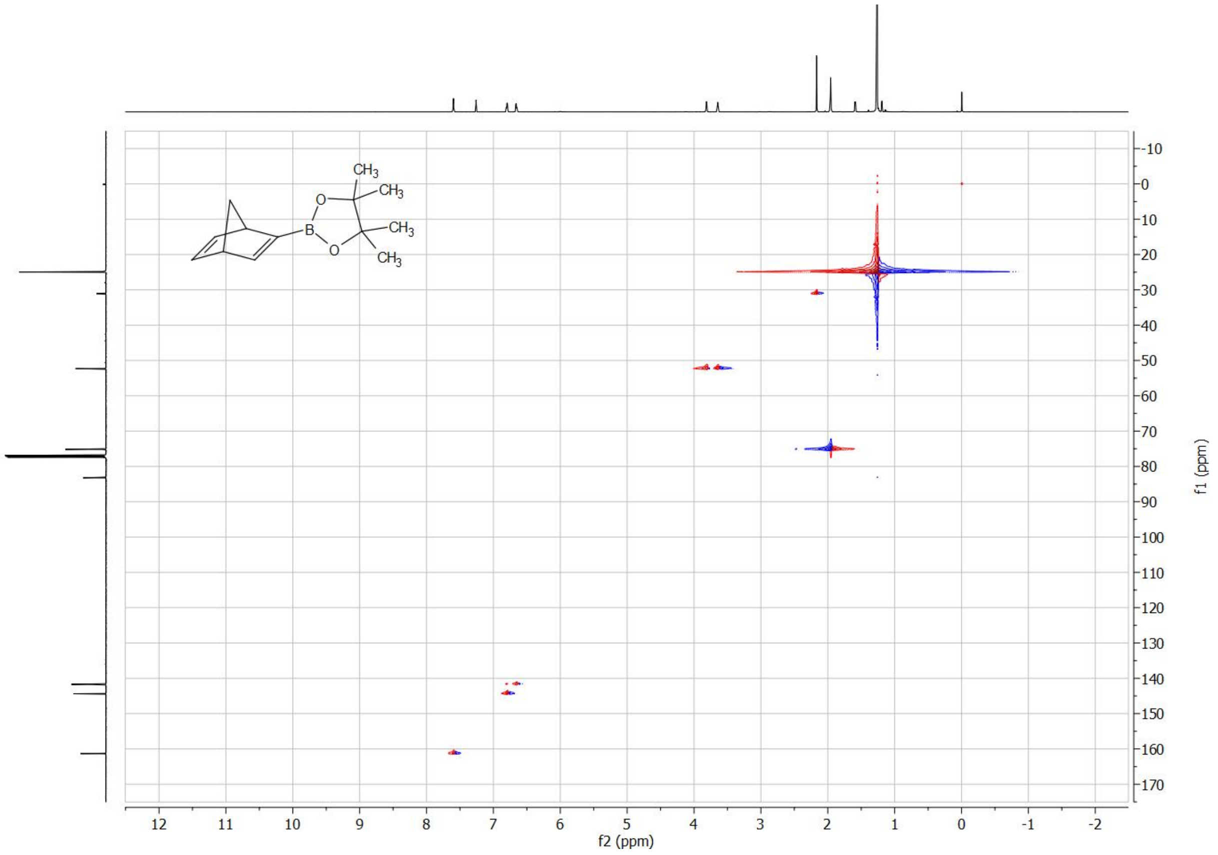

Figure S4: HSQC-NMR spectrum of **2a** (500 MHz, 125 MHz) in $CDCl_3$.

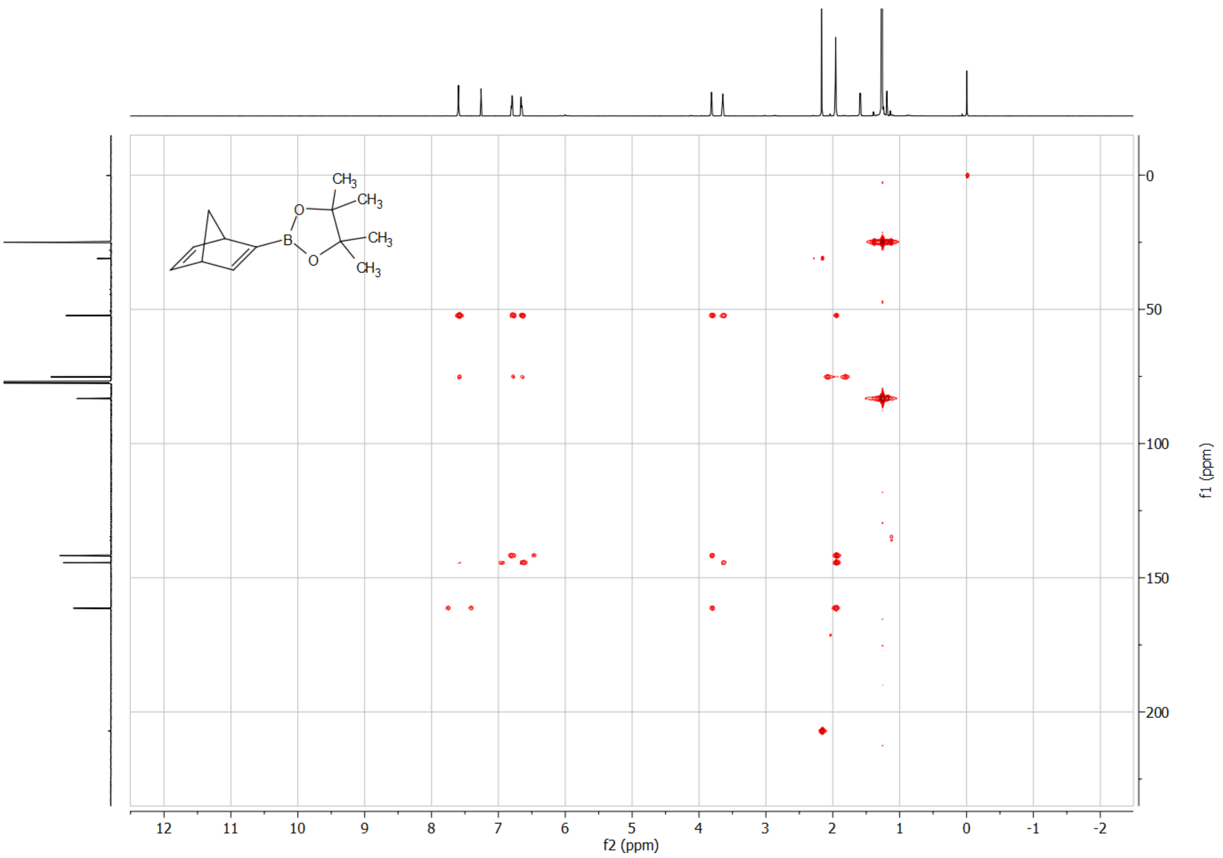

Figure S5: HMBC-NMR spectrum of **2a** (500 MHz, 125 MHz) in $CDCl_3$.

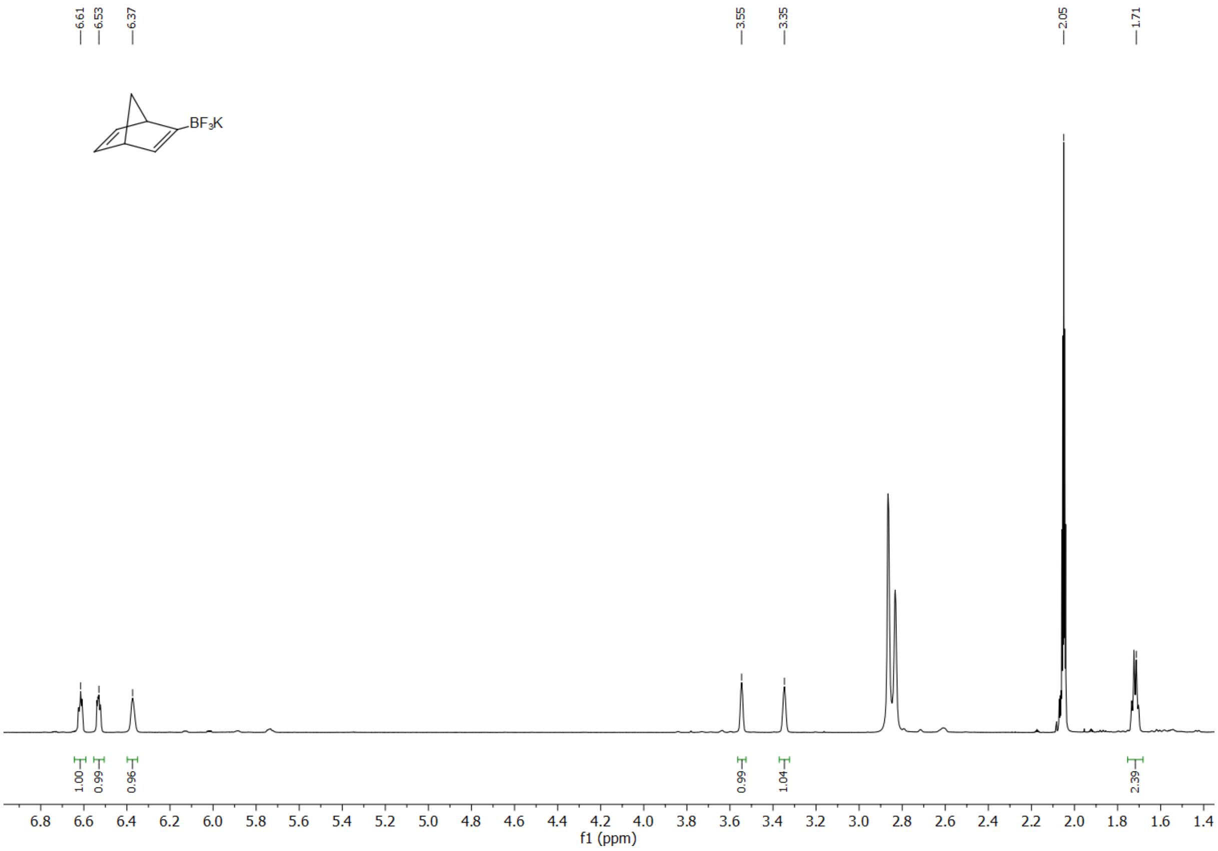

Figure S6: ^{1}H NMR spectrum of **3** (500 MHz) in $CDCl_3$.

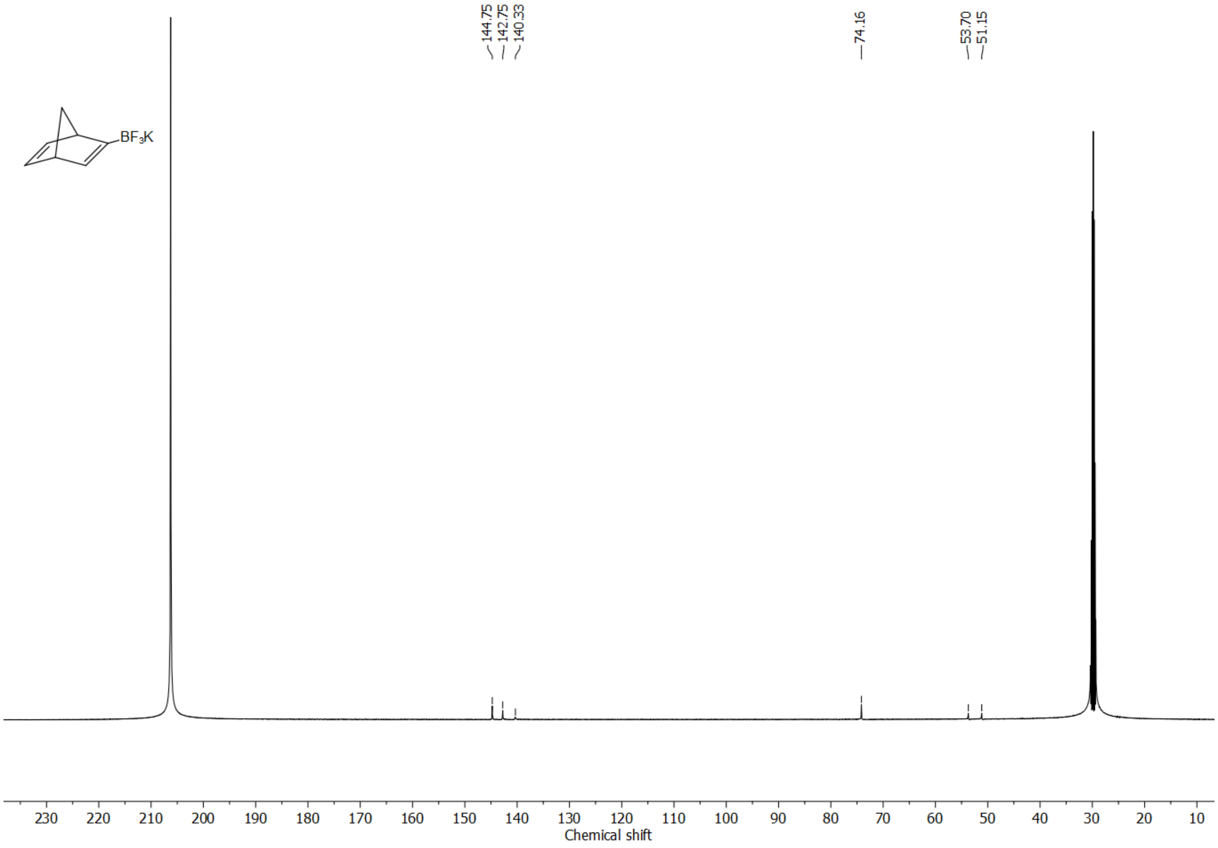

Figure S7: ^{13}C NMR spectrum of **3** (125 MHz) in $CDCl_3$.

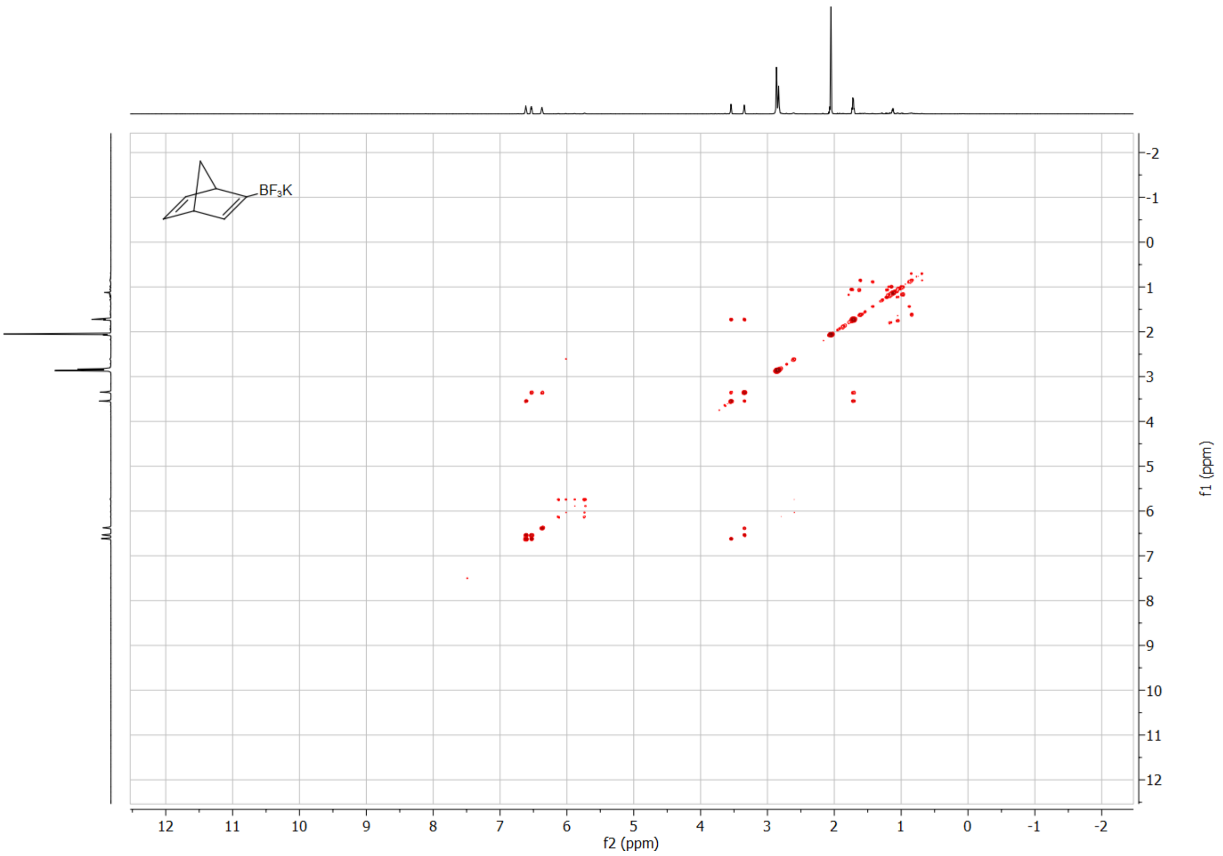

Figure S8: H,H-COSY-NMR spectrum of **3** (500 MHz, 500 MHz) in $CDCl_3$.

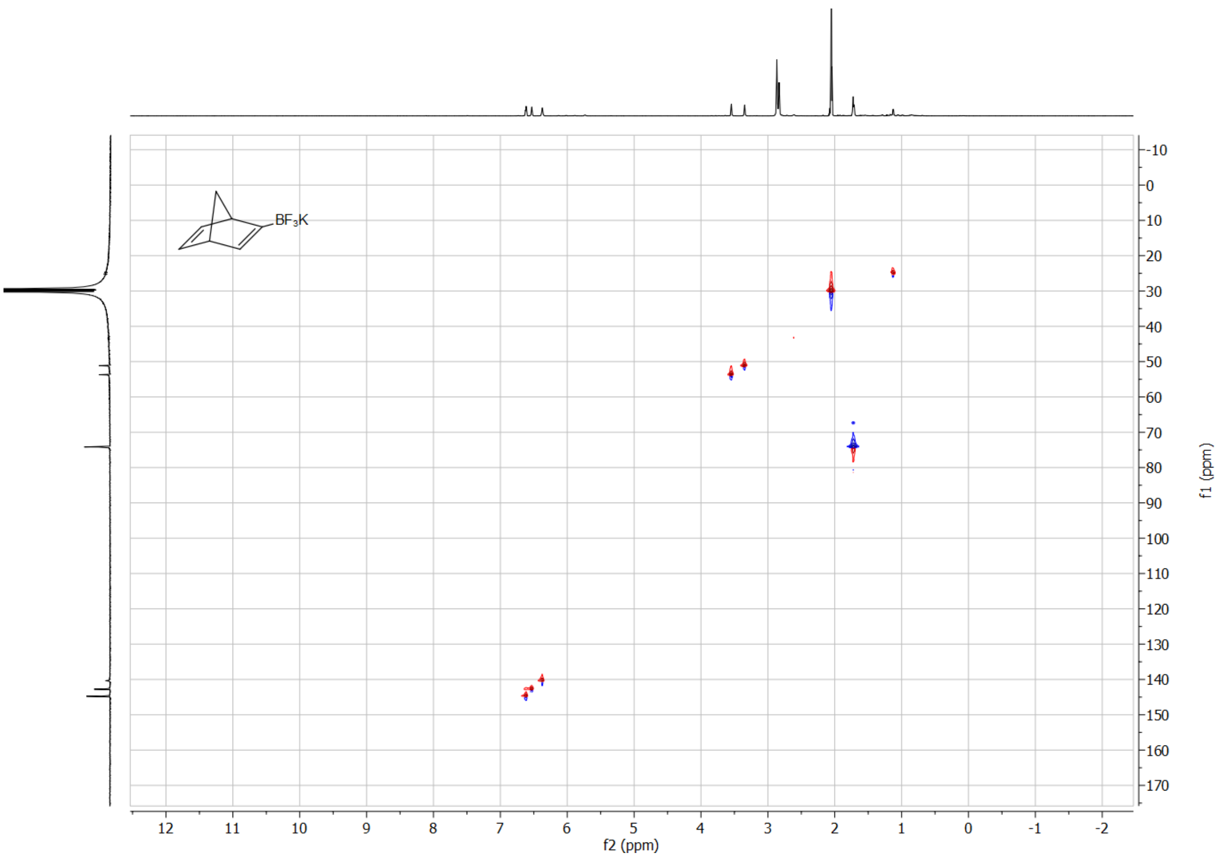

Figure S9: HSQC-NMR spectrum of **3** (500 MHz, 125 MHz) in $CDCl_3$.

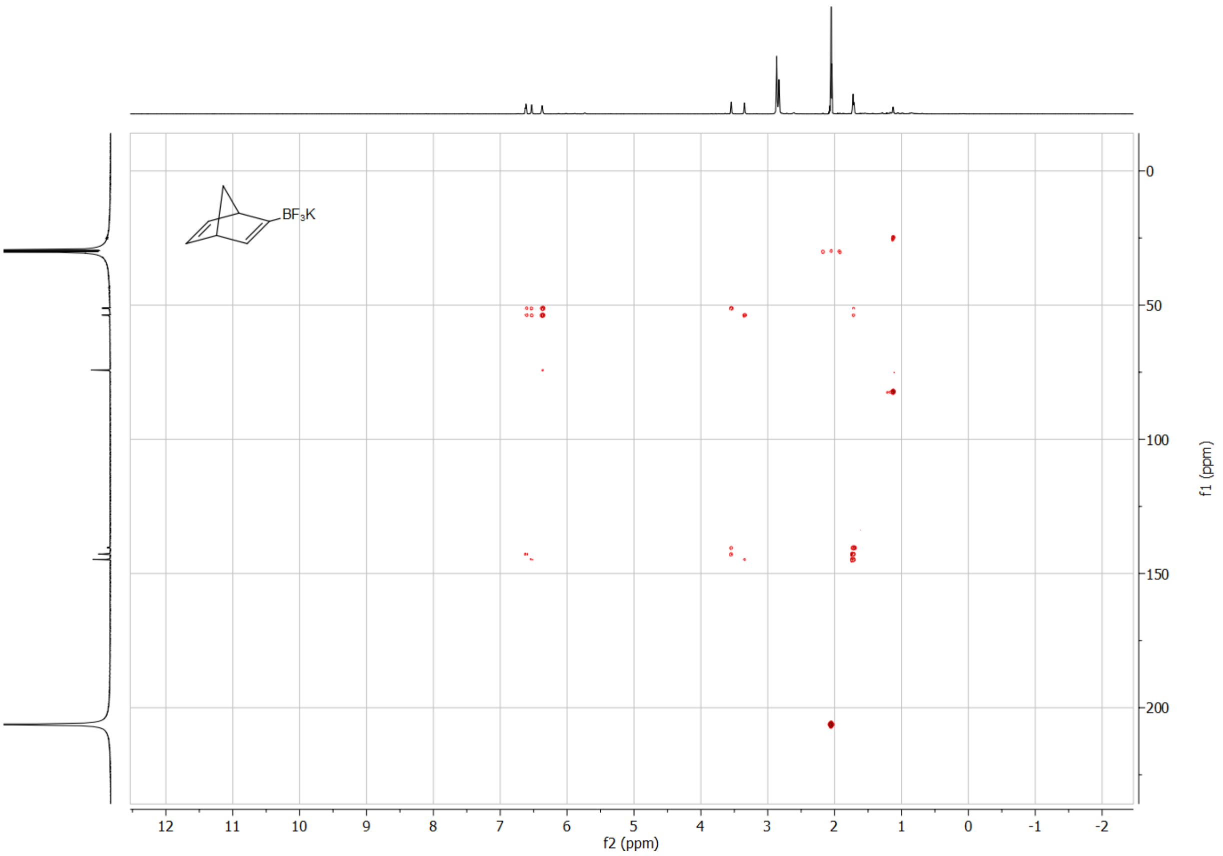

Figure S10: HMBC-NMR spectrum of **3** (500 MHz, 125 MHz) in $CDCl_3$.

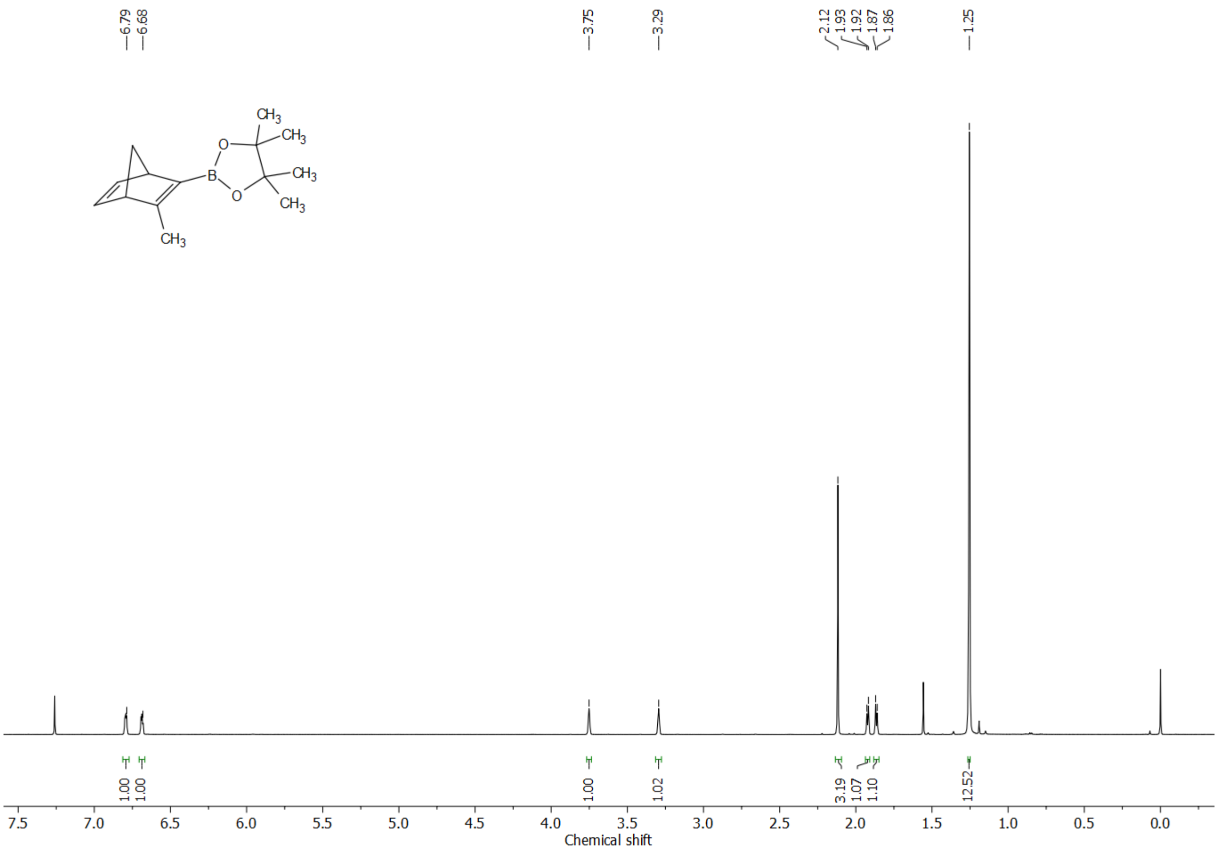

Figure S11: ^{1}H NMR spectrum of **2b** (600 MHz) in $CDCl_3$.

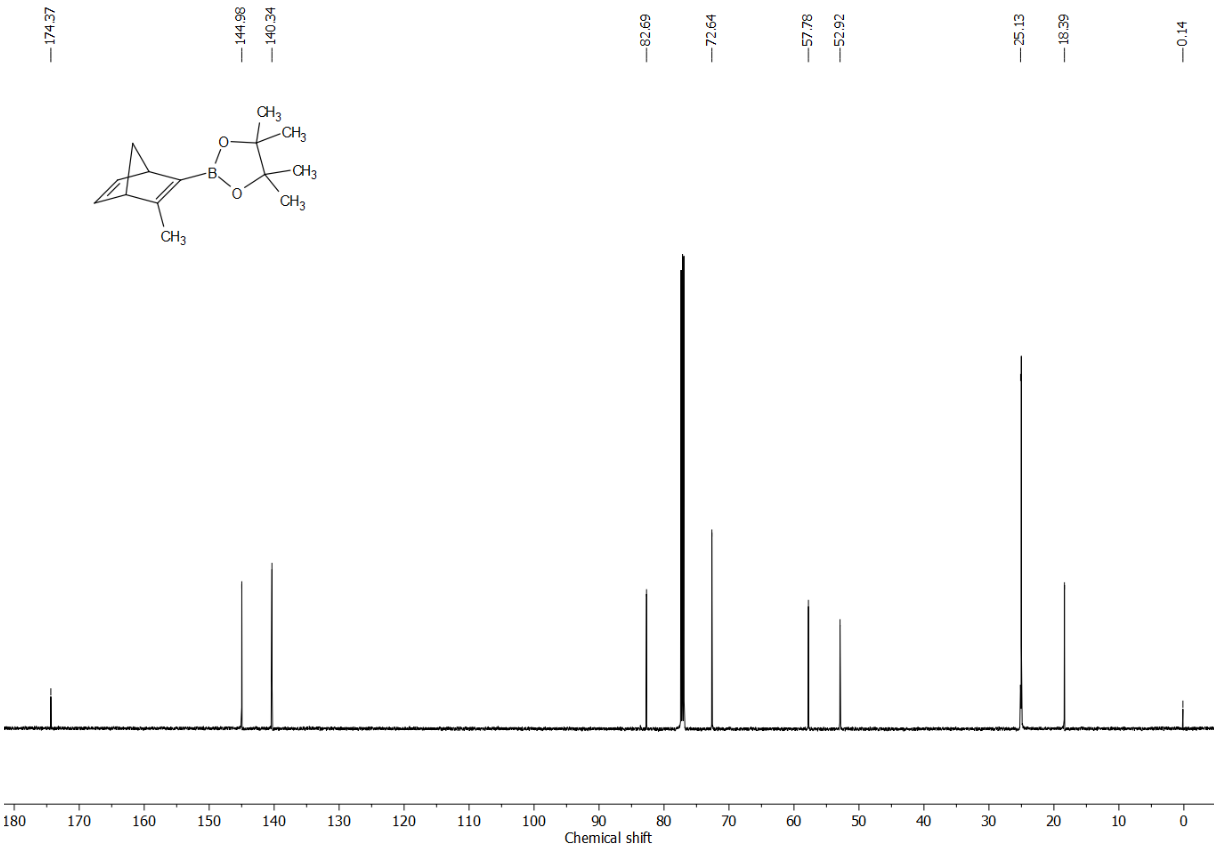

Figure S12: ^{13}C NMR spectrum of **2b** (150 MHz) in $CDCl_3$.

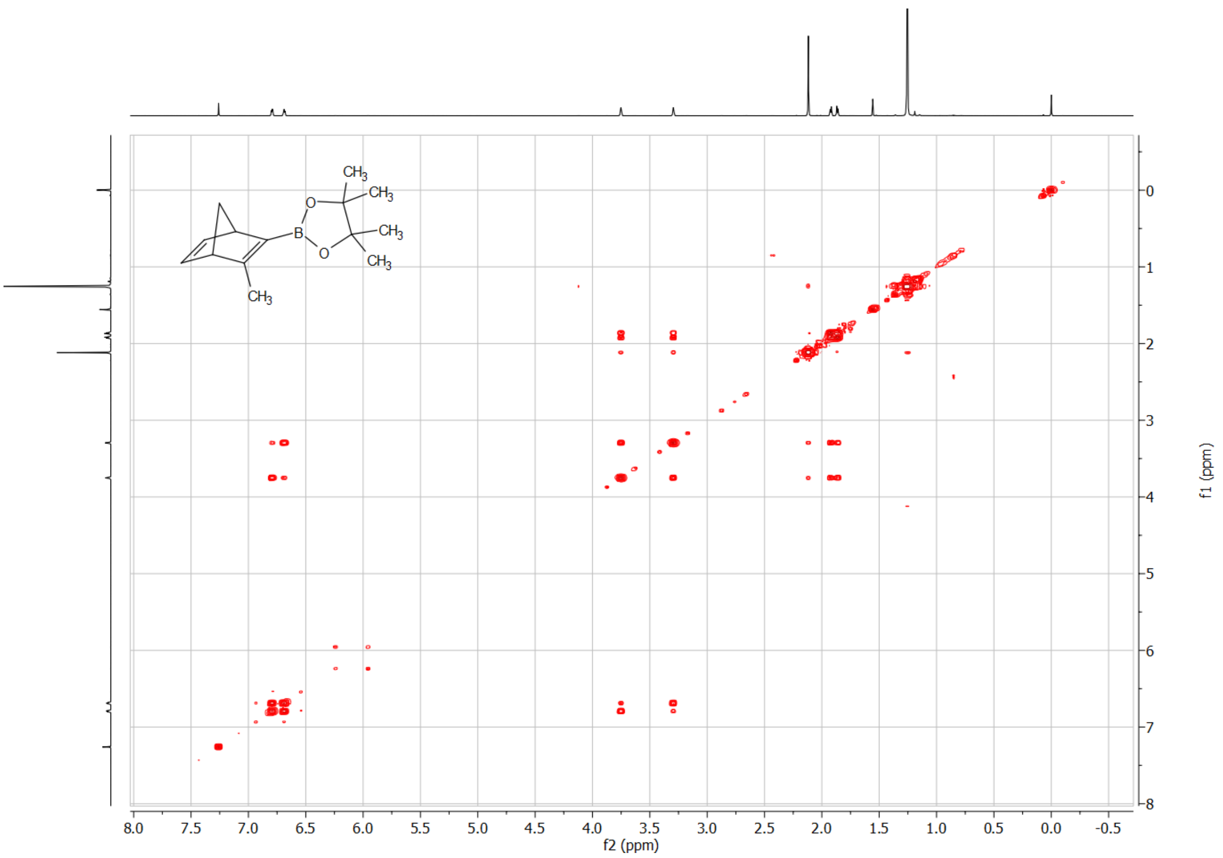

Figure S13: H,H-COSY-NMR spectrum of **2b** (600 MHz, 600 MHz) in $CDCl_3$.

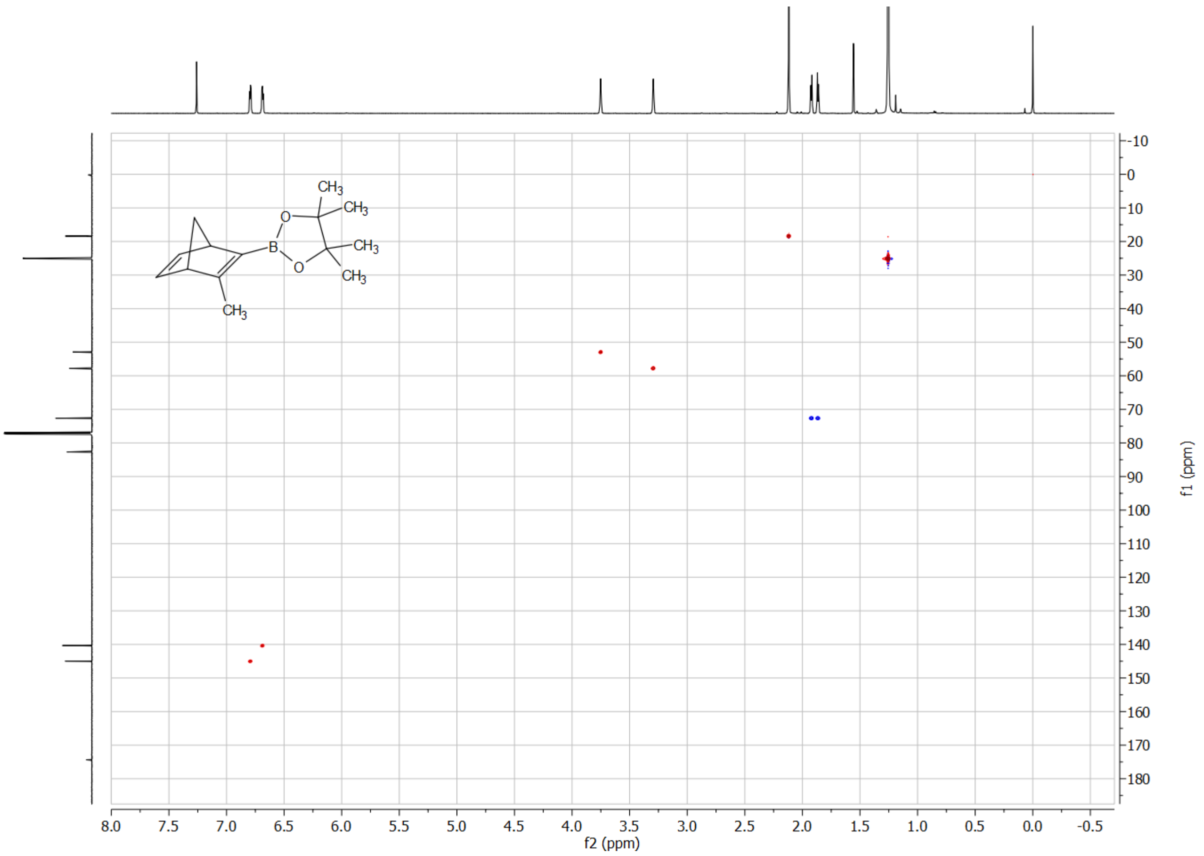

Figure S14: HSQC-NMR spectrum of **2b** (500 MHz, 150 MHz) in $CDCl_3$.

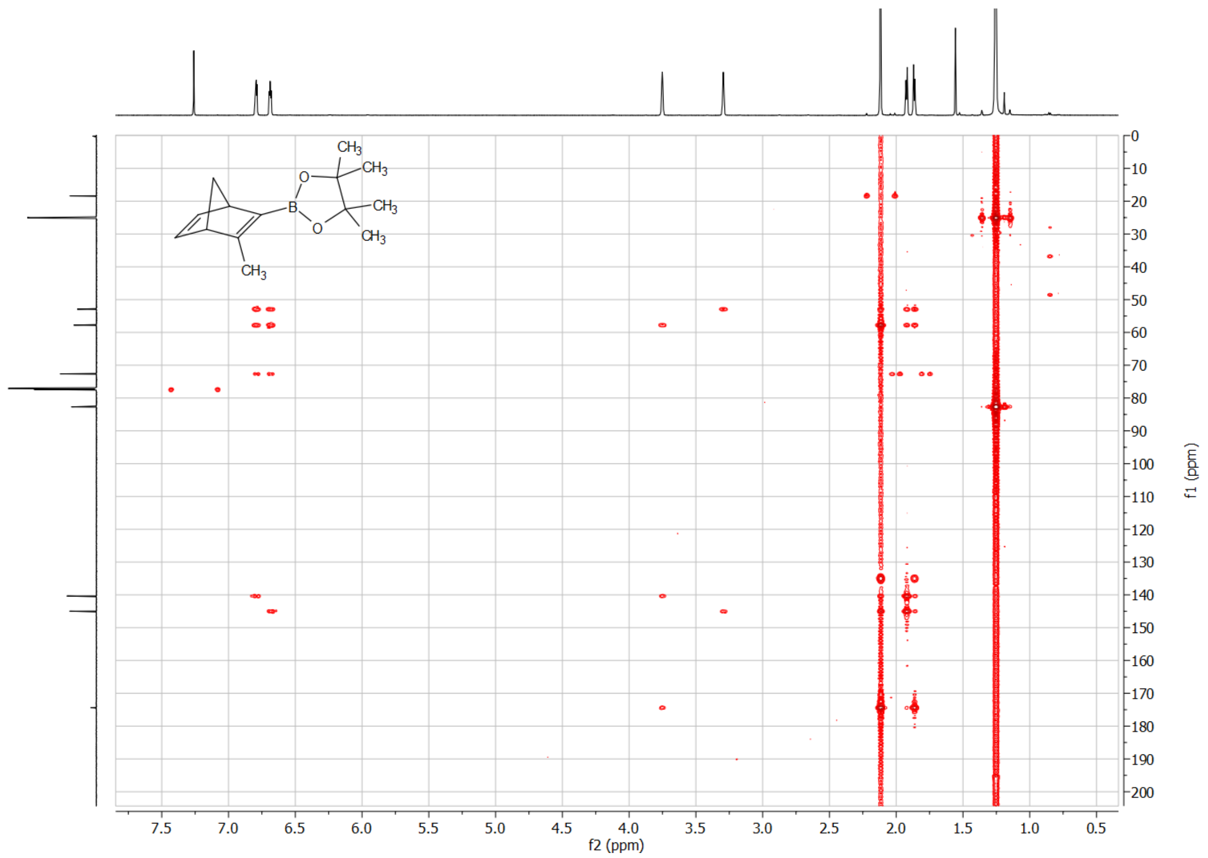

Figure S15: HMBC-NMR spectrum of **2b** (600 MHz, 150 MHz) in $CDCl_3$.

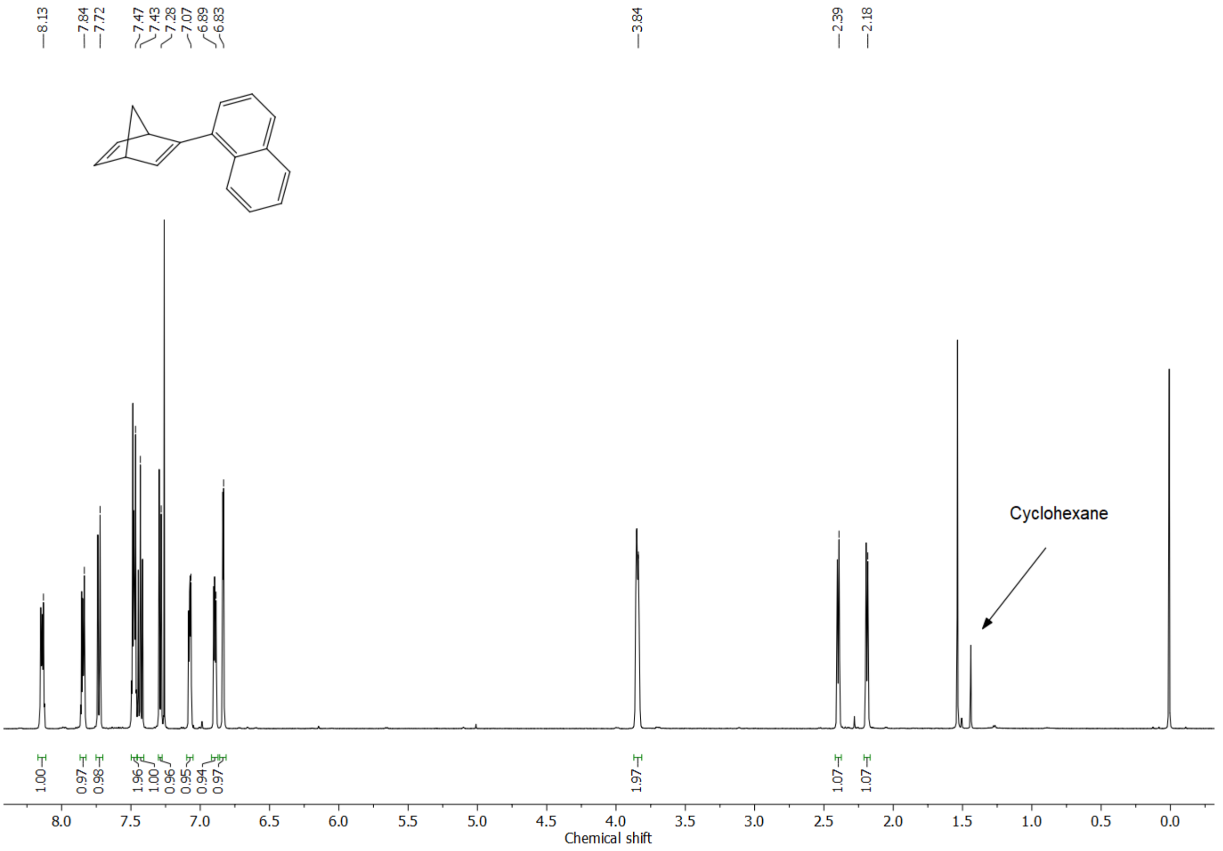

Figure S16: ^{1}H NMR spectrum of **5b** (500 MHz) in $CDCl_3$.

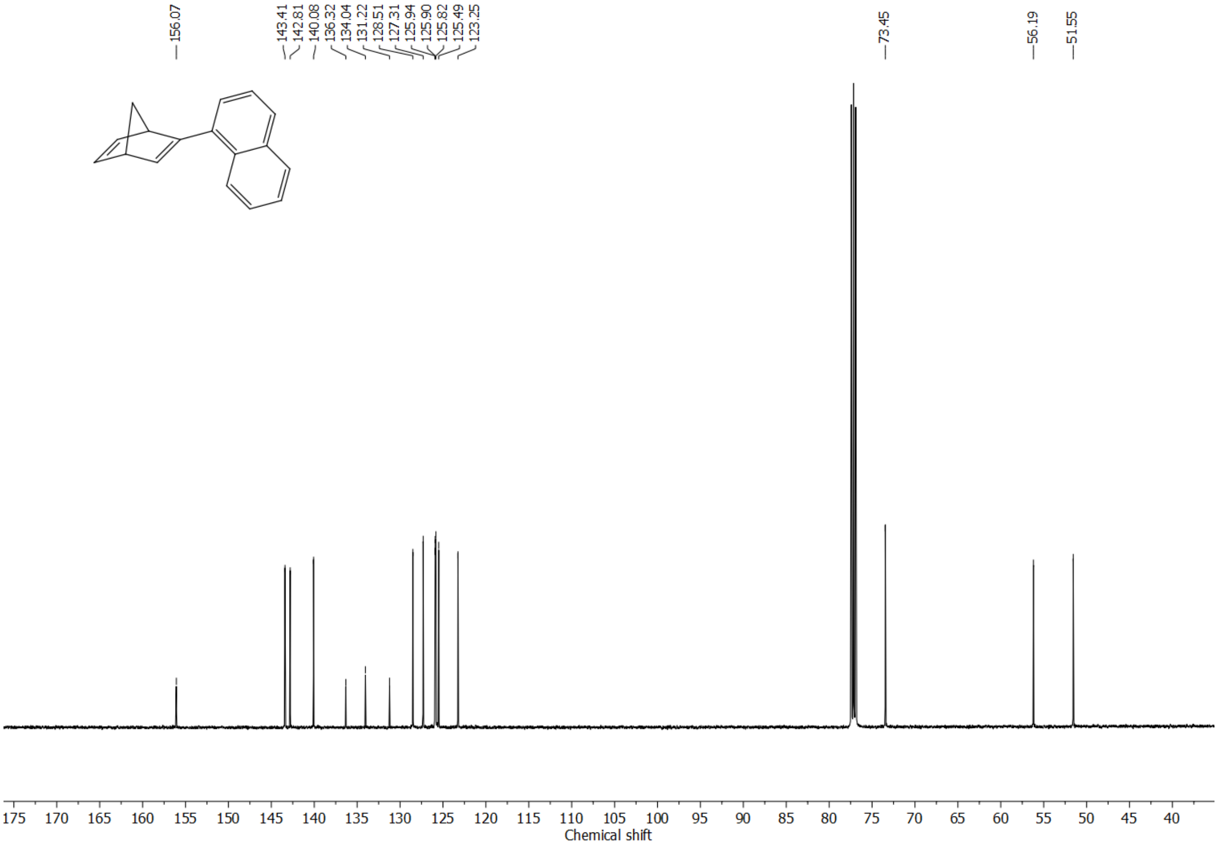

Figure S17: ^{13}C NMR spectrum of **5b** (125 MHz) in $CDCl_3$.

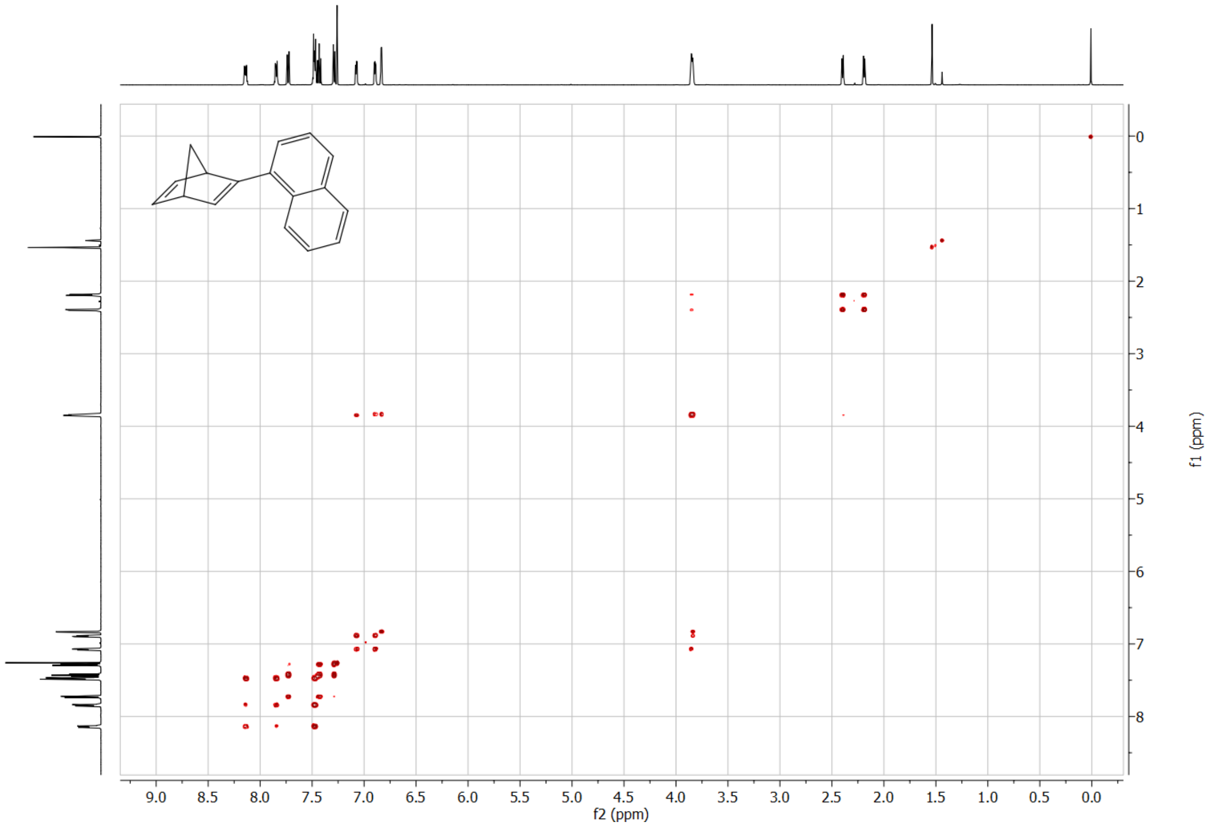

Figure S18: H,H-COSY-NMR spectrum of **5b** (500 MHz, 500 MHz) in $CDCl_3$.

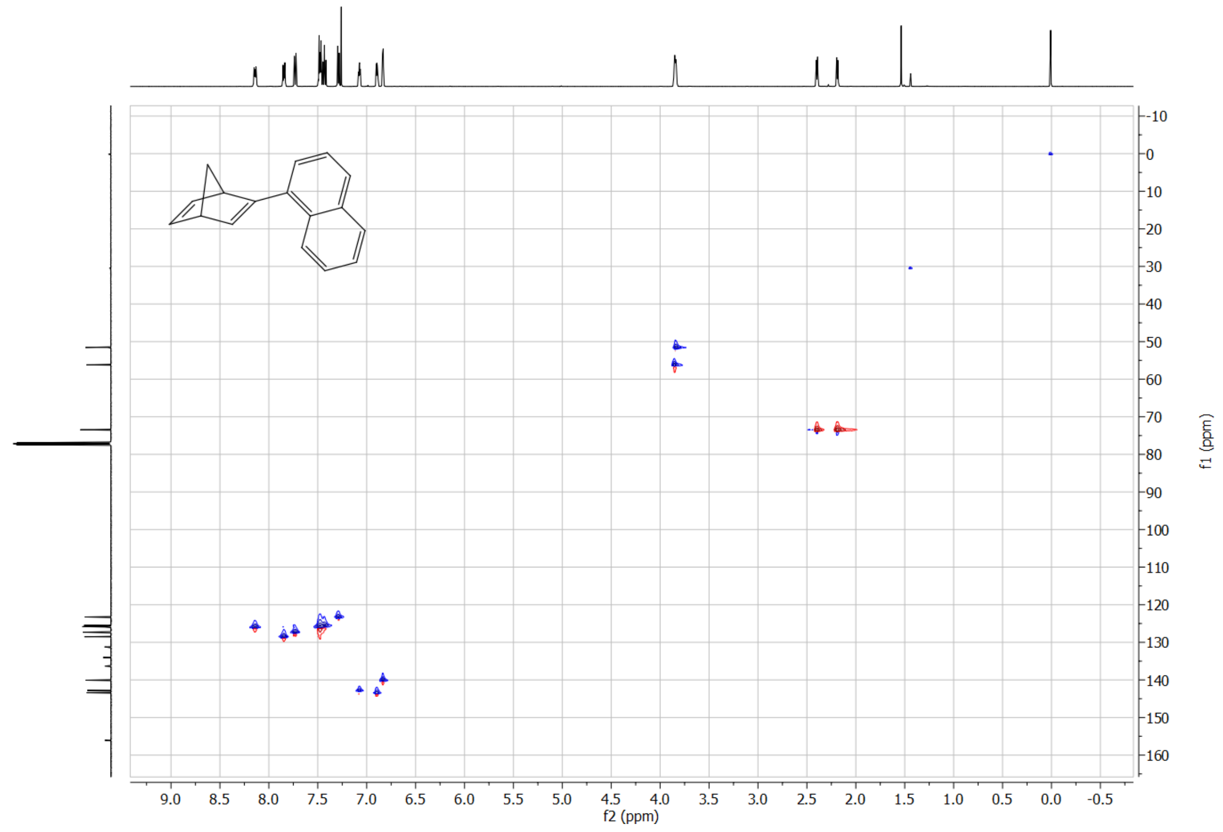

Figure S19: HSQC-NMR spectrum of **5b** (500 MHz, 125 MHz) in $CDCl_3$.

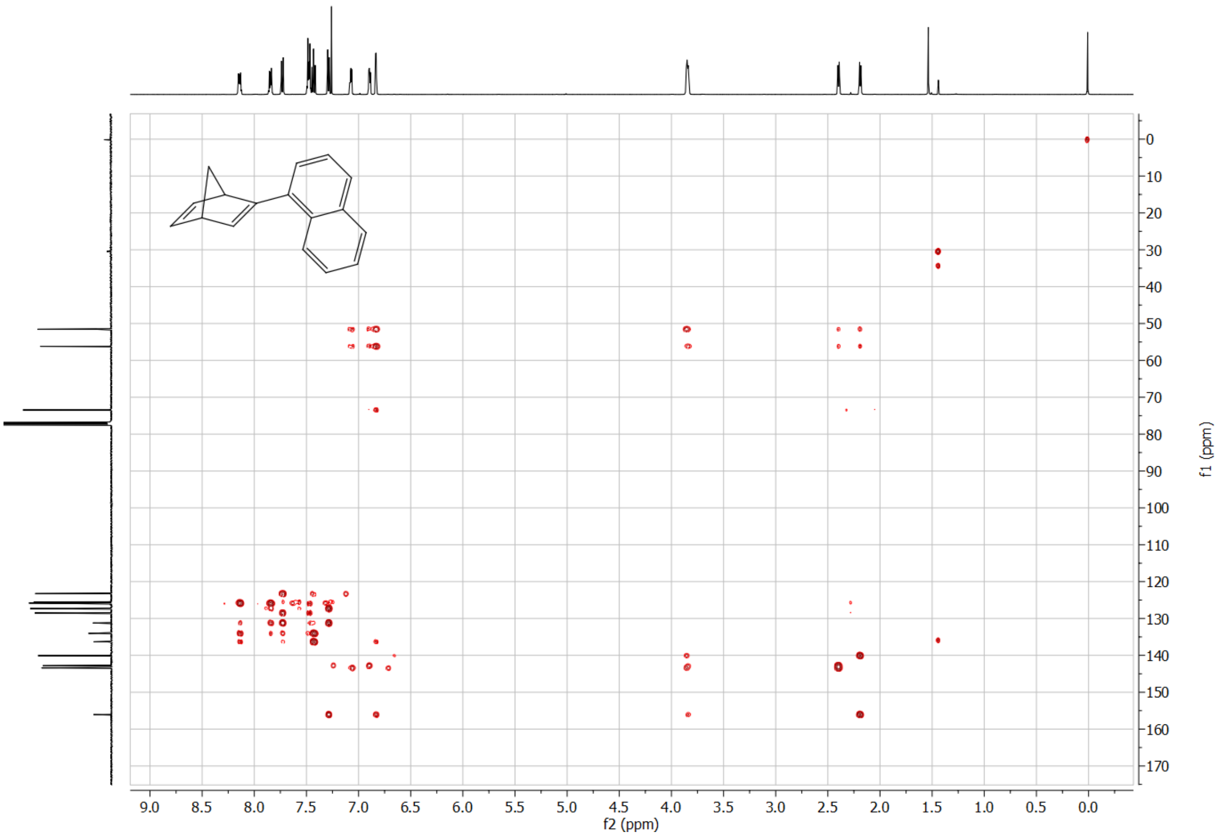

Figure S20: HMBC-NMR spectrum of **5b** (500 MHz, 125 MHz) in $CDCl_3$.

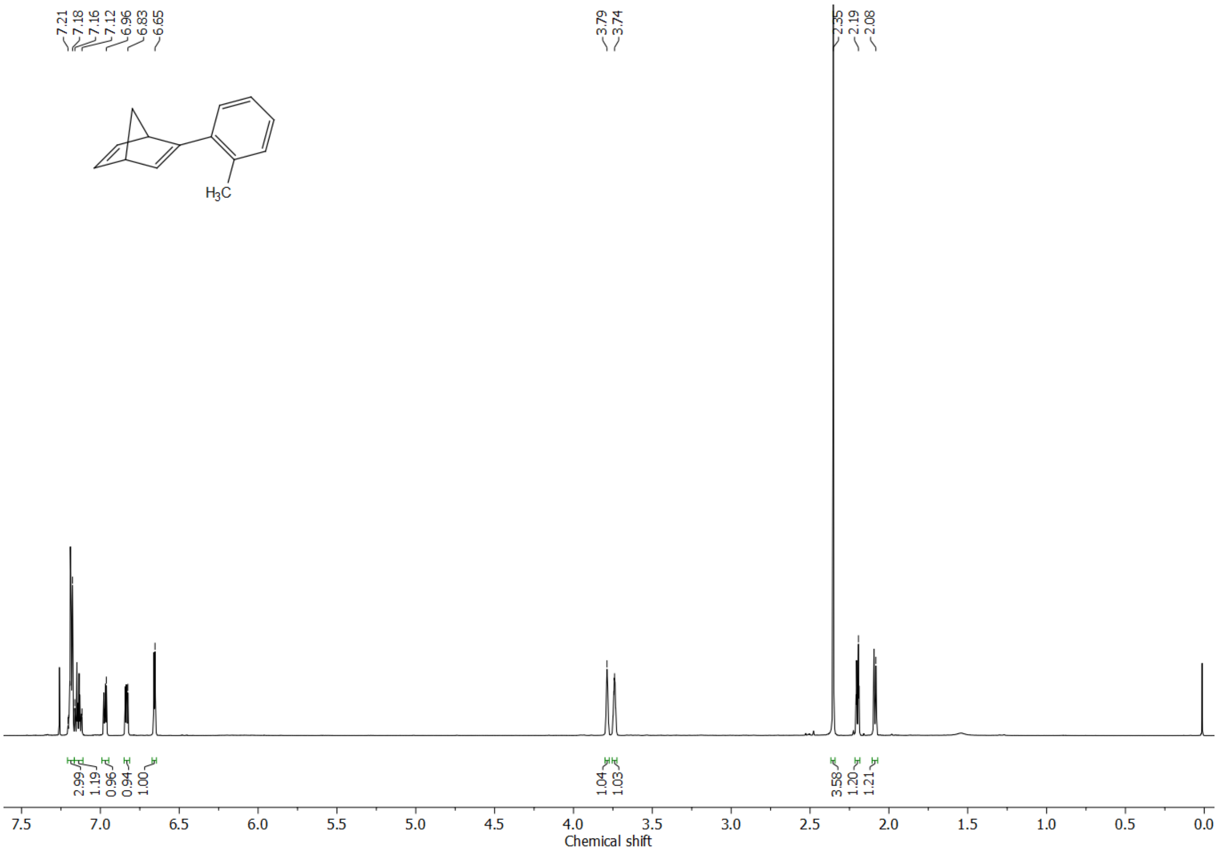

Figure S21: ^{1}H NMR spectrum of **5c** (500 MHz) in $CDCl_3$.

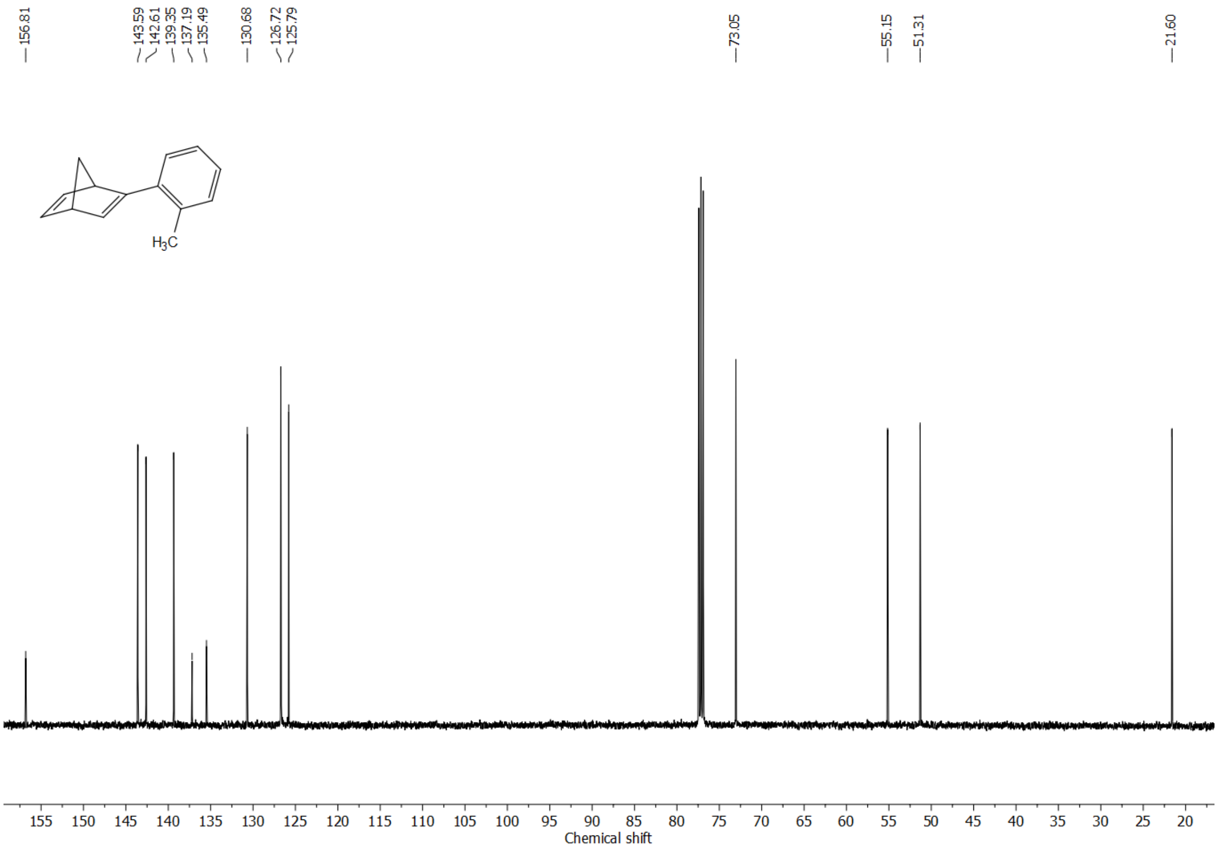

Figure S22: ^{13}C NMR spectrum of **5c** (125 MHz) in $CDCl_3$.

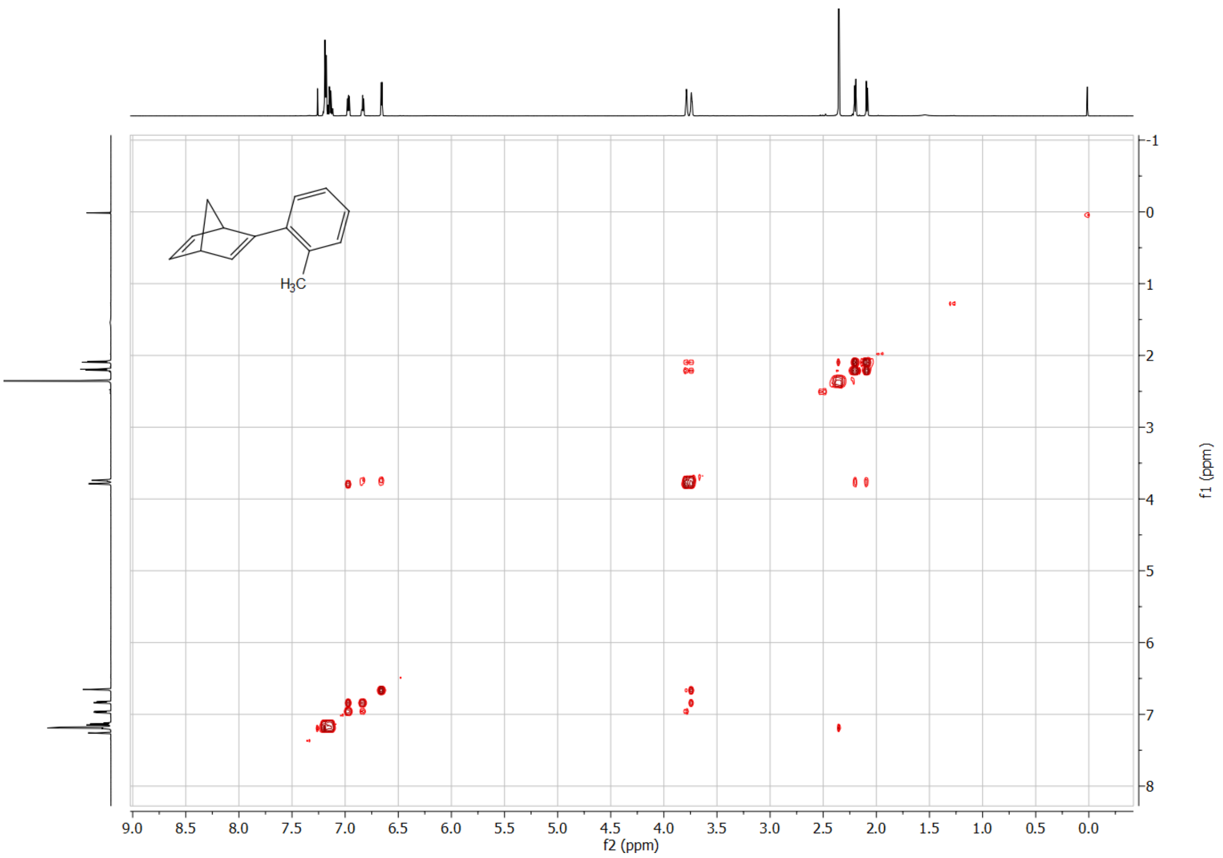

Figure S23: H,H-COSY-NMR spectrum of **5c** (500 MHz, 500 MHz) in $CDCl_3$.

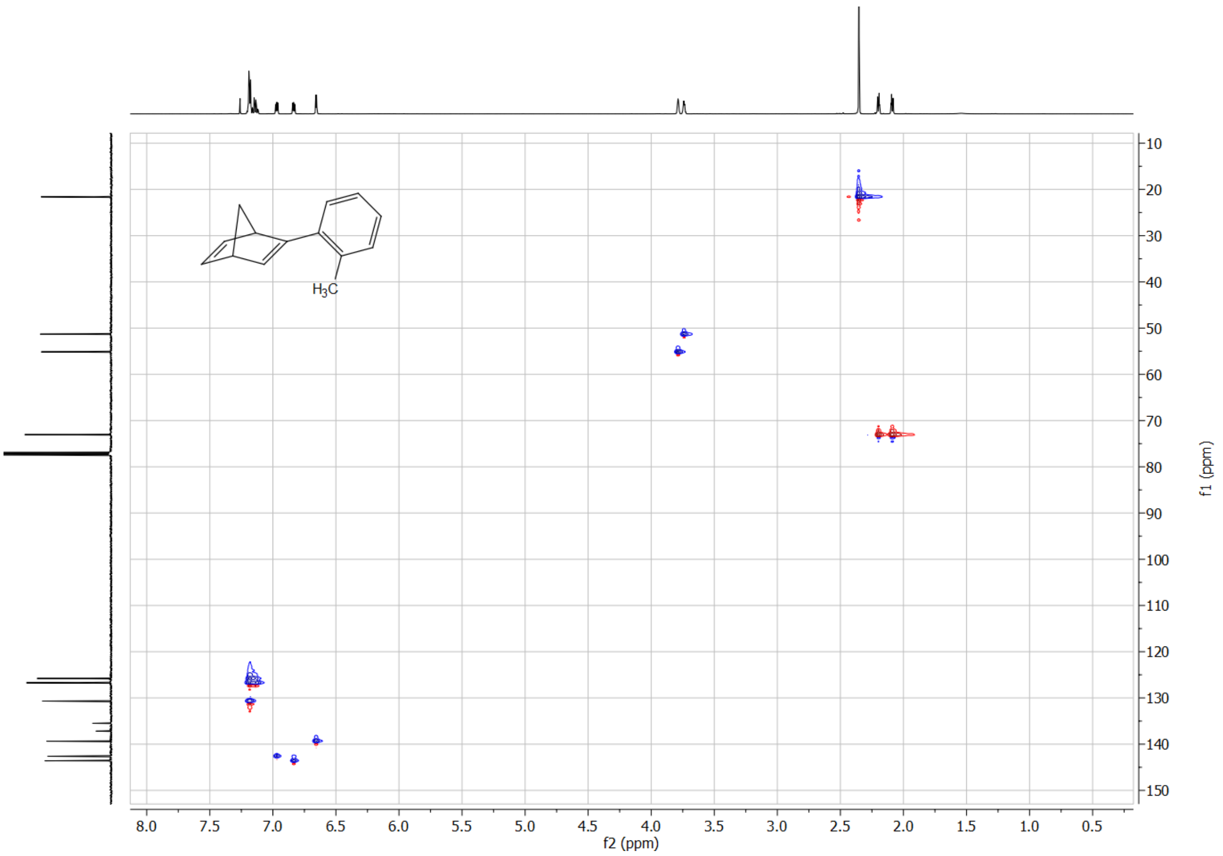

Figure S24: HSQC-NMR spectrum of **5c** (500 MHz, 125 MHz) in $CDCl_3$.

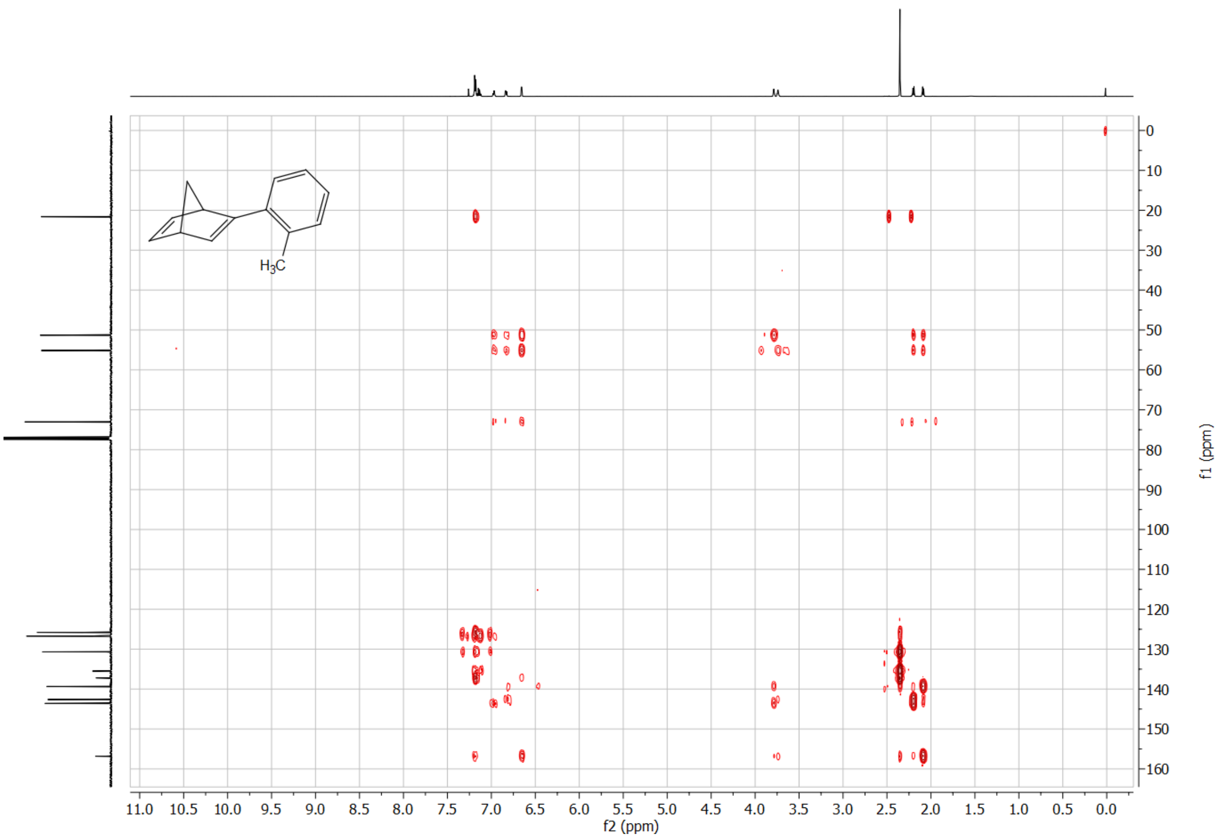

Figure S25: HMBC-NMR spectrum of **5c** (500 MHz, 125 MHz) in $CDCl_3$.

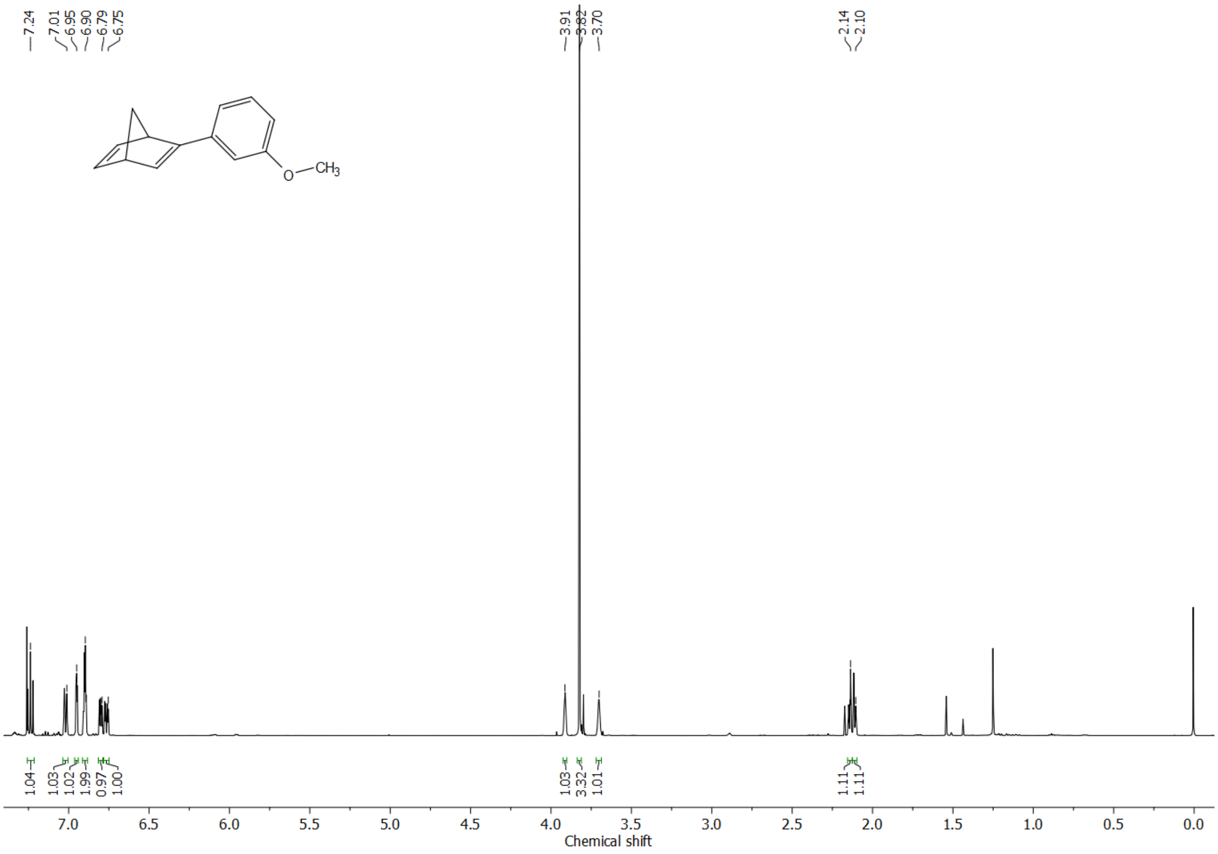

Figure S26: ^{1}H NMR spectrum of **5e** (500 MHz) in $CDCl_3$.

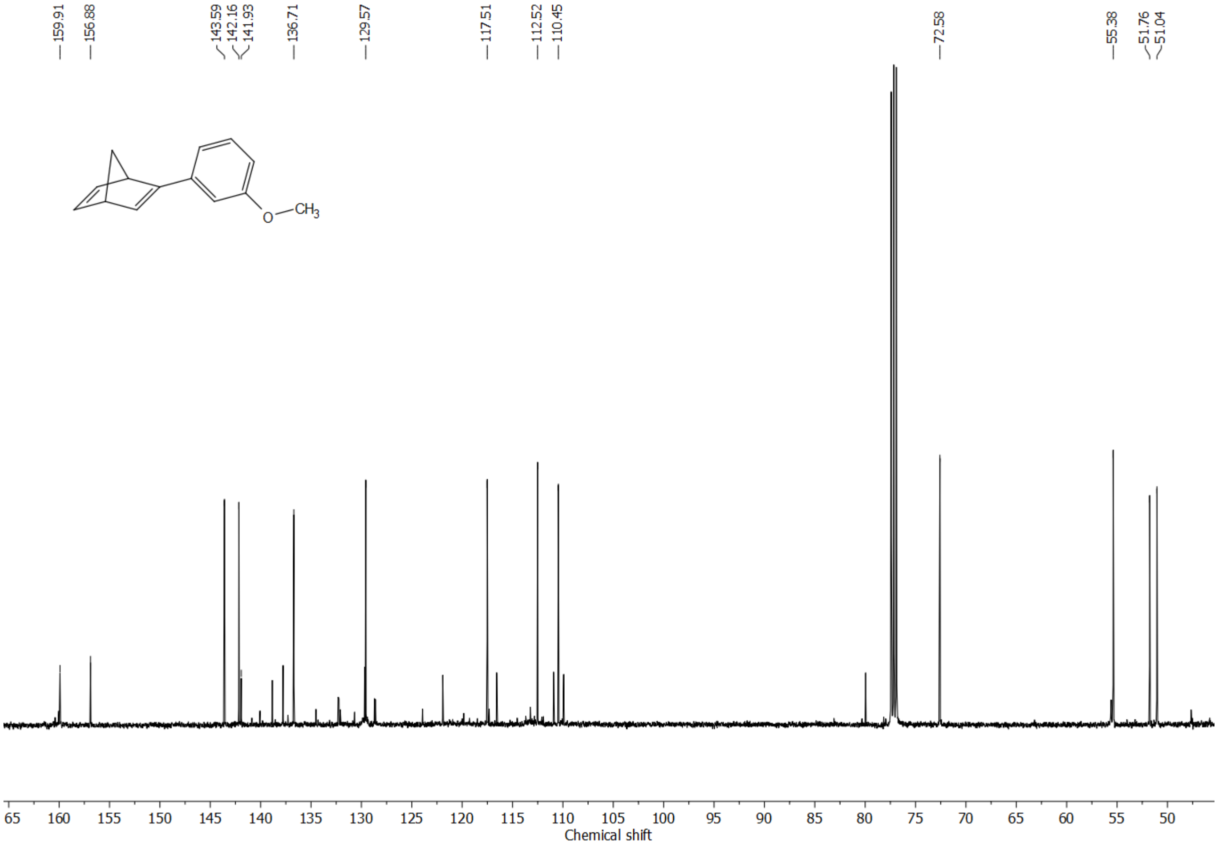

Figure S27: ^{13}C NMR spectrum of **5e** (125 MHz) in $CDCl_3$.

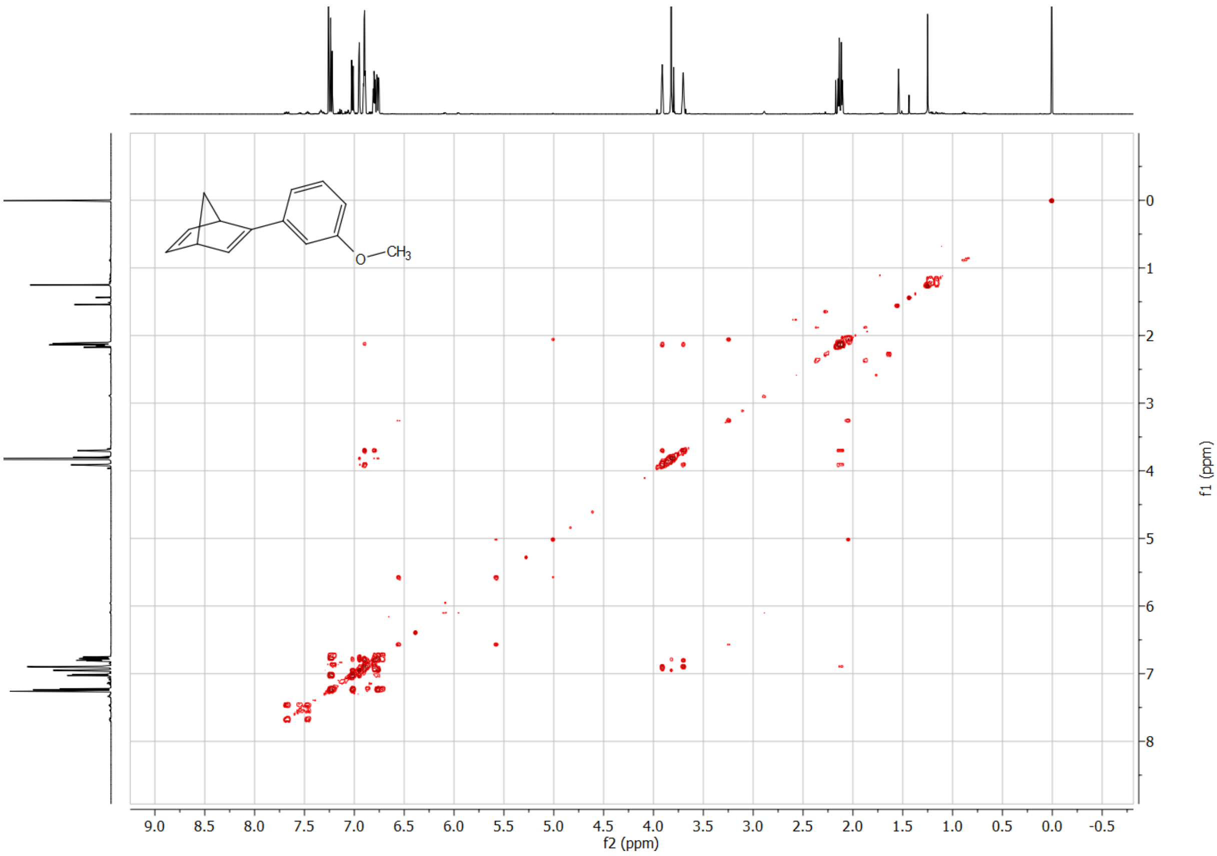

Figure S28: H,H-COSY-NMR spectrum of **5e** (500 MHz, 500 MHz) in $CDCl_3$.

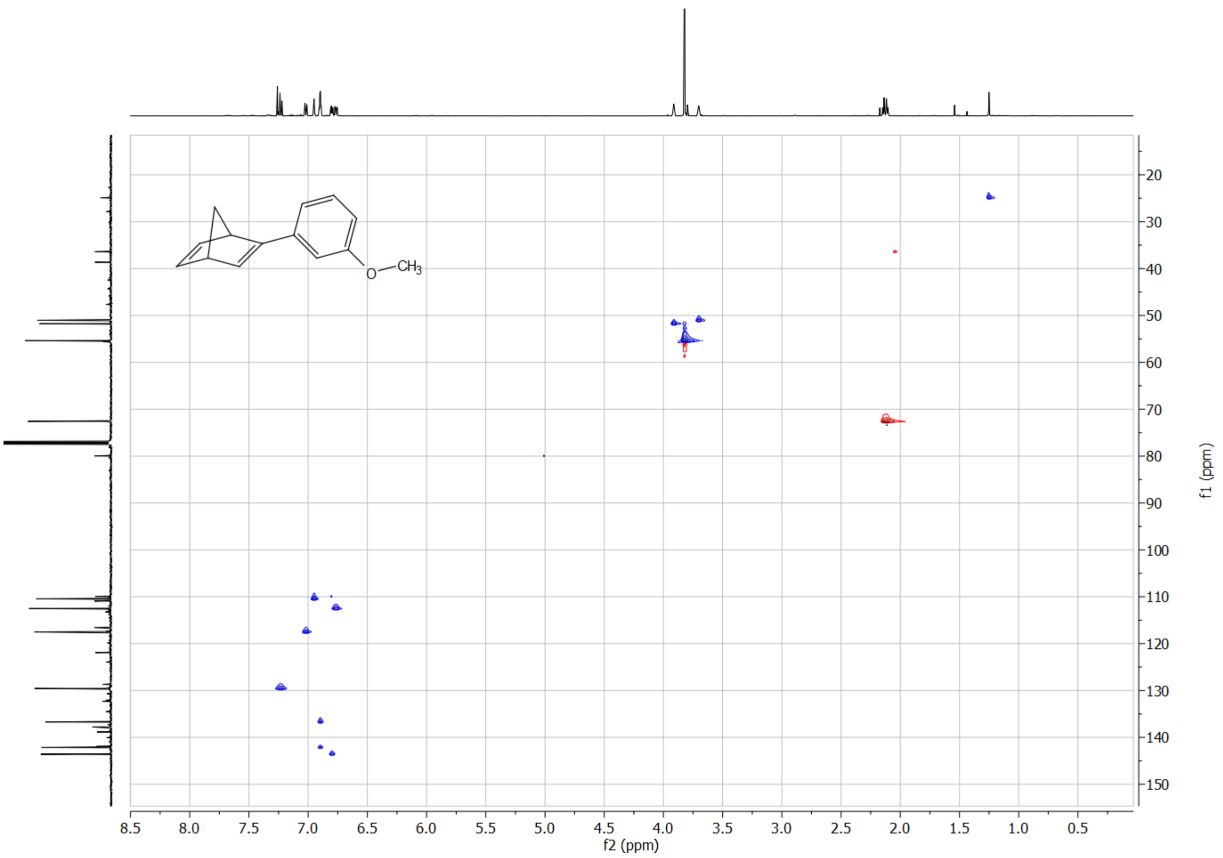

Figure S29: HSQC-NMR spectrum of **5e** (500 MHz, 125 MHz) in $CDCl_3$.

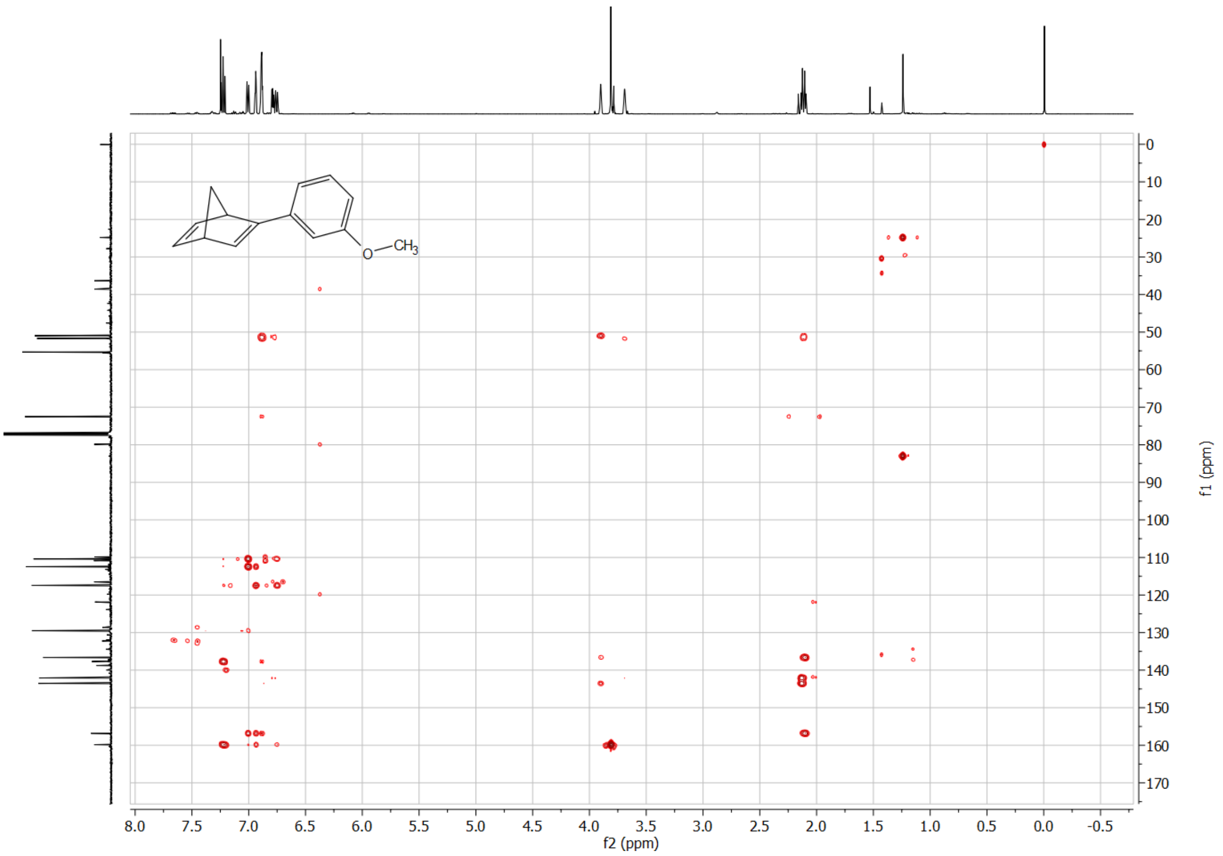

Figure S30: HMBC-NMR spectrum of **5e** (500 MHz, 125 MHz) in $CDCl_3$.

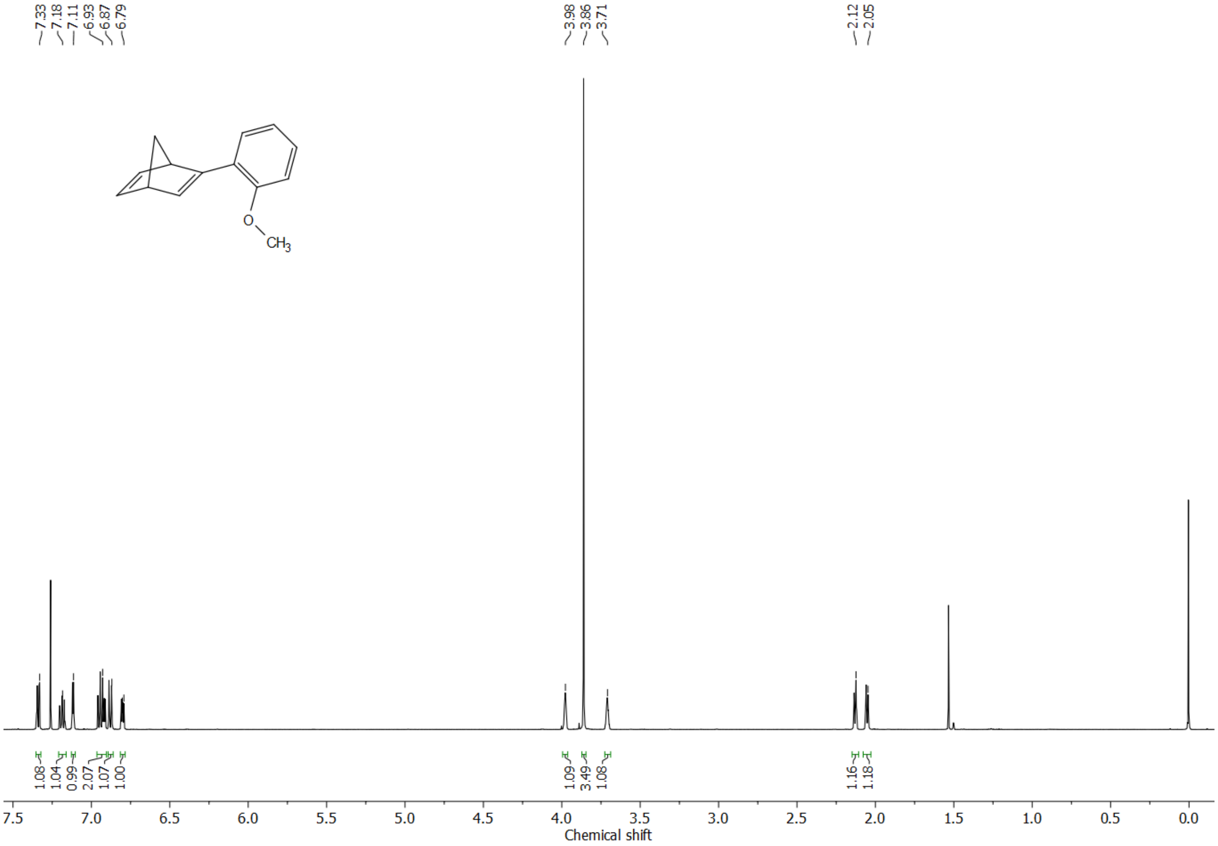

Figure S31: ^{1}H NMR spectrum of **5f** (500 MHz) in $CDCl_3$.

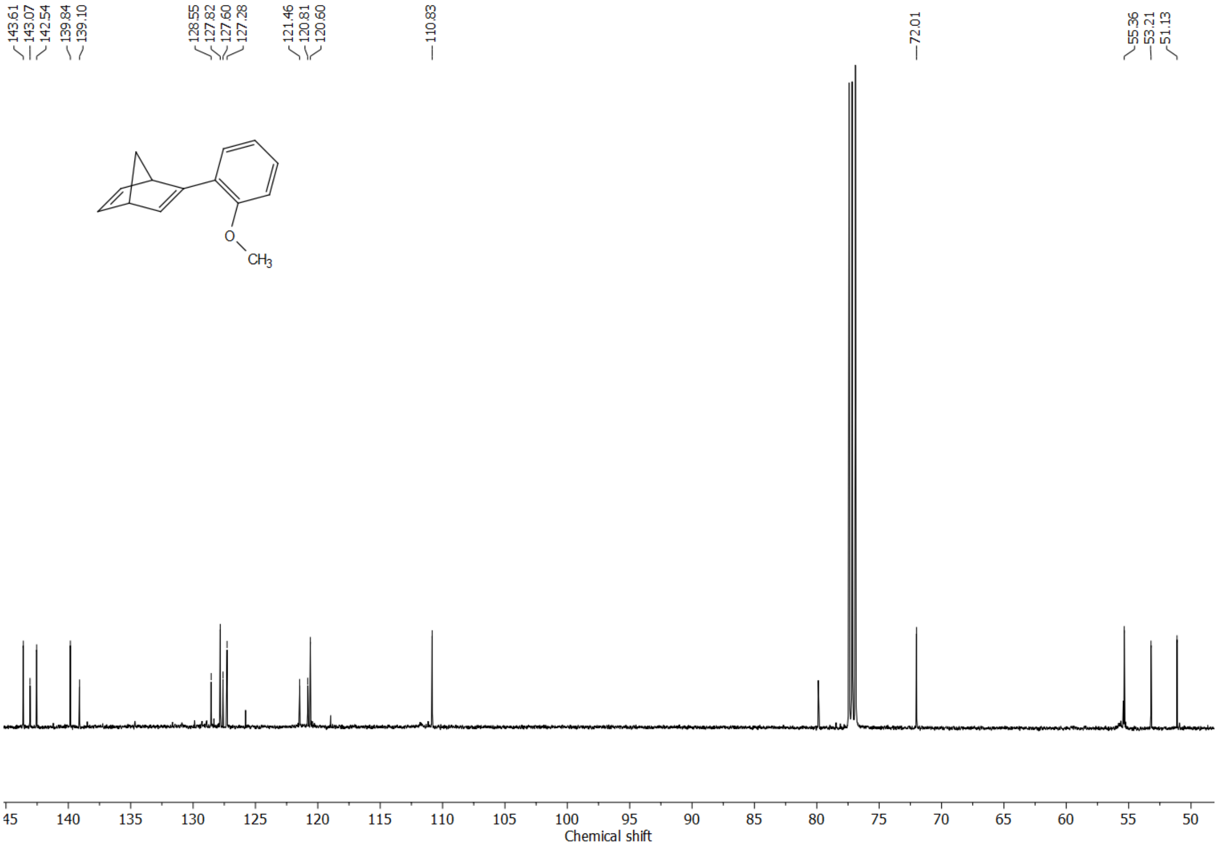

Figure S32: ^{13}C NMR spectrum of **5f** (125 MHz) in $CDCl_3$.

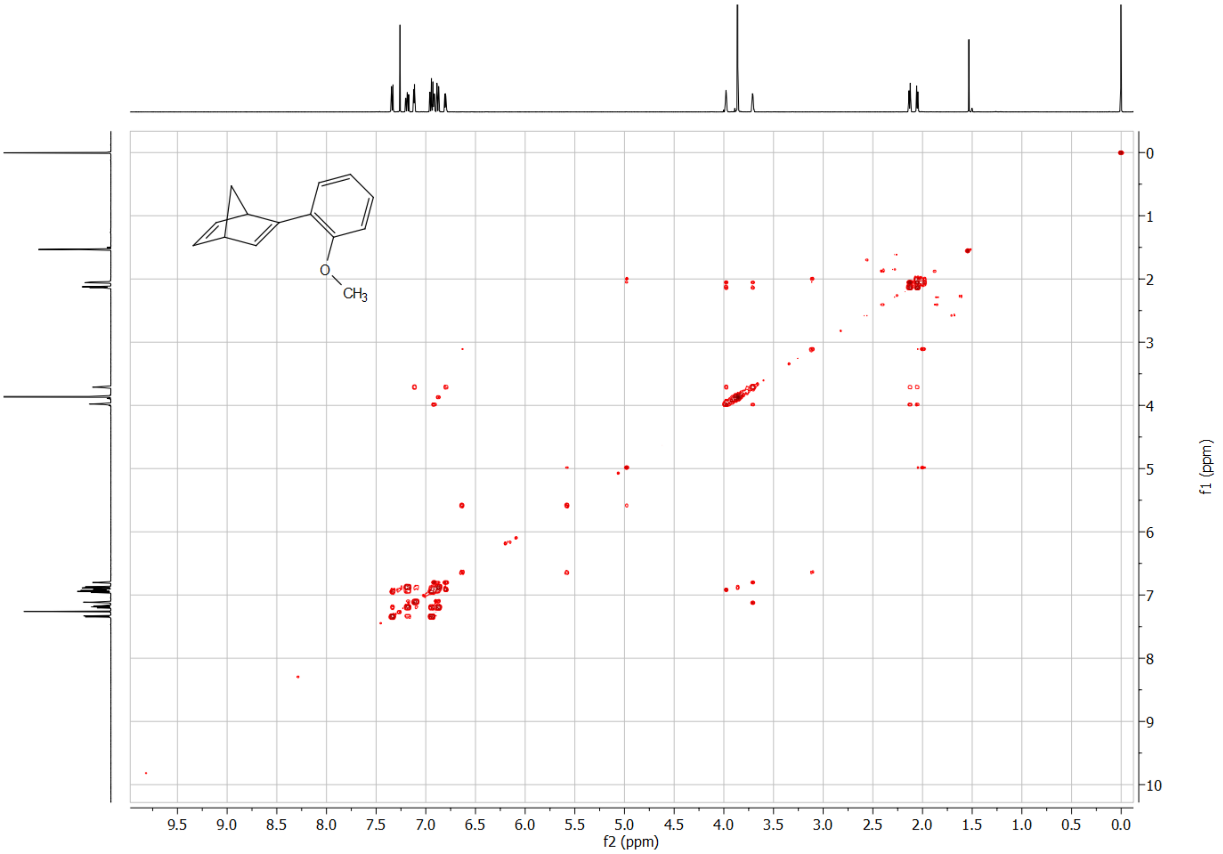

Figure S33: H,H-COSY-NMR spectrum of **5f** (500 MHz, 500 MHz) in $CDCl_3$.

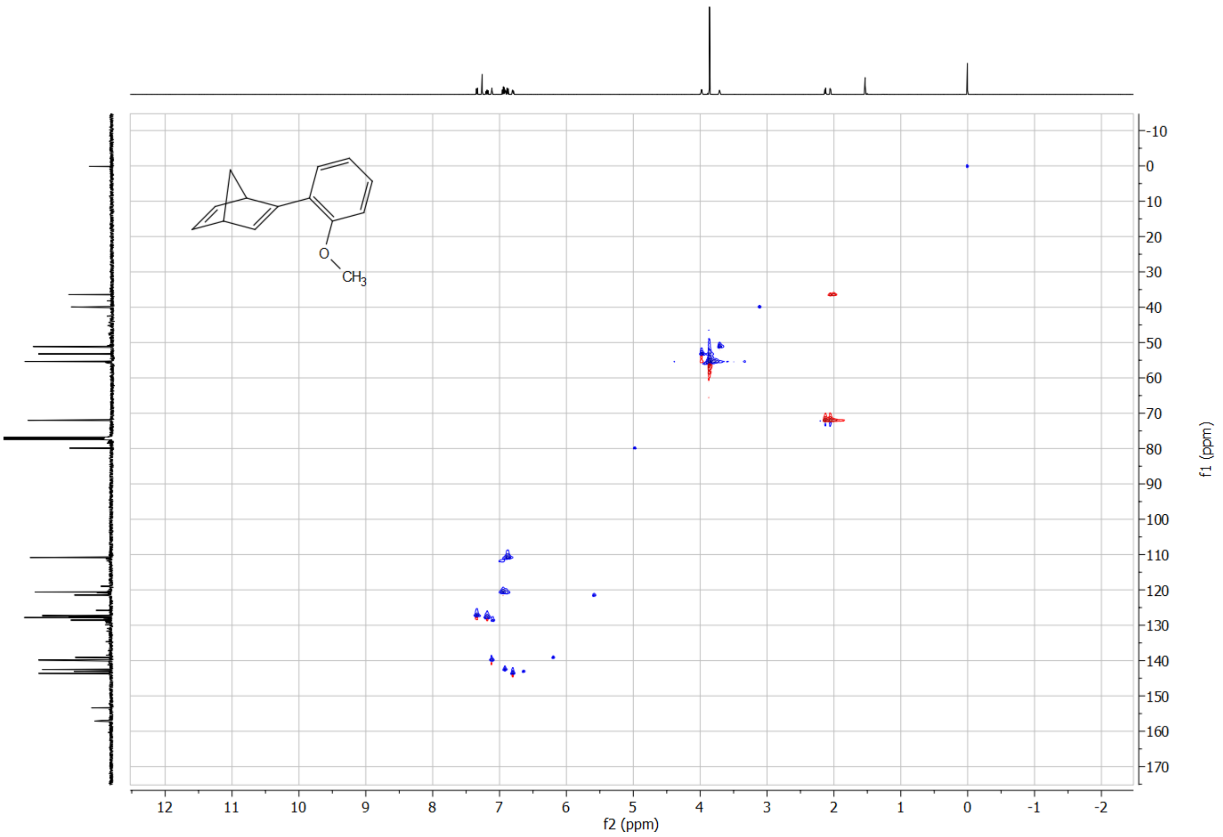

Figure S34: HSQC-NMR spectrum of **5f** (500 MHz, 125 MHz) in $CDCl_3$.

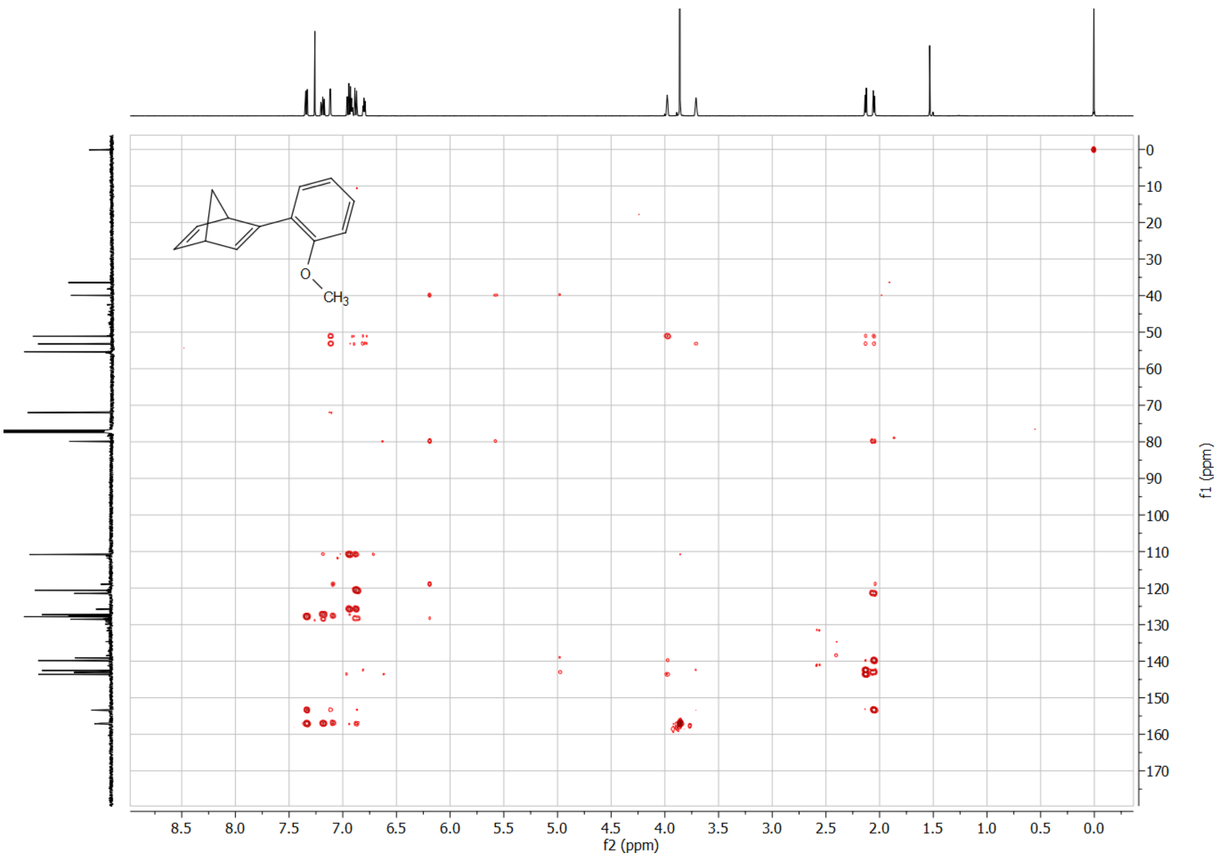

Figure S35: HMBC-NMR spectrum of **5f** (500 MHz, 125 MHz) in $CDCl_3$.

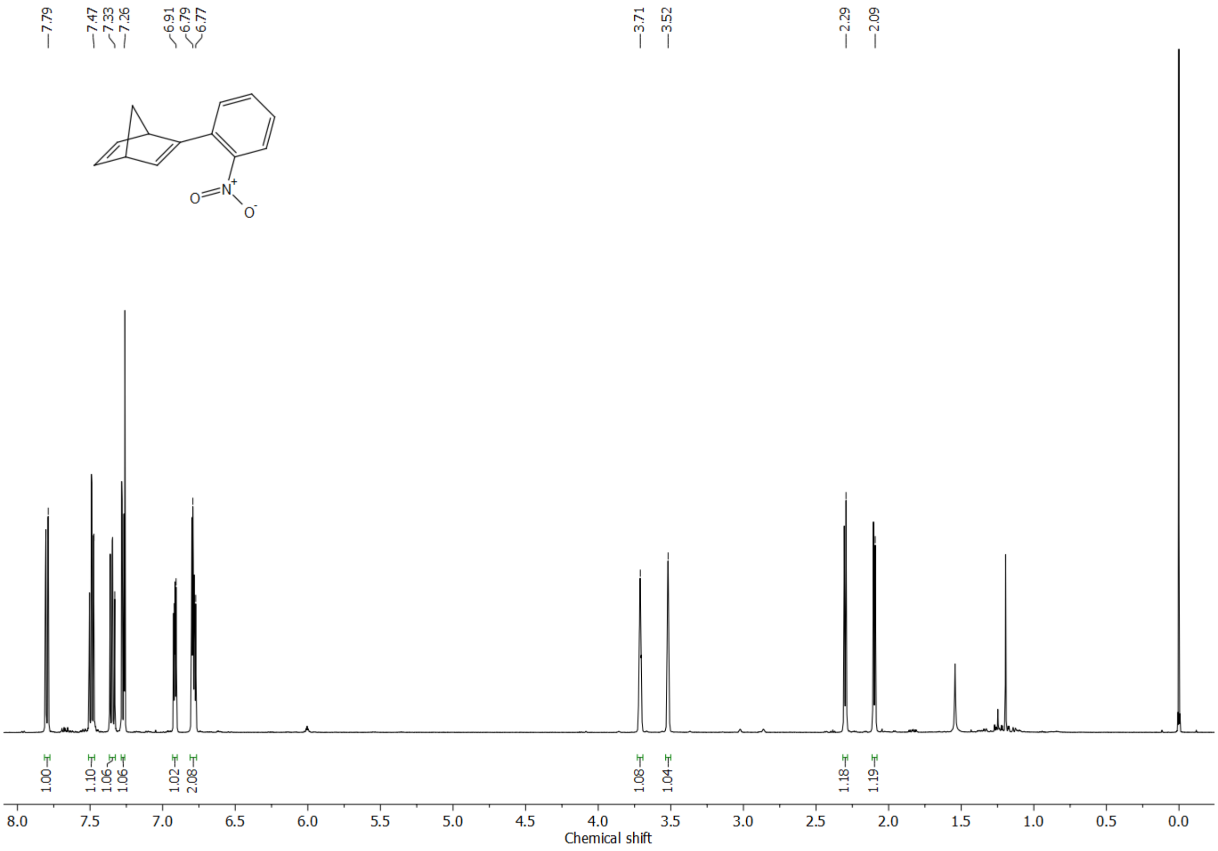

Figure S36: ^{1}H NMR spectrum of **5h** (500 MHz) in $CDCl_3$.

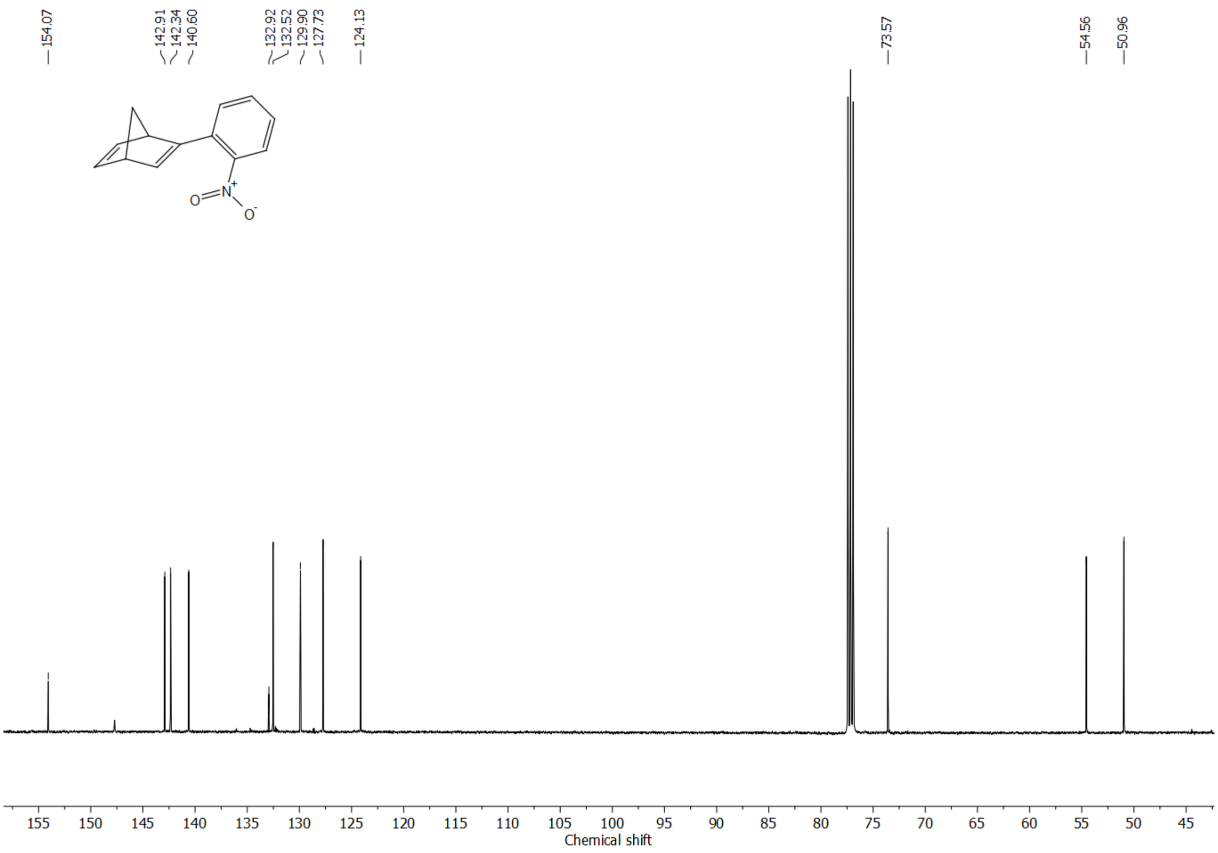

Figure S37: ^{13}C NMR spectrum of **5h** (125 MHz) in $CDCl_3$.

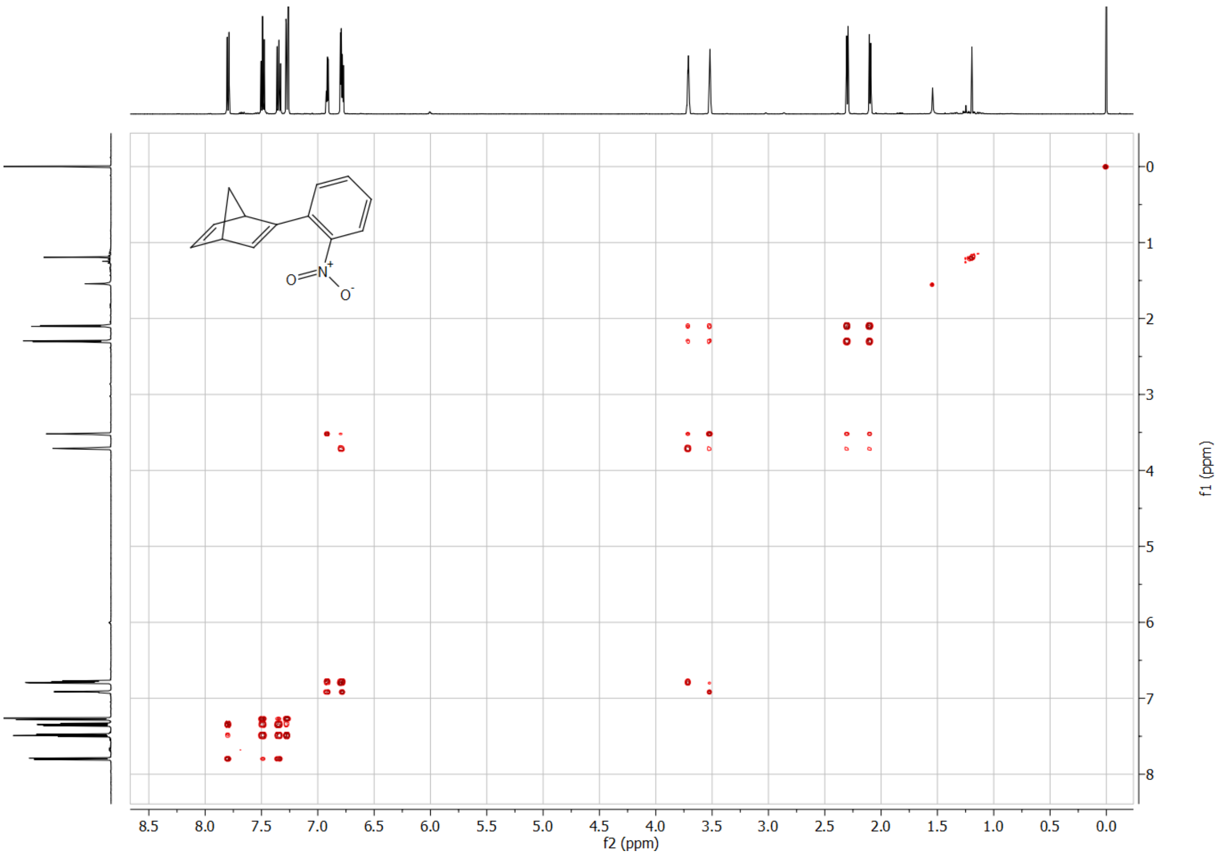

Figure S38: H,H-COSY-NMR spectrum of **5h** (500 MHz, 500 MHz) in $CDCl_3$.

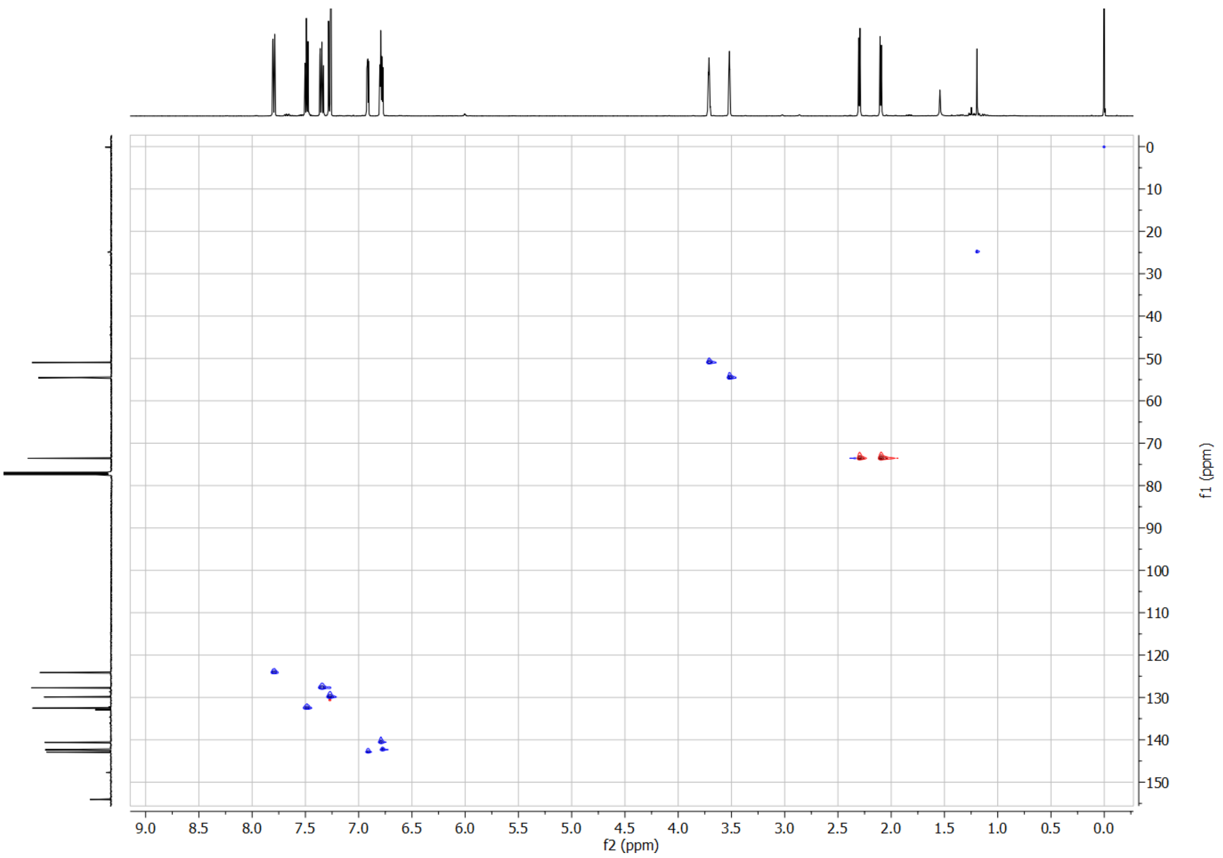

Figure S39: HSQC-NMR spectrum of **5h** (500 MHz, 125 MHz) in $CDCl_3$.

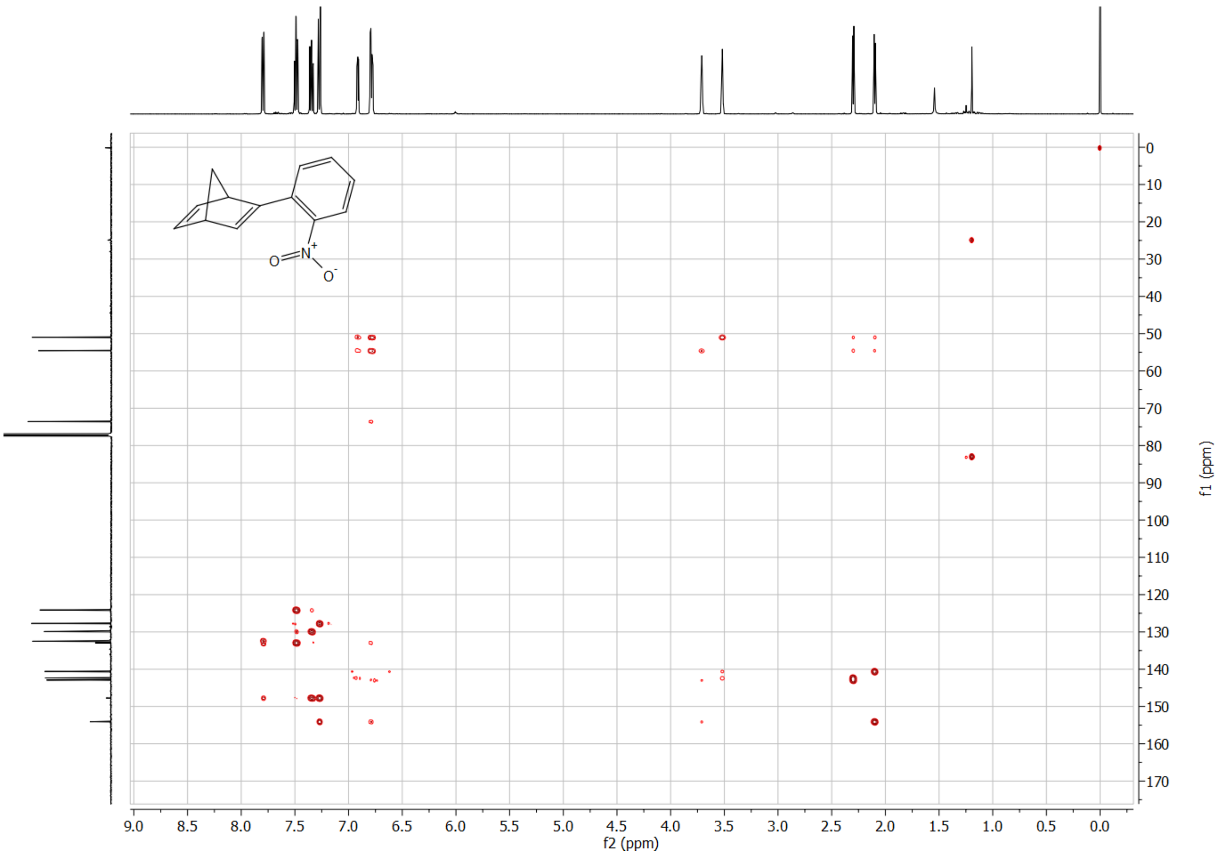

Figure S40: HMBC-NMR spectrum of **5h** (500 MHz, 125 MHz) in $CDCl_3$.

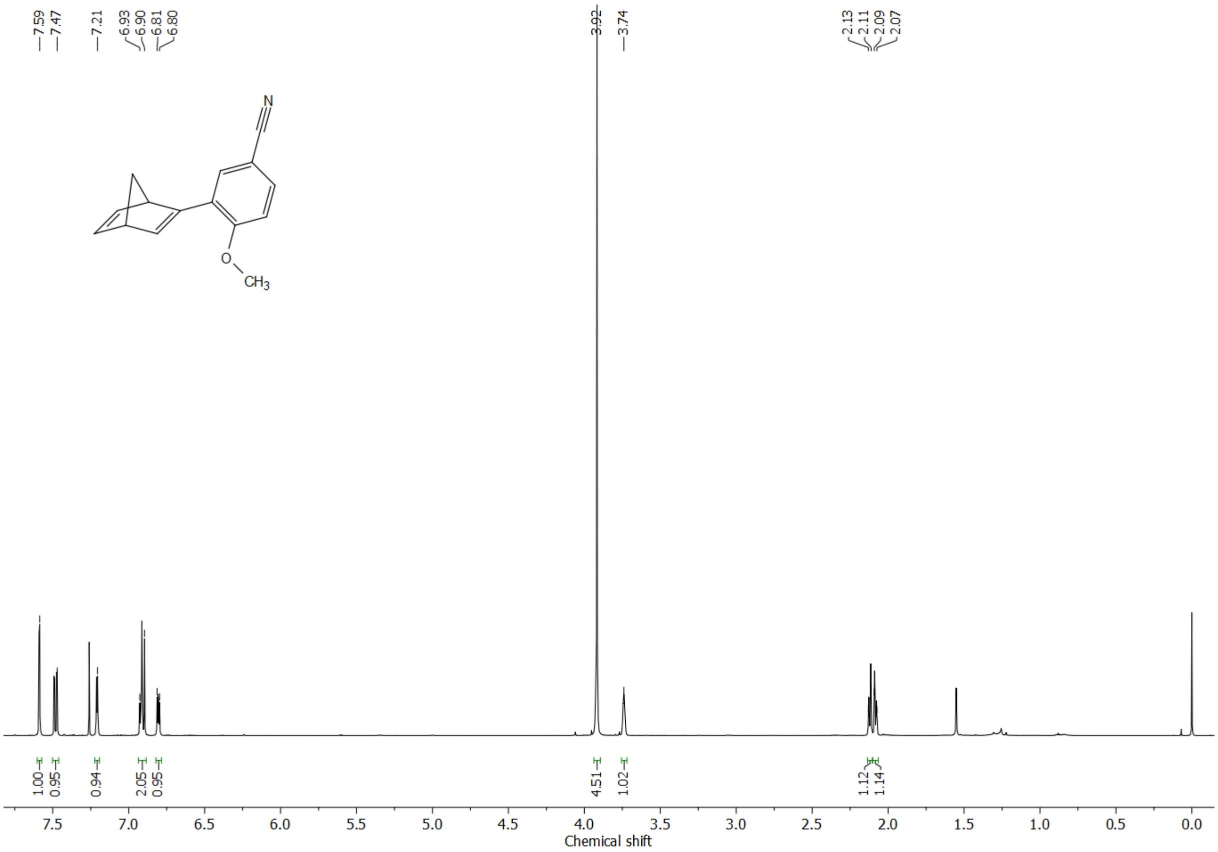

Figure S41: ^{1}H NMR spectrum of **5i** (500 MHz) in $CDCl_3$.

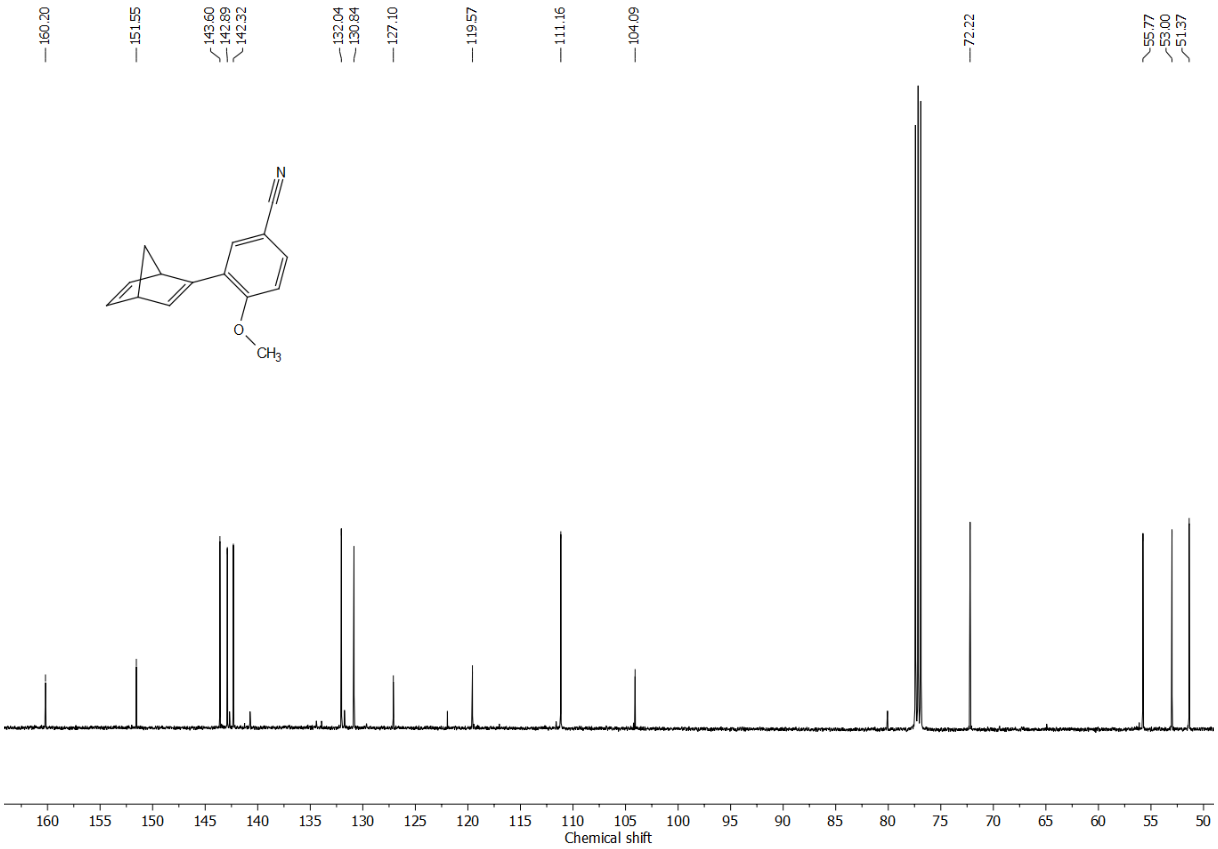

Figure S42: ^{13}C NMR spectrum of **5i** (125 MHz) in $CDCl_3$.

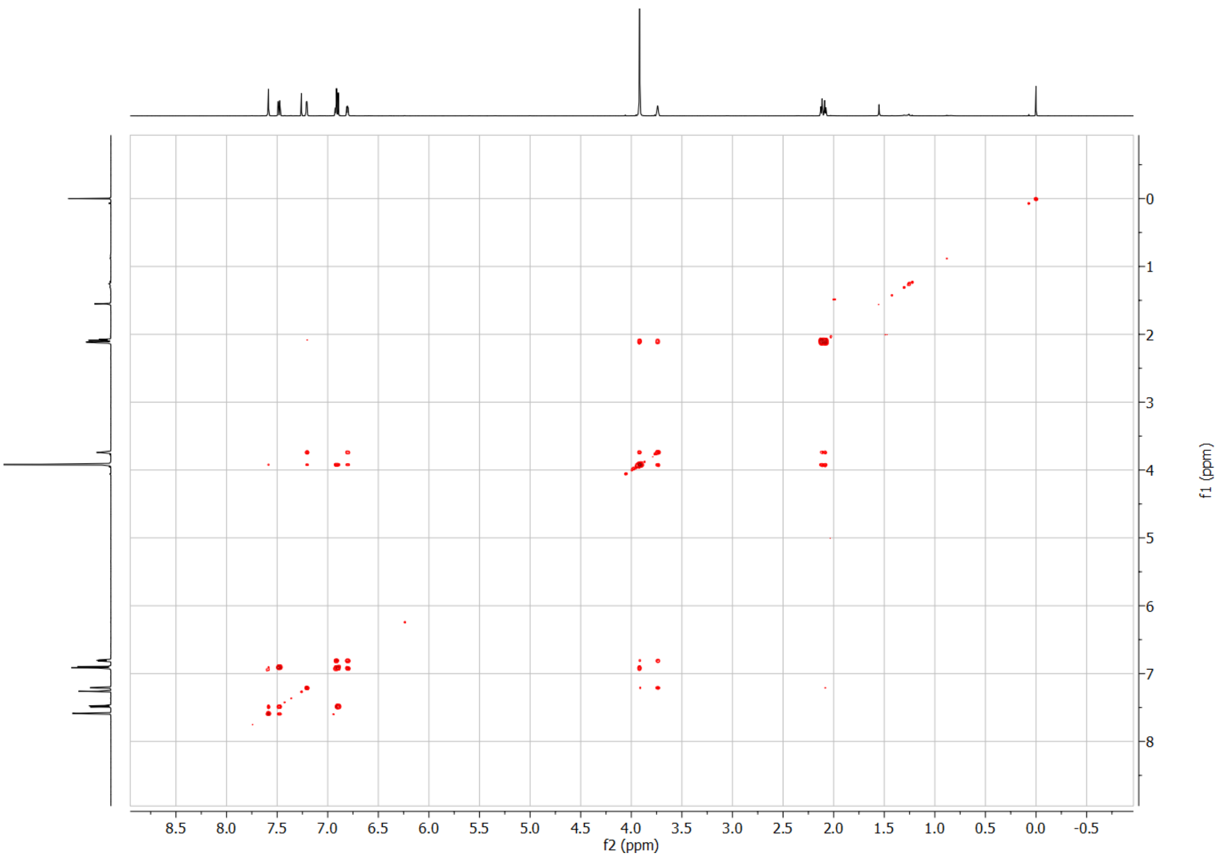

Figure S43: H,H-COSY-NMR spectrum of **5i** (500 MHz, 500 MHz) in $CDCl_3$.

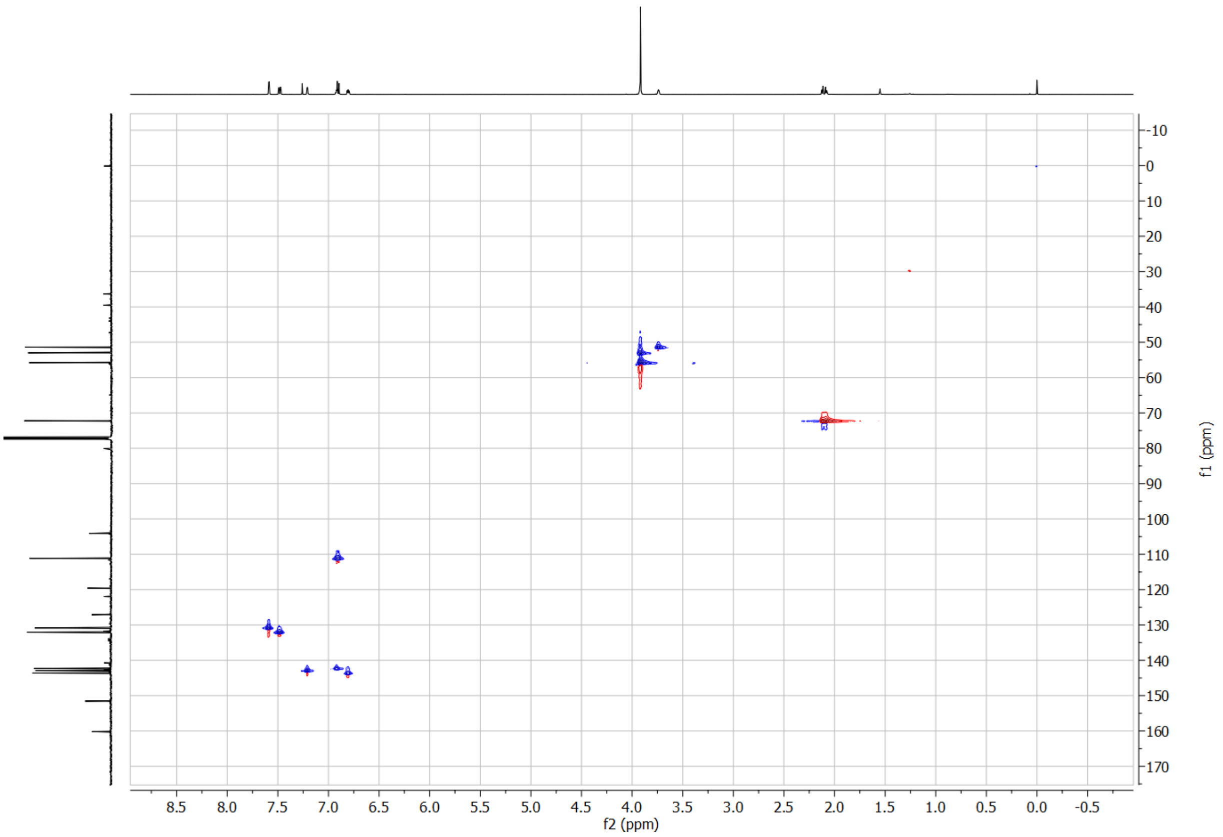

Figure S44: HSQC-NMR spectrum of **5i** (500 MHz, 125 MHz) in $CDCl_3$.

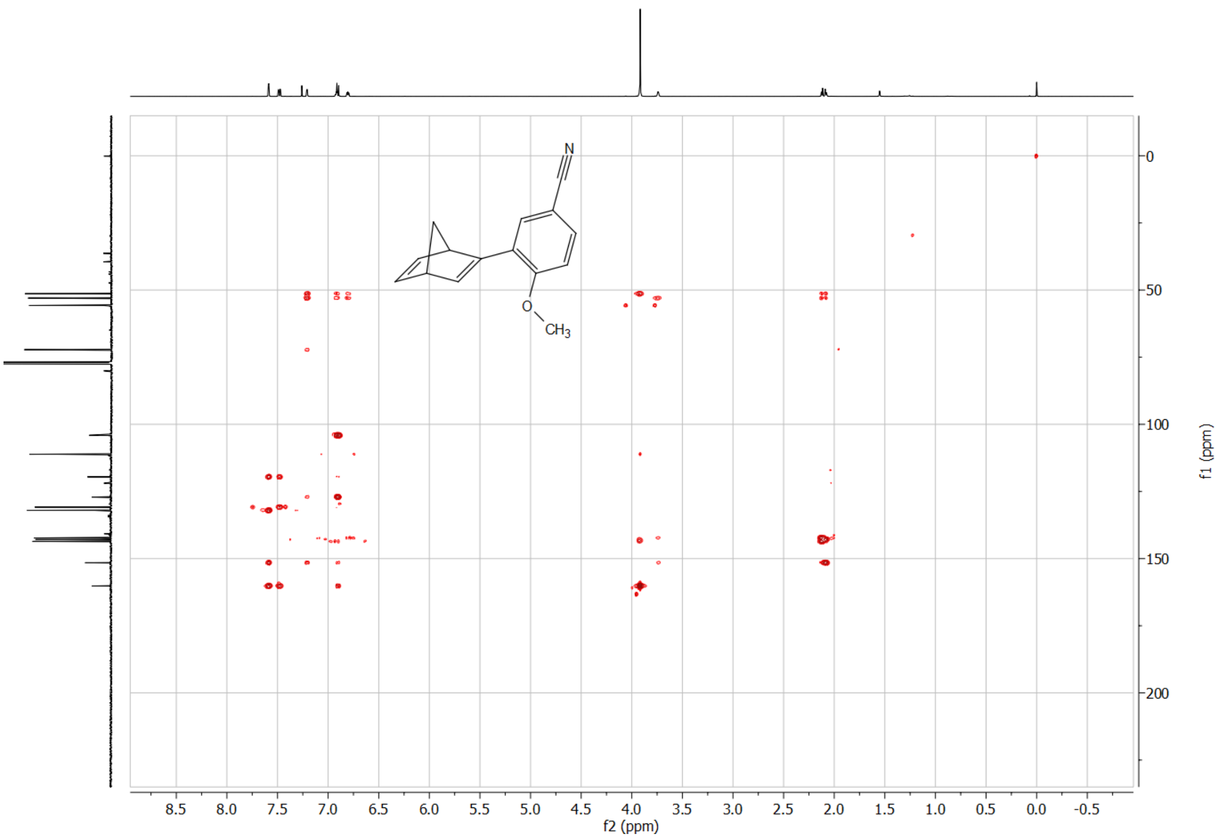

Figure S45: HMBC-NMR spectrum of **5i** (500 MHz, 125 MHz) in $CDCl_3$.

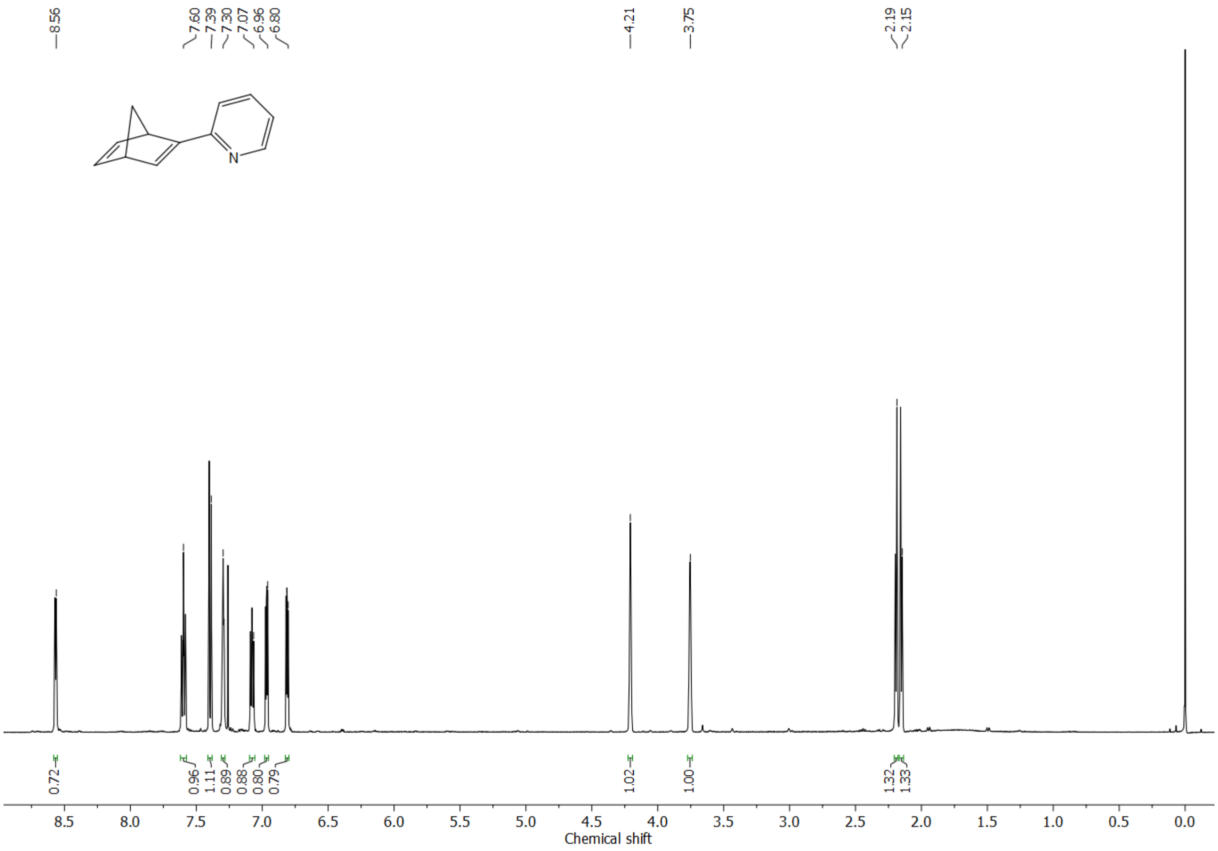

Figure S46: ^{1}H NMR spectrum of **5j** (500 MHz) in $CDCl_3$.

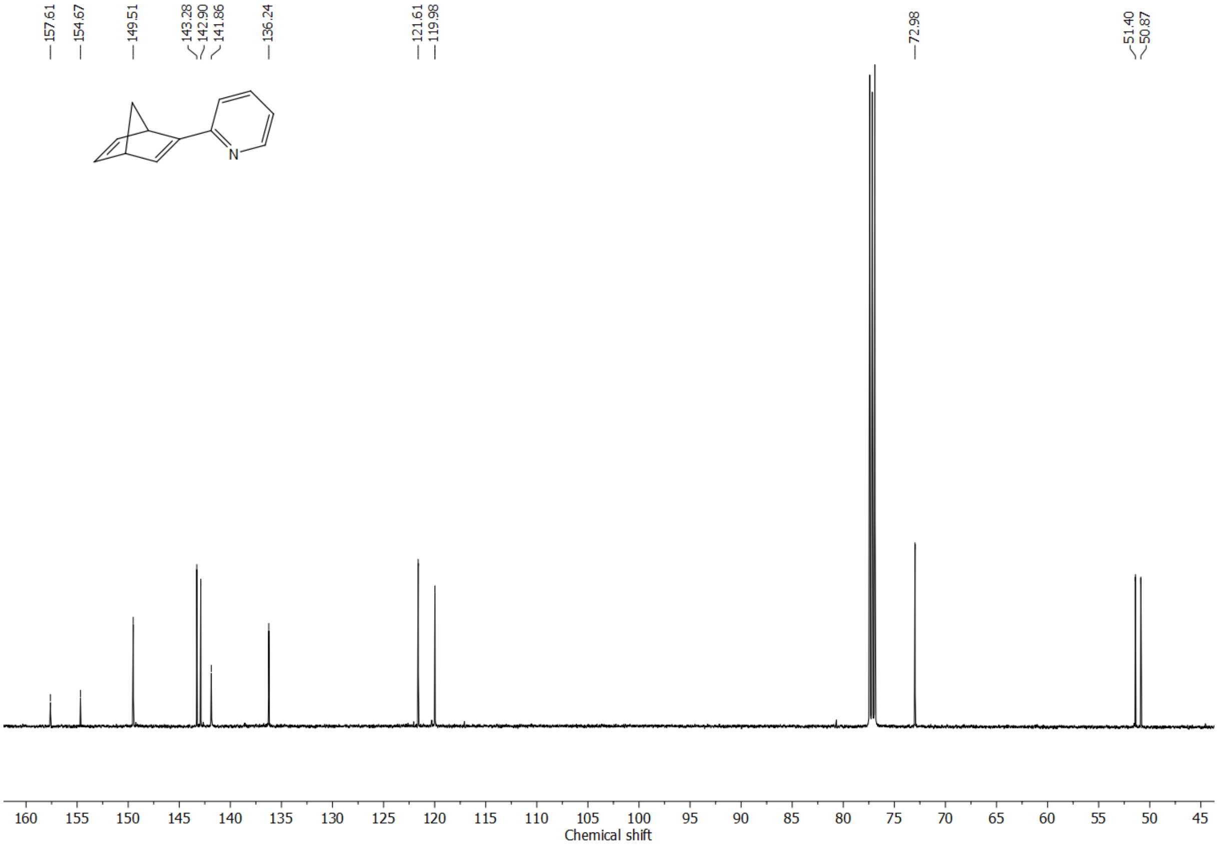

Figure S47: ^{13}C NMR spectrum of **5j** (125 MHz) in $CDCl_3$.

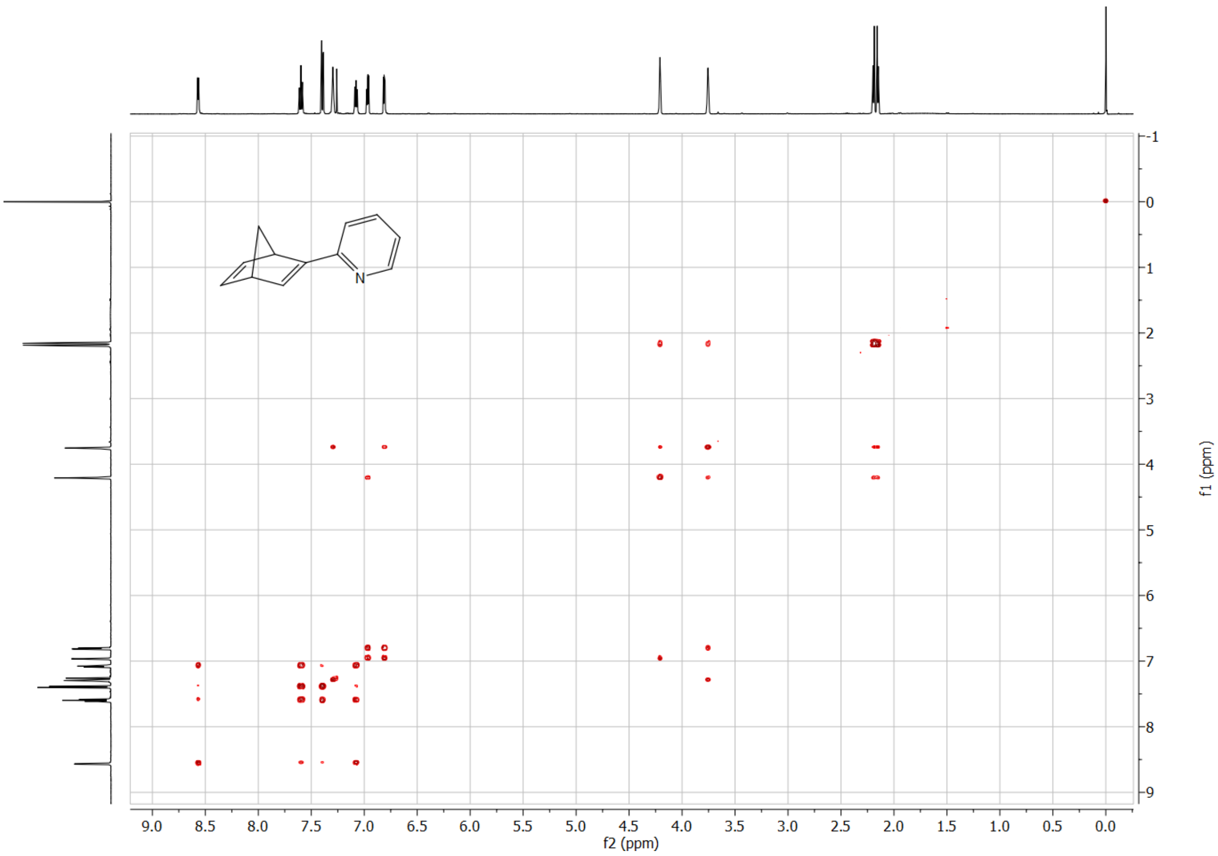

Figure S48: H,H-COSY-NMR spectrum of **5j** (500 MHz, 500 MHz) in $CDCl_3$.

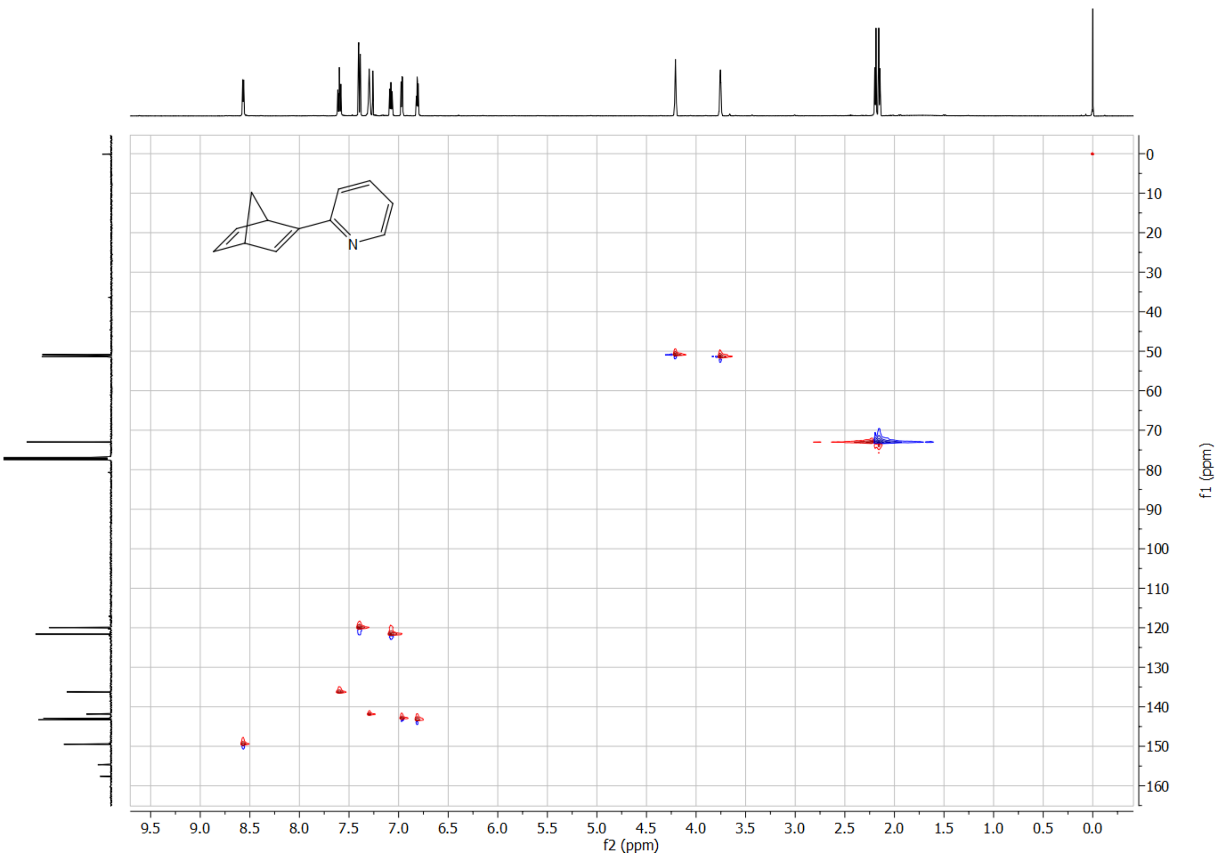

Figure S49: HSQC-NMR spectrum of **5j** (500 MHz, 125 MHz) in $CDCl_3$.

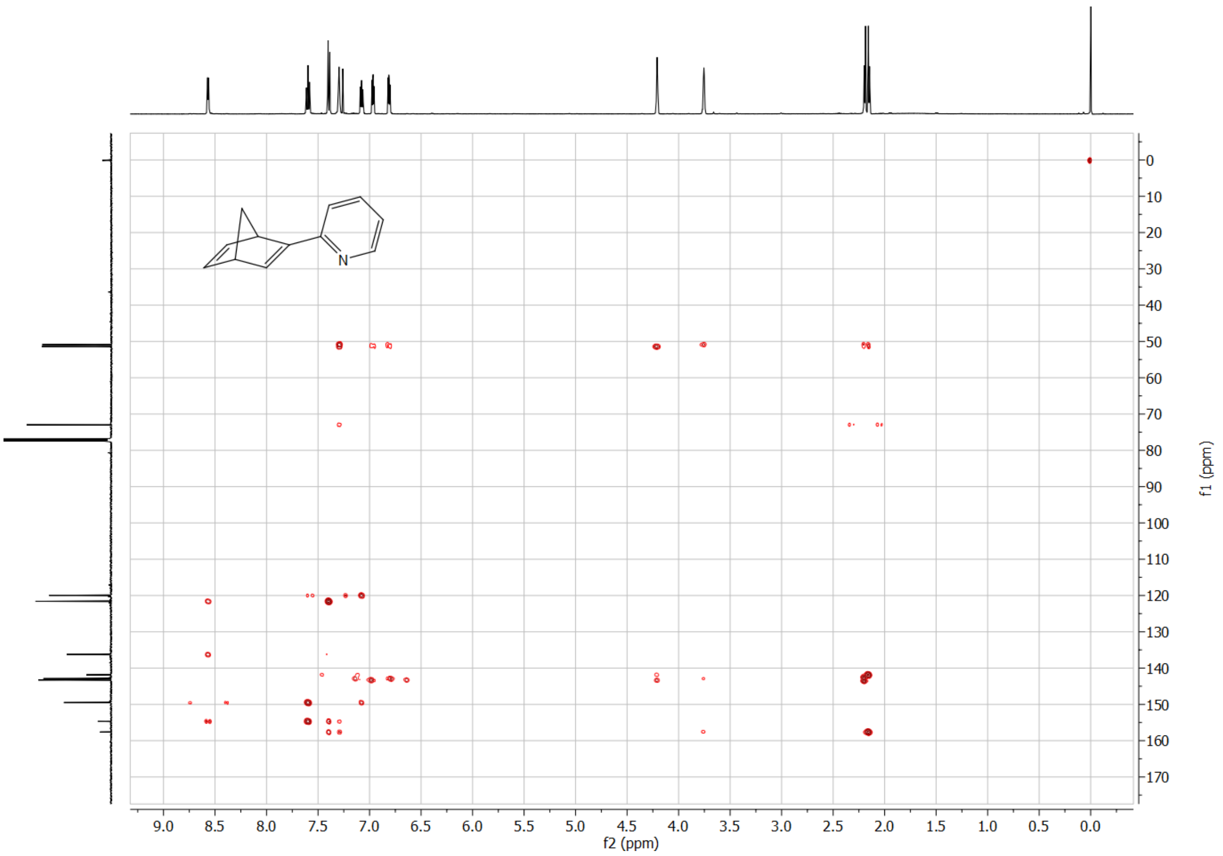

Figure S50: HMBC-NMR spectrum of **5j** (500 MHz, 125 MHz) in $CDCl_3$.

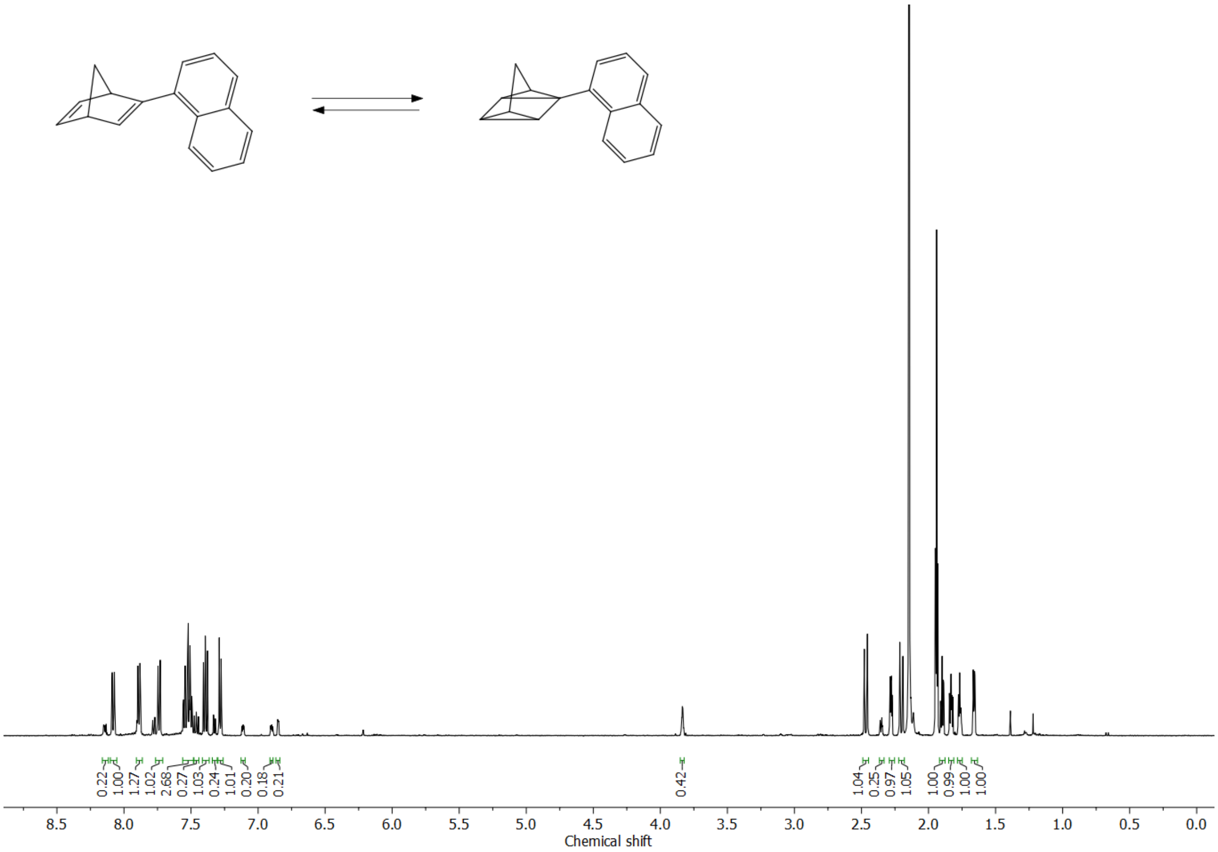

Figure S51: ^{1}H NMR spectrum (500 MHz) of the photo stationary state in CD_3CN.

3 References

1. Tranmer, G. K.; Yip, C.; Handerson, S.; Jordan, R. W.; Tam, W. *Can. J. Chem.* **2000,** *78*, 527–535.
2. Chang, C.-S.; Lin, Y.-T.; Shih, S.-R.; Lee, C.-C.; Lee, Y.-C.; Tai, C.-L.; Tseng, S.-N.; Chern, J.-H. *J. Med. Chem.* **2005,** *48*, 3522–3535.
3. Back, T. G.; Zhai, H. *Chem. Commun.* **2006**, 326–328.
4. OriginLab Corporation, OriginPro 8.5.1, Nothhampton, MA, USA, 2011.
